# Autophagy suppresses Ras-driven epithelial tumourigenesis by limiting the accumulation of reactive oxygen species

**DOI:** 10.1038/onc.2017.175

**Published:** 2017-06-05

**Authors:** J Manent, S Banerjee, R de Matos Simoes, T Zoranovic, C Mitsiades, J M Penninger, K J Simpson, P O Humbert, H E Richardson

**Affiliations:** 1 Cell Polarity and Signaling Laboratory, Department of Biochemistry and Genetics, La Trobe Institute for Molecular Science, School of Molecular Sciences, La Trobe University, Melbourne, VIC, Australia; 2 Cell Cycle and Development Laboratory, Peter MacCallum Cancer Centre, Melbourne, VIC, Australia; 3 Cell Cycle and Cancer Genetics Laboratory, Peter MacCallum Cancer Centre, Melbourne, VIC, Australia; 4 Victorian Centre for Functional Genomics, Peter MacCallum Cancer Centre, Melbourne, VIC, Australia; 5 Sir Peter MacCallum Department of Oncology, University of Melbourne, Melbourne, VIC, Australia; 6 Department of Medical Oncology, Dana-Farber Cancer Institute, Department of Medicine, Harvard Medical School, Boston, MA, USA; 7 Institute of Molecular Biotechnology, Vienna, Austria; 8 Department of Biochemistry and Molecular Biology, University of Melbourne, Melbourne, VIC, Australia; 9 Department of Pathology, University of Melbourne, Melbourne, VIC, Australia; 10 Cancer Biology, Cell Polarity and Tissue Architecture Laboratory, Department of Biochemistry and Genetics, La Trobe Institute for Molecular Science, La Trobe University, Melbourne, VIC, Australia; 11 Department of Anatomy and Neurobiology, University of Melbourne, Melbourne, VIC, Australia

## Abstract

Activation of Ras signalling occurs in ~30% of human cancers; however, activated Ras alone is not sufficient for tumourigenesis. In a screen for tumour suppressors that cooperate with oncogenic Ras (*Ras^V12^*) in *Drosophila*, we identified genes involved in the autophagy pathway. Bioinformatic analysis of human tumours revealed that several core autophagy genes, including *GABARAP*, correlate with oncogenic *KRAS* mutations and poor prognosis in human pancreatic cancer, supporting a potential tumour-suppressive effect of the pathway in Ras-driven human cancers. In *Drosophila,* we demonstrate that blocking autophagy at any step of the pathway enhances *Ras*^*V12*^-driven epithelial tissue overgrowth via the accumulation of reactive oxygen species and activation of the Jun kinase stress response pathway. Blocking autophagy in *Ras*^*V12*^ clones also results in non-cell-autonomous effects with autophagy, cell proliferation and caspase activation induced in adjacent wild-type cells. Our study has implications for understanding the interplay between perturbations in Ras signalling and autophagy in tumourigenesis, which might inform the development of novel therapeutics targeting Ras-driven cancers.

## Introduction

Mutations that lock the small G-protein RAS in its activated form, such as RAS^V12^, are found in over 30% of human cancers, making it one of the most important oncogenes.^1, 2, 3^ However, RAS activation alone is not sufficient for the development of cancer because of the induction of senescence.^4, 5, 6, 7, 8^ Identification of genes or pathways that cooperate with Ras to drive cancer progression is therefore paramount. With its ease of manipulation and expansive repertoire of available genetic tools, the vinegar fly *Drosophila melanogaster* has served a pioneering role in the study of cancer-causing genes and cooperative tumourigenesis.^9, 10, 11^ In Ras-driven epithelial overgrowth, studies in *Drosophila* have revealed that impairment of cell polarity or mitochondrial functions enhance tumour growth and invasion via activation of the Jun kinase (JNK) stress response pathway, faithfully recapitulating some of the features responsible for tumour progression in human cancers.^11, 12, 13, 14, 15^

Autophagy is a widely conserved catabolic process, and basal autophagy levels are necessary for cell homeostasis, clearing aberrant or unnecessary cytoplasmic material, such as misfolded proteins, supernumerary or defective organelles, the accumulation of which would otherwise generate toxic stress that is detrimental to proper cell metabolism and function.^16, 17^ In some developmental contexts, autophagy has also been shown to induce programmed cell death.^18^ Indeed, there is a tight relationship between autophagy and apoptosis, as members of the BCL-2 antiapoptotic family of protein interact directly with the autophagy machinery at the nucleation step.^19^ Moreover, autophagy is induced in response to external stresses, such as starvation or hypoxia, allowing cells to cope with transient nutrient deprivation or lower levels of oxygen.^20^

In tumourigenesis, the role of autophagy is yet to be fully resolved and appears context dependent.^21, 22, 23^ Several lines of evidence point to a role for autophagy in the survival of tumour cells in the hypoxic, nutrient-deprived microenvironment of an early tumour where a blood supply is yet to be established. Indeed, higher autophagy levels are present in most aggressive forms of human cancers to sustain the growth of the tumour, and blocking autophagy in mouse models of cancer restrains growth and progression towards more aggressive types of tumours.^24, 25^ Of note, tumours with activating *Ras* mutations are particularly dependent on functional autophagy.^24, 26, 27, 28, 29^ Conversely, inactivating mutations in several autophagy genes, such as *UVRAG*, *AMBRA1* and *BECN1*, have been identified in breast and ovarian cancers,^30^ and *Becn1*, *Atg5* or *Atg7* depletion in the mouse are linked to increased tumourigenesis, highlighting the tumour-suppressive function of autophagy in these contexts.^31, 32^

In this study, following our identification of the core autophagy regulator *Atg8a*/*GABARAP*, *Atg7* and *Atg9* in a large-scale screen for *Ras*^*V12*^-cooperating tumour suppressors in *Drosophila*, we investigate the tumour-suppressive role of autophagy in Ras-activated tissues. We show that knockdown of autophagy at any step of the process synergises with oncogenic Ras to promote growth of *Drosophila* epithelial tissues. We bioinformatically identify low expression of various autophagy genes in pancreatic adenocarcinoma and correlate it with oncogenic *KRAS* status and poor prognosis. In *Drosophila*, we uncover non-cell-autonomous proliferation and apoptosis in wild-type tissue surrounding *Ras*^*V12*^-autophagy-impaired mutant clones. We show that autophagy inhibition in a Ras-activated background leads to induction of oxidative stress and JNK pathway activation. Genetic knockdown of oxidative stress or JNK is sufficient to prevent *Ras*^*V12*^-autophagy-impaired tissue overgrowth. Our study reveals extensive cross-talk between Ras signalling and the autophagy pathway and suggests that therapeutic inhibition of autophagy could be contraindicated in certain subsets of cancers.

## Results

### A screen for *Ras^V12^*-cooperative tumour suppressors identifies genes involved in the autophagy pathway

**Figure 1:**
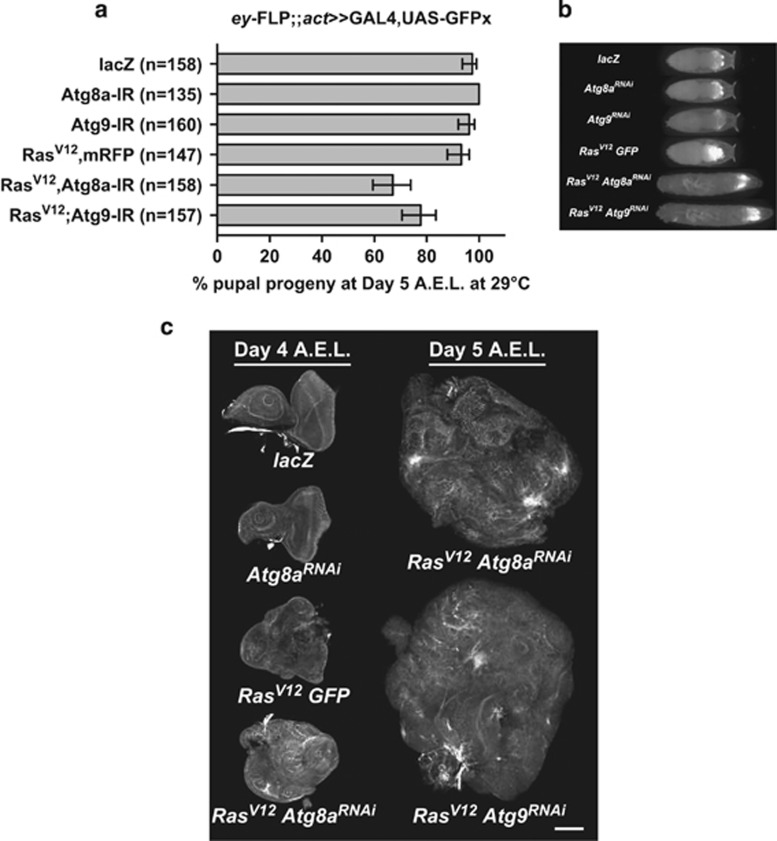
A screen for Ras-cooperative tumour suppressor genes identifies members of the autophagy pathway. (**a**) Pupariation count of *ey-FLP-out Act»GAL4* larvae expressing the indicated transgenes at 5 days AEL. Knockdown of *Atg8a* or *Atg9* in combination with *Ras*^*V12*^ expression delayed pupariation in ~20% of animals, while neither single knockdown of *Atg8a* or *Atg9*, nor expression of *Ras*^*V12*^ alone, did. (**b**) At day 5 AEL, giant larvae with enhanced GFP-positive masses can be observed in *Ras*^*V12*^ *Atg8a*^*RNAi*^ or *Ras*^*V12*^ *Atg9*^*RNAi*^ crosses by fluorescent microscopy, while all control animals have turned into pupae. (**c**) F-actin (phalloidin) staining of eye-antennal discs of *ey-FLP-out Act»GAL4* wandering L3 larvae at day 4 AEL or giant larvae at day 5 AEL expressing the indicated transgenes. *Ras*^*V12*^ *Atg*^*RNAi*^ tissues lose morphology and keep growing after all control flies have pupariated. Error bars=95% confidence intervals. Scale bar: 50 μm.

To identify new tumour suppressor genes that cooperate with constitutively active oncogenic *Ras* (*Ras^V12^*), a high-throughput genome-wide *in vivo* RNA interference (RNAi) screen in the *Drosophila* eye-antennal epithelium was performed (Zoranovic *et al.*, in preparation). Hits were selected based on their ability to delay larval development when knocked down in eye-antennal imaginal discs, a characteristic that correlates with uncontrolled growth of the tissue and subsequent failure to timely induce pupariation.^33, 34^ Three genes of the autophagy pathway, *Atg7*, *Atg8a* and *Atg9*, were identified among the hits, suggesting that autophagy can suppress the growth of *Ras*^*V12*^-expressing epithelial tissue. Indeed, knockdown of *Atg8a or Atg9* in *Ras*^*V12*^-expressing eye-antennal epithelial tissue using the *eyeless-FLP-out, actin-GAL4* system (see Materials and methods) delayed pupariation, with 20% of animals still crawling as giant larvae 5 days after egg lay (AEL) when raised at 29 °C, whereas controls formed pupae at 4 days (Figures 1a and b). Third instar larval *Ras*^*V12*^ *Atg*^*RNAi*^ eye-antennal epithelial tissues displayed loss of architecture and overgrowth compared with control discs (Figure 1c). Furthermore, *Ras*^*V12*^ *Atg*^*RNAi*^ eye-antennal epithelial tissue overgrew (Figure 1c—day 5 AEL) and was associated with a giant larvae phenotype, when all control flies had pupariated (Figures 1a and b). Notably, knocking down *Atg8a* or *Atg9* alone did not induce any significant defect in size or shape of the eye-antenna discs nor delayed pupariation (Figures 1a–c, and data not shown), and animals developed into healthy, fertile adults (Supplementary Figure 1a). Thus, knockdown of *Atg8a* or *Atg9* cooperates with *Ras*^*V12*^ to result in neoplastic tumours and blocks metamorphosis at the larval–pupal stage, a characteristic observed previously with other Ras-cooperating mutations, for example, in the apicobasal polarity genes, *scrib*, *dlg*, *lgl* and *baz*,^12, 13, 35^ or upon overexpression of actin cytoskeletal genes, *RhoGEF2, pbl* (RhoGEF), *Rac1* and *bsk* (Jun kinase).^36, 37^

### All steps of autophagy are required to limit overgrowth of *Ras^V12^*-expressing epithelial tissues

**Figure 2:**
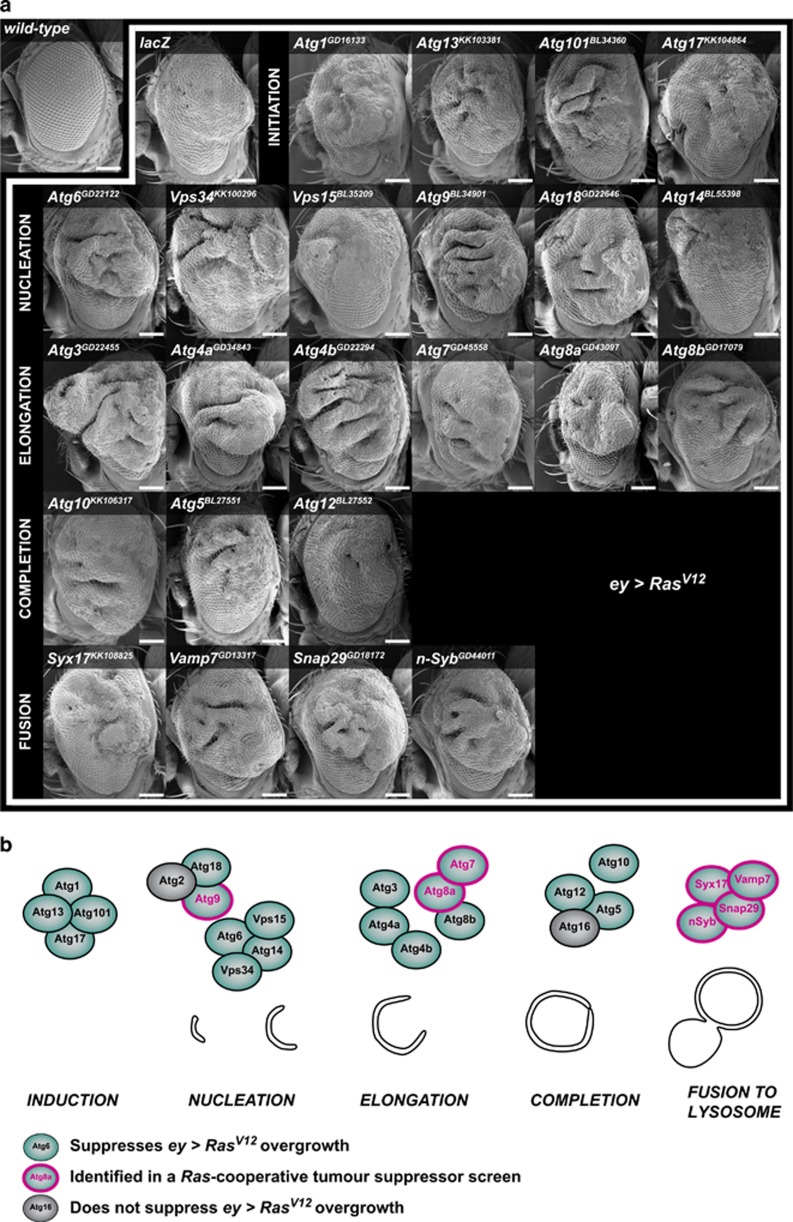
Impaired autophagy enhances Ras-driven overgrowth of the adult *Drosophila* eye. (**a**) Scanning electron micrographs of *ey*-*GAL4* adult female *Drosophila* eyes crossed to the indicated RNAi lines (superscript) or *lacZ* control. Inhibition of autophagy at any step of the process results in enhanced overgrowth of *Ras*^*V12*^-expressing eyes. (**b**) Schematic of the autophagy pathway. Genes that knockdown enhances Ras-driven overgrowth are highlighted in green, with those identified in the *Ras*^*V12*^-cooperative tumour suppressor primary screen circled in red. Scale bars: 100 μm.

Because the three autophagy-related genes identified in our screen mapped to two distinct steps of the process—formation of the isolation membrane for *Atg9*, or phagophore elongation for *Atg7* and *Atg8a*, we questioned whether the tumour-suppressive effect was limited to these genes, or whether autophagy in general was necessary to limit *Ras*^*V12*^-driven overgrowth of fly epithelial tissue. To test this systematically, we switched to a well-characterised tissue overgrowth model in which the *eyeless*-*GAL4* (*ey-GAL4*) driver expresses *Ras*^*V12*^ and/or *Atg-*RNAis in the eye-antennal epithelium.^36, 38^ Knockdown of *Atg8a*, *Atg7* or *Atg9* each enhanced the overgrowth and roughness of *ey-GAL4, Ras*^*V12*^ (*ey*>*Ras*^*V12*^) eyes, as anticipated (Figure 2a and Supplementary Figure 2a), which was not observed with the controls (Supplementary Figure 2b). We then assessed the growth of adult eyes expressing *Ras*^*V12*^ and RNAis for most of the documented genes involved in autophagy in *Drosophila* (Table 1, Supplementary Table 1 and Supplementary Figure 2a), and confirmed that the RNAi lines could effectively block autophagy in a Ras-activated developing epithelium by measuring accumulation of the autophagic cargo adapter Ref(2)P/p62 (Supplementary Figure 3). This included genes required at the initiation (*Atg1*, *Atg101*, *Atg13*, *Atg17*), nucleation (*Atg6*, *Vps34*, *Vps15*, *Atg14*, *Atg18a*), elongation (*Atg3*, *Atg4a*, *Atg4b*, *Atg8b*) and completion (*Atg5*, *Atg10*, *Atg12*) steps. Strikingly, knockdown of almost all genes involved in the autophagy pathway enhanced *Ras*^*V12*^-induced overgrowth (Figures 2a and b, Supplementary Figure 2a and Supplementary Table 1). The only exceptions were that of *Atg2* and *Atg16*, for which the single RNAi line available for each did not modify *ey*>*Ras*^*V12*^ eye size or roughness (Supplementary Figure 2a), and *Atg18b*, which we did not assess in this study.

These data suggested that other autophagy-related hits might be present in our screen. Interestingly, analysis of protein interactions within the 947 candidate genes identified in the *Ras*^*V12*^-cooperative tumour suppressor screen revealed a cluster of Snap/SNARE proteins, composed of Syx17, Snap29 and Vamp7^39^ (Supplementary Figure 1b), that have recently been shown to regulate autophagy at the last step of the process by promoting fusion of autophagosomes to lysosomes.^40^ Of interest, the neuronal synaptobrevin, *nSyb*, predicted by protein interaction studies to be part of this cluster,^39^ and whose paralogue Syb was recently confirmed to form a functional complex with Syx17, Snap29 and Vamp7,^41^ was also identified in our screen. Similarly to other components of the autophagy pathway above, we were able to confirm that *Syx17*, *Vamp7*, *Snap29* or *nSyb* knockdown could also enhance *Ras*^*V12*^-induced overgrowth (Figures 2a and b, Supplementary Figure 2a and Supplementary Table 1).

Taken together, our data show that blocking autophagy at any step of the pathway can potentiate Ras-driven overgrowth (Figure 2b), suggesting that functional basal autophagy is required to limit overgrowth of *Ras*^*V12*^-expressing epithelial tissues.

#### Lower expression of *GABARAP* and *VAMP2* correlates with worse prognosis in KRAS-G12-positive pancreatic adenocarcinoma

**Figure 3:**
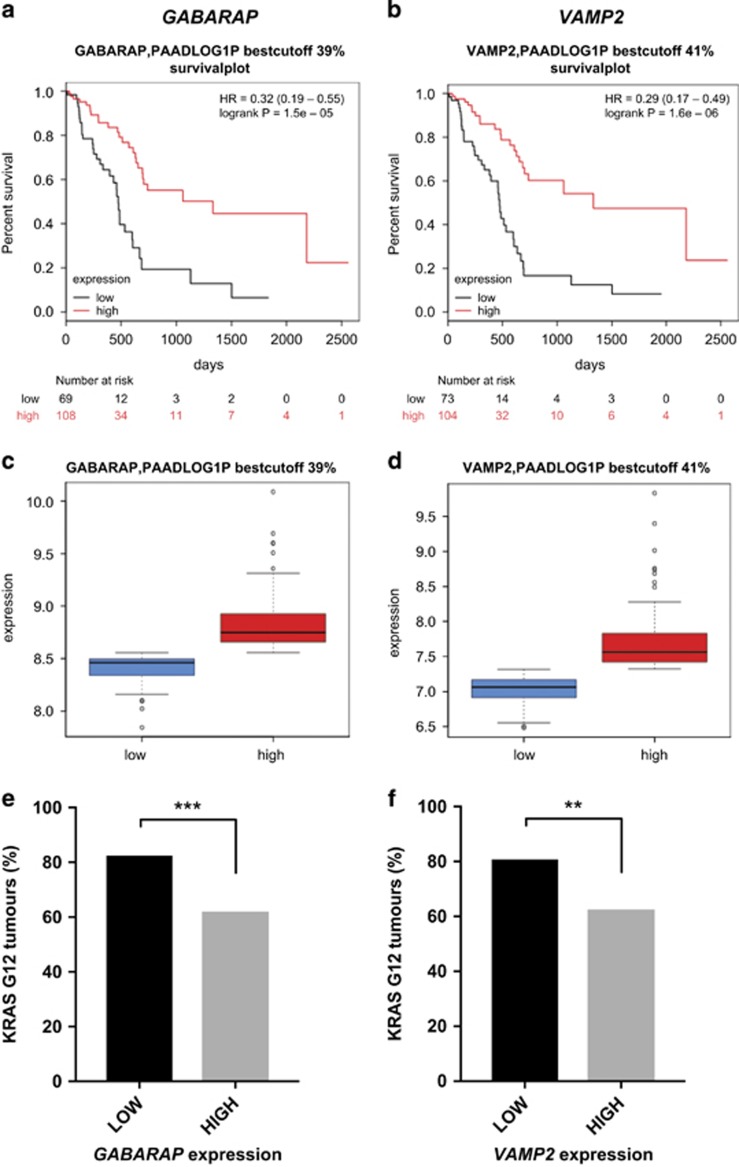
Low expression of *GABARAP* and *VAMP2* is prognostic for worse survival in KRAS-G12-positive PAAD. Survival curves of PAAD patients with low (black) or high (red) expression of *GABARAP* (**a**) and *VAMP2* (**b**). Low expression of *GABARAP* (**c**) or *VAMP2* (**d**) correlates with worse clinical outcome in patients (**a** and **b**) and with enrichment for G12-activating mutations in KRAS (**e** and **f**), suggesting that these autophagy-related genes behave as tumour suppressors in PAAD with KRAS-activating mutations. Statistics: Fisher’s exact *t*-test.

The autophagy pathway has ambivalent roles in tumourigenesis (reviewed in Ávalos *et al.*^22^). Our *Drosophila* data argues for a tumour-suppressive function of the pathway in Ras-driven tumourigenesis. To investigate the expression of autophagy-related genes in human tumours with documented *KRAS* mutations, we queried available RNAseq data from The Cancer Genome Atlas. We focused on pancreatic ductal adenocarcinoma (PAAD), as incidence of *KRAS*-activating mutations in this tumour type is above 80%. We performed K-means clustering of tumours into low- or high-expressing groups for each of the human orthologues of the autophagy genes identified in the fly screen, and survival distribution was assessed for both groups. Patients with tumours exhibiting low mRNA expression for *GABARAP*, *GABARAPL1*, *GABARAPL2*, *GABARAPL3* or *VAMP2* showed significant association with worse clinical outcome (hazard ratio <1) and significant enrichment for *KRAS-G12*-activating mutations (*P*<0.01) (Figures 3a–d, Supplementary Table 2 and Supplementary Data sets 1 and 2), suggesting that in PAAD with *G12 KRAS* mutations, these genes individually behave as tumour suppressors. Noteworthy, this correlation between low-expressing autophagy, *KRAS*-activating mutations and poor clinical outcome was still observed when tumours were ranked for the mean expression of the entire subset of autophagy genes identified in the primary fly screen (Supplementary Figure 4), although this effect was mostly, but not exclusively, driven by the *GABARAP* family and *VAMP2* (Supplementary Table 3).

On the other hand, poorer clinical outcome was associated with higher expression of other autophagy-related genes such as *VAMP7* and *VAMP8* (Supplementary Table 2 and Supplementary Data sets 1 and 2). While this suggests that not all genes of the pathway act as tumour suppressors, in tumours where *VAMP7* or *VAMP8* showed higher expression, other autophagy genes, such as *ATG9B* or *GABARAP*, often showed reduced expression (Supplementary Figure 4a and Supplementary Data set 2), and this upregulation could reflect a mechanism to compensate for a blockage of the pathway at another level.

Altogether, this analysis shows that low expression of the *GABARAP* family and *VAMP2* correlates with a higher risk of poorer outcome in PAAD and might serve as a good indicator of poor prognosis in *KRAS-G12* bearing tumours.

### Autophagy limits the growth of *Ras^V12^ dlg^RNAi^* epithelial tumours in *Drosophila*

**Figure 4:**
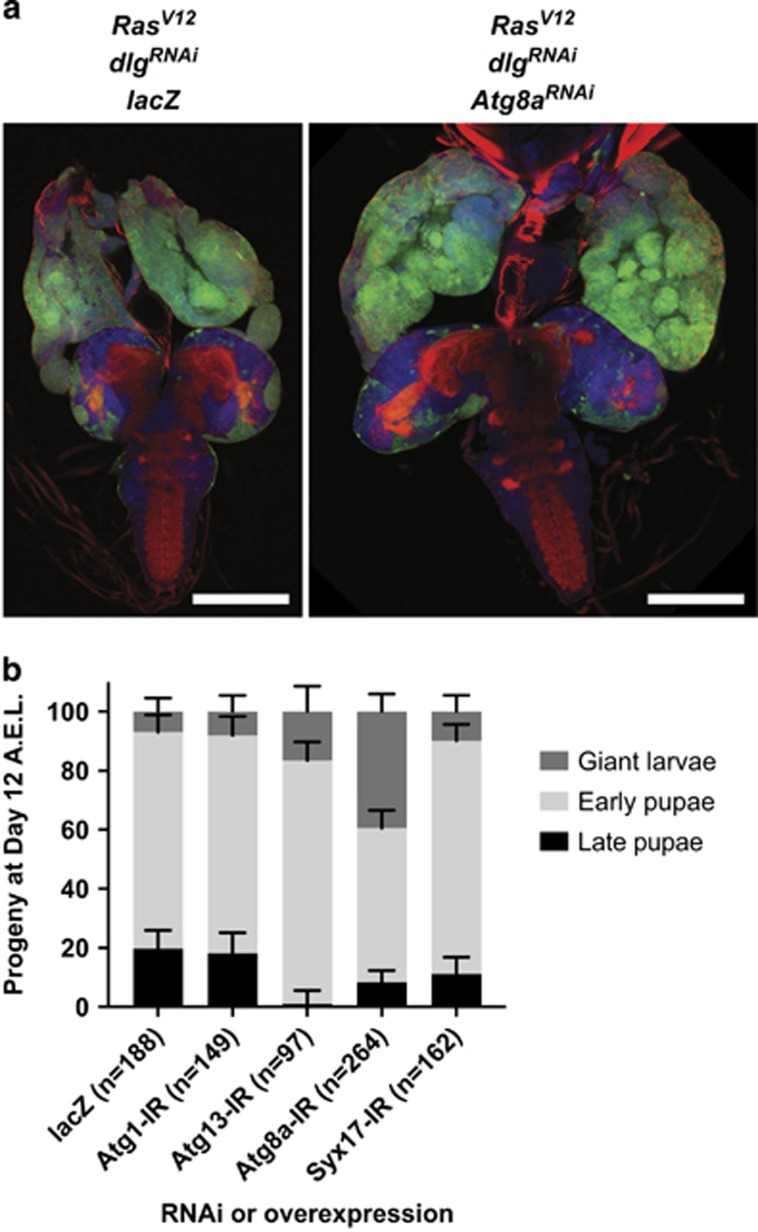
Blocking autophagy enhances polarity-impaired *Ras*^*V12*^-driven epithelial tumours. (**a**) Eye-antennal imaginal discs of *ey-FLP-out Act»Ras*^*V12*^ *dlg*^*RNAi*^ *lacZ* (left) or *ey-FLP-out Act»Ras*^*V12*^ *dlg*^*RNAi*^ *Atg8a*^*RNAi*^ (right) are shown attached to the brain and ventral nerve chord. (**b**) Autophagy inhibition at the induction (*Atg13*^*RNAi*^), elongation (*Atg8a*^*RNAi*^) or fusion to lysosome (*Syx17*^*RNAi*^) step of the pathway enhanced developmental delay in an *ey-FLP-out Act»Ras*^*V12*^ *dlg*^*RNAi*^ cooperative model of tumourigenesis. Inhibition of autophagy at the elongation step, using *Atg8a*^*RNAi*^, accentuated developmental delay, with nearly 40% of animals dying as giant larvae, and only 8% reaching a later pupal stage. Inhibiting autophagy at the autophagosome–lysosome fusion step, using *Syx17*^*RNAi*^, also delayed development of *ey-FLP-out Act»GAL4 Ras*^*V12*^ *dlg*^*RNAi*^ larvae, although not as strongly as *Atg8a*^*RNAi*^, with ~10% dying as giant larvae, and only 11% making it to the later pupal stage. Blocking the initiation step, using *Atg13*^*RNAi*^, markedly enhanced developmental delay, with only 1% of animals reaching the late pupae stage, and 16% dying as giant larvae. However, inhibiting the pathway at the same step, using *Atg1*^*RNAi*^, did not have any substantial effect on *ey-FLP-out Act»GAL4 Ras*^*V12*^ *dlg*^*RNAi*^ development. Error bars=95% confidence intervals.

It has been proposed that the tumour-suppressive role of autophagy in cancer would be limited to early stages of tumour development, while later stages require autophagy to sustain their growth (reviewed in White^21^ and Galluzzi *et al.*^23^). To investigate whether autophagy could also have a tumour-suppressive effect in a more aggressive model of tumourigenesis, we inhibited the pathway in the eye-antennal epithelium in which *Ras*^*V12*^ was expressed together with knockdown of the cell polarity gene, *dlg*. Loss of *dlg* has been previously shown to cooperate with *Ras*^*V12*^ in tumourigenesis,^13, 42, 43^ and human *dlg* orthologs are mutated or downregulated in cancer.^44, 45, 46, 47^ In the *ey-FLP-out Act»GAL4 Ras*^*V12*^ *dlg*^*RNAi*^ model,^43^ mutant tissue (marked by green fluorescent protein (GFP)) overgrows, loses architecture, the eye-antennal discs fuse together and invasion occurs into the brain lobes and ventral nerve chord (Figure 4a—control *lacZ*). As a consequence, normal development is altered: the majority of animals (73%) die early after pupariation, with only 20% of animals reaching a later developmental stage, with adult structures visible through the pupal case, and 7% of animals failing to pupariate and dying as giant larvae (Figure 4b—control *lacZ*). Inhibiting autophagy at the elongation step, using *Atg8a*^*RNAi*^, increased third instar larval eye-antennal disc size (Figure 4a), and accentuated developmental delay, with nearly 40% of animals dying as giant larvae, and only 8% reaching the late pupal stage (Figure 4b). Inhibiting autophagy at the autophagosome–lysosome fusion step, using *Syx17*^*RNAi*^, also delayed the development of *ey-FLP-out Act»GAL4 Ras*^*V12*^ *dlg*^*RNAi*^ larvae, although not as strongly as *Atg8a*^*RNAi*^ (Figure 4b). Blocking the initiation step, using *Atg13*^*RNAi*^, markedly enhanced the developmental delay (Figure 4b). However, inhibiting the pathway at the same step, using *Atg1*^*RNAi*^, did not have any substantial effect on *ey-FLP-out Act»GAL4 Ras*^*V12*^ *dlg*^*RNAi*^ development. A difference in the knockdown efficiency in the eye-antennal disc is plausible; however, we have shown that both RNAis efficiently block the autophagic flux in Ras-activated wing epithelium (Supplementary Figure 4). Thus, specific functions of *Atg1* and *Atg13* are more likely to account for this difference. Indeed, it has recently been shown that Atg1 has autophagy-independent roles in tissue growth.^48^ Altogether, these data show that blocking autophagy at multiple steps of the pathway can also have a tumour-suppressive role in a more advanced Ras-driven tumoural setting.

### Non-cell-autonomous ectopic proliferation, caspase activation and cell death in *Ras^V12^ Atg^RNAi^* mosaic tissues

**Figure 5:**
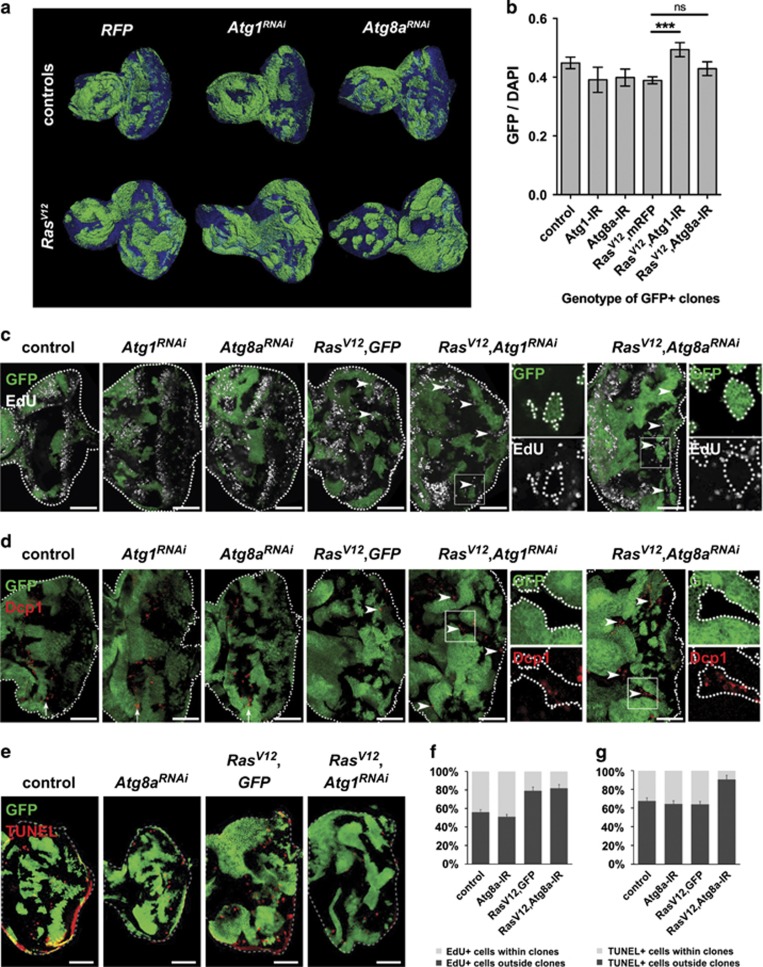
Autophagy inhibition in Ras-activated tissues results in cell- and non-cell-autonomous effects on tissue growth. (**a**) Three-dimensional (3D) reconstruction of *Ras*^*V12*^*Atg*^*RNAi*^ and control eye-antennal mosaic discs from L3 wandering larvae. Mutant clones are GFP+ and tissues were counterstained with DAPI (blue). (**b**) In discs expressing both *Ras*^*V12*^ and *Atg1*^*RNAi*^ in clones, the proportion of GFP tissue was significantly higher than in the control *Ras*^*V12*^ *mRFP* samples (49.4±2.4%, compared to 38.9±1.2%, *P*=0.00028). This trend was also observed in *Ras*^*V12*^ *Atg8a*^*RNAi*^, although the difference was not significantly different (42.9±2.3%, *P*=0.068). (**c**) Non-cell-autonomous proliferation in wild-type tissue (non-GFP) surrounding *Ras*^*V12*^*GFP*, *Ras*^*V12*^ *Atg1*^*RNAi*^ or *Ras*^*V12*^*Atg8a*^*RNAi*^ clones (GFP+), as seen by EdU incorporation. (**d**) Non-cell-autonomous caspase activation in wild-type tissue (non-GFP) surrounding *Ras*^*V12*^*Atg1*^*RNAi*^ or *Ras*^*V12*^*Atg8a*^*RNAi*^ clones (GFP+), as seen by Dcp1 staining. Arrows indicate the apoptotic wave before the MF. (**e**) Apoptosis is confirmed by TUNEL assay around clones expressing *Ras*^*V12*^ and *Atg8a*-RNAi. (**f**') Quantification of data in (**d**). (**g**) Quantification of data in (**e**). Error bars=s.e.m. Statistics: one-way ANOVA with Tukey's multiple correction test. Scale bars: 50 μm.

To further investigate the effect of autophagy inhibition in *Ras*^*V12*^-expressing epithelial tissue in a setting that more closely mimics the development of mammalian tumours, we turned to a clonal system using the mosaic analysis with a repressible cell marker (MARCM) technique,^49^ where *UAS-transgene*-expressing clones are marked by the expression of GFP from a *UAS-GFP* construct. We first examined the representation of GFP-positive tissue in eye-antennal discs of third instar larvae using three-dimensional reconstruction of confocal *z*-sections (Figure 5a). In clones where autophagy was inhibited at the initiation or elongation steps with *Atg1*^*RNAi*^ or *Atg8a*^*RNAi*^, respectively, the ratio of GFP/4',6-diamidino-2-phenylindole (DAPI) volume was not significantly different to the control (Figure 5b). In discs expressing both *Ras*^*V12*^ and *Atg1*^*RNAi*^ in clones, the proportion of GFP tissue was significantly higher relative to control *Ras*^*V12*^ *mRFP* samples (Figure 5b), consistent with the overgrowth we observed in a whole-tissue setting (Figure 2a). This trend was also observed in *Ras*^*V12*^ *Atg8a*^*RNAi*^, although failing to meet statistical significance.

**Figure 6:**
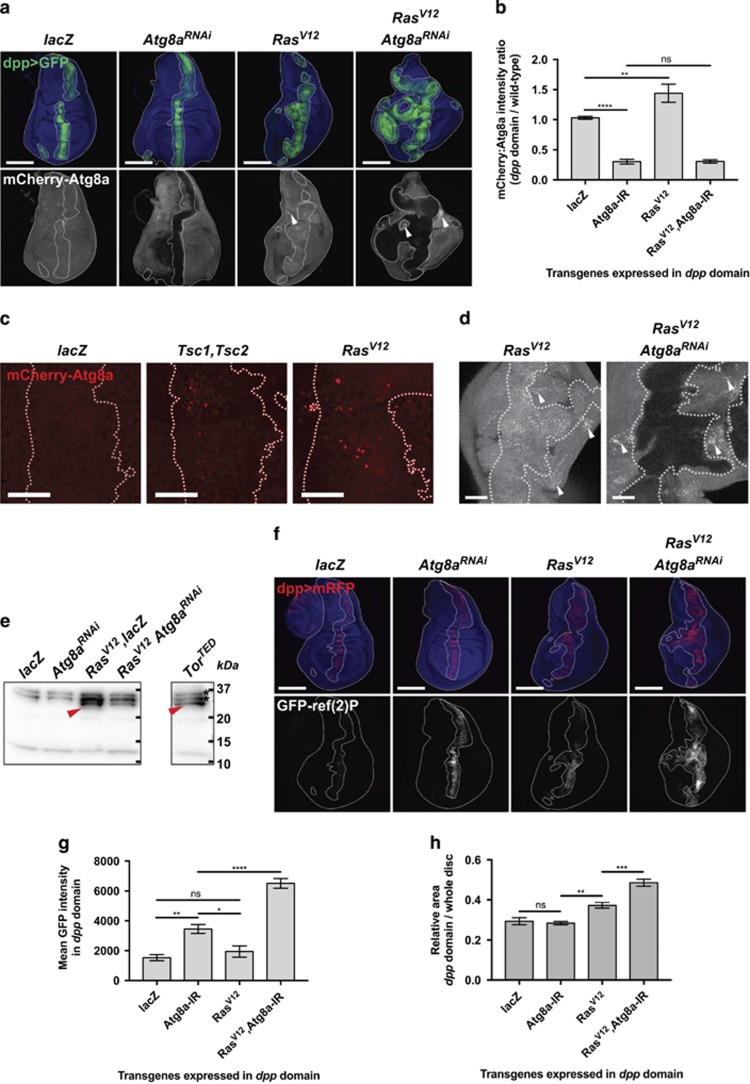
*Ras*^*V12*^ expression induces autophagy. (**a**–**d**) Effect of *Atg8a*^*RNAi*^, *Ras*^*V12*^ and *Ras*^*V12*^ *Atg8a*^*RNAi*^ expressed via the *dpp-GAL4* driver on *pmCherry-Atg8a* expression in L3 wing discs. mCherry-Atg8a levels (**b**) are increased upon Ras activation. (**c**) mCherry-Atg8a punctae are detected in the Dpp domain (dotted lines) upon expression of *Tsc1* and *Tsc2* transgenes (positive control) or *Ras*^*V12*^, while no puncta is detected upon expression of a control *lacZ*. (**d**) Non-cell-autonomous activation of autophagy is also observed in wild-type tissue surrounding *Ras*^*V12*^ and *Ras*^*V12*^ *Atg8a*^*RNAi*^ tissue (arrowheads). (**e**) Monitoring of autophagy flux induction by detection of free mCherry in mCherry-Atg8a tissues. A 27 kDa band corresponding to free mCherry is detected in wing discs expressing *Ras*^*V12*^ and *Ras*^*V12*^ *Atg8a*^*RNAi*^ in the Dpp domain, as well as in the positive control expressing *Tor*^*TED*^. *, unspecified band. (**f**) Effect of *Atg8a*^*RNAi*^, *Ras*^*V12*^ and *Ras*^*V12*^ *Atg8a*^*RNAi*^ expressed via the *dpp-GAL4* driver on GFP-Ref(2)P accumulation in L3 wing discs. *Atg8a* knockdown in the Dpp domain blocks autophagic flux as seen by accumulation of GFP-Ref(2)P aggregates. Slight accumulation of Ref(2)P aggregates is detected upon Ras activation, and blocking autophagic flux in this context leads to massive accumulation of Ref(2)P aggregates in the Dpp domain, quantified in (**g**). (**h**) As in the developing eye epithelium, autophagy inhibition in a Ras-activated background leads to tissue overgrowth, with proportion of GFP+ tissue higher in *Ras*^*V12*^ *Atga-*RNAi compared with Ras-only or *Atg8a*-RNAi-only controls. Scale bars: (**a** and **e**) 100 μm, (**c**) 20 μm and (**d**) 50 μm. Error bars: s.e.m. Statistics: one-way ANOVA with Tukey's multiple correction.

We then examined proliferation using 5-ethynyl-2′-deoxyuridine (EdU) incorporation. Ectopic proliferation posterior to the morphogenetic furrow (MF) was observed in *Ras*^*V12*^ *GFP* clones (Figure 5c, arrowheads), consistent with the role of activated Ras in driving cell proliferation.^50^ Strikingly, in *Ras*^*V12*^ *GFP*, *Ras*^*V12*^ *Atg1*^*RNAi*^ and *Ras*^*V12*^ *Atg8a*^*RNAi*^ mosaic discs we observed that most EdU-positive cells surrounded mutant GFP clones, suggesting that mutant tissue induced non-cell-autonomous proliferation of the neighbouring wild-type tissue (Figure 5c, arrowheads and insets, quantified in Figure 5f). Since the overall mutant-to-wild-type tissue ratio was slightly increased in *Ras*^*V12*^ *Atg*^*RNAi*^ samples compared with *Ras*^*V12*^ *mRFP* (Figures 5a and b), cell proliferation in the wild-type tissue must be overcompensated by apoptosis. Indeed, staining for *Drosophila* death caspase 1 (Dcp1) in mosaic discs revealed that, posterior to the MF, Dcp1 activation occurred preferentially in the wild-type tissue surrounding *Ras*^*V12*^ *Atg*^*RNAi*^ mutant clones, and that much less Dcp1-positive cells were detected in *Ras*^*V12*^ *GFP* mosaic discs (Figure 5d). Indeed, terminal deoxynucleotidyl transferase dUTP nick-end labelling (TUNEL) staining further confirmed that apoptosis was preferentially induced in the vicinity of *Ras*^*V12*^ *Atg8a*^*RNAi*^ tissue, with very little apoptosis detected within clones (Figure 5e, quantified in Figure 5 g). Overall, the reduced apoptosis observed within *Ras*^*V12*^ *Atg8a*^*RNAi*^ clones compared with *Ras*^*V12*^ *GFP* clones could explain the overgrowth of *Ras*^*V12*^ *Atg*^*RNAi*^ tissues in mosaic discs (Figures 6a and b).

High levels of Ras activity has also been shown to induce cell-autonomous differentiation;^51^ however, autophagy inhibition did not alter this effect as seen by staining for the neuronal marker Elav (Supplementary Figure 4). Interestingly, we saw no alterations in proliferation or apoptosis in *Ras*^*V12*^ *Atg*^*RNAi*^ clones. Thus altogether, our data suggest that autophagy inhibition in Ras-activated clones induced non-cell-autonomous proliferation and also caspase activation, and cell death, in the surrounding wild-type tissue, resulting in an overall overgrowth of the mutant tissue.

### Autophagy is induced upon Ras activation

To test whether our observations on inhibiting autophagy in the eye-antennal epithelium was tissue-specific or a more generalised phenomenon for other epithelial tissues, we expressed *Ras*^*V12*^ and *Atg*^*RNAis*^ along the anteroposterior boundary of the wing imaginal disc using the *dpp*^*blk*^*-GAL4* driver (*dpp>*) (Figure 6). In this setting, we examined the effect of the *Ras*^*V12*^ and *Atg*^*RNAi*^ transgenes on autophagic flux *in vivo* by monitoring *Atg8a* expression (using ubiquitous *pmCherry-Atg8a* expressed under endogenous promoter control)^52^ or Ref(2)P levels and aggregation (using *UAS-GFP-ref(*2*)P*).^53^

In controls expressing a *lacZ* construct, faint but homogeneous expression of mCherry-Atg8a (under endogenous promoter control) was detected throughout the disc (Figure 6a, quantified in Figure 6b), while low levels of GFP-Ref(2)P (under *UAS* control) accumulated in the *dpp* expression domain (Dpp strip) (Figure 6f, quantified in Figure 6g). As expected upon *Atg8a*^*RNAi*^ expression, we observed reduced levels of Atg8a (Figures 6a and b—*Atg8a*^*RNAi*^) and accumulation of Ref(2)P aggregates in the Dpp strip (Figures 6f and g—*Atg8a*^*RNAi*^), confirming that in *Atg8a*^*RNAi*^ tissue, autophagy flux is efficiently impaired. In *Ras*^*V12*^-expressing samples, mCherry-Atg8a levels were elevated compared with wild-type surrounding tissue (Figures 6a and b—*Ras*^*V12*^). Higher magnification of the Dpp domain showed mCherry-Atg8a puncta accumulating upon *Ras*^*V12*^ expression, similarly to that observed with Tsc1,Tsc2 overexpression, which inhibits mTORC1 and induces autophagy^54^ (Figure 6c). This finding suggests that basal autophagy is increased upon Ras activation. Interestingly, we also noticed increased Atg8a levels and punctae in the wild-type tissue adjacent to the Dpp domain expressing *Ras*^*V12*^, and similarly in the wild-type tissue adjacent to the Dpp domain expressing *Ras*^*V12*^ *Atg8a*^*RNAi*^ (Figure 6d). These data suggest that basal autophagy is also induced in a non-cell-autonomous manner in the vicinity of *Ras*^*V12*^ and *Ras*^*V12*^ *Atg8a*^*RNAi*^ tissue (Figure 6d, arrowheads; see also Figure 6a). To investigate the functional induction of the autophagy flux, we sought to detect the release of free mCherry that occurs after the mCherry-Atg8a reporter is processed by the lysosome. Overexpression of a *Tor*^*TED*^ construct, a well-known inducer of autophagy flux,^55^ under the *dpp*-*GAL4* driver used in Figures 6a–d leads to the detection of a ~27kDa protein corresponding to the molecular weight of free mCherry (Figure 6e, right lane, arrowhead). Upon expression of *lacZ* or *Atg8a*^*RNAi*^, no such band is detected, suggesting that mCherry is not cleaved from Atg8a and that flux is not occurring. However, upon Ras expression, we were able to detect free mCherry, confirming that Ras induces autophagy flux in developing *Drosophila* epithelium. Moreover, detection of free mCherry in discs expressing a strip of *Ras*^*V12*^ *Atg8a*^*RNAi*^ tissue also reveals the non-cell-autonomous induction of the pathway, as mCherry is knocked down together with Atg8a in the presence of the RNAi in the strip, and only mCherry-Atg8a expressed in the wild-type portion of the disc is detected (Figures 6a, b and d).

**Figure 7:**
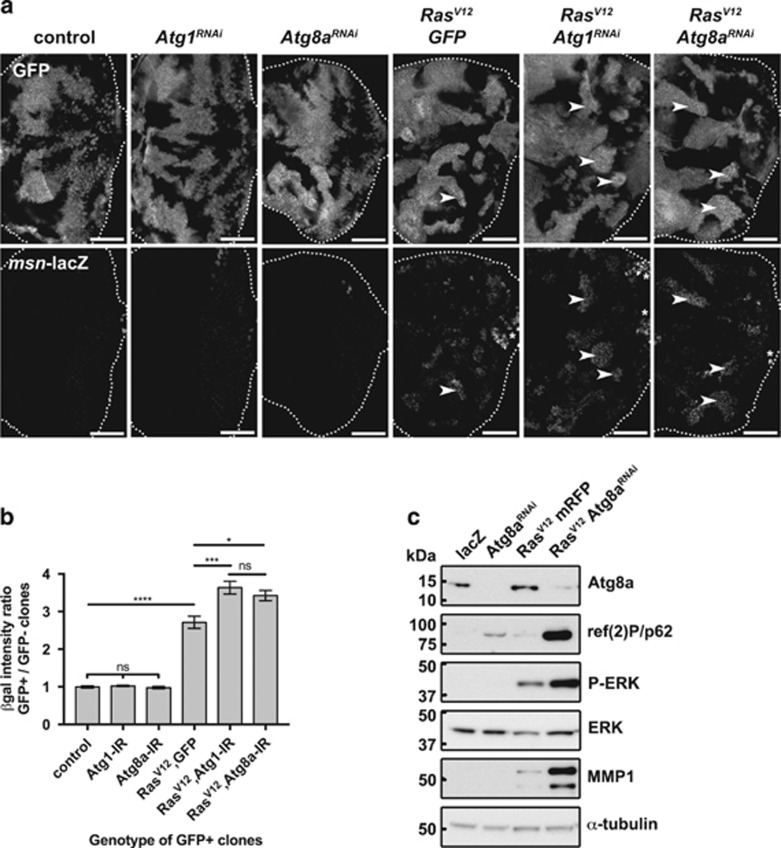
JNK signalling is induced upon autophagy inhibition in *Ras*^*V12*^-expressing tissues. (**a**) JNK pathway activation in *Ras*^*V12*^*Atg1*^*RNAi*^ or *Ras*^*V12*^*Atg8a*^*RNAi*^ clones, as detected by the expression of the *msn*-*lacZ* reporter (arrowheads). Stronger induction of *msn-lacZ* can be seen in *Ras*^*V12*^*Atg*^*RNAi*^ clones compared with *Ras*^*V12*^ *GFP* clones (quantified in (**b**)). Asterisks show endogenous *msn*-*lacZ* expression in glial cells. (**c**) Western blot analyses of Atg8a, Ref(2)P, phospho-ERK, total-ERK and MMP1 relative to α-tubulin in *ey-FLP-out, Act»GAL4 lacZ, Atg8a*^*RNAi*^, *Ras*^*V12*^ or *Ras*^*V12*^*Atg8a*^*RNAi*^ lysates. Ras^V12^ signalling induces autophagy in the eye epithelium, as seen by higher level of Atg8a in *Ras*^*V12*^-expressing tissue compared with control *lacZ* tissue. Ref(2)P accumulation confirms inhibition of the autophagic flux in *Atg8a*^*RNAi*^ and *Ras*^*V12*^*Atg8a*^*RNAi*^ discs. Strong accumulation of Ref(2)P is observed in *Ras*^*V12*^*Atg8a*^*RNAi*^. Ras-MAPK signalling is increased in *Ras*^*V12*^*Atg8a*^*RNAi*^ tissue, compared with *Ras*^*V12*^ alone, as seen by increased phospho-ERK levels. Strong induction of the JNK target MMP1 in *Ras*^*V12*^*Atg*^*RNAi*^ discs reveals synergistic activation of the JNK pathway in *Ras*^*V12*^ tissue upon autophagy inhibition. Scale bars: 50 μm. Statistics (**b**): mean±s.e.m., one-way ANOVA with Tukey's multiple correction.

Increased levels of GFP-Ref(2)P were also observed upon *Ras*^*V12*^ expression (Figures 6f and g—*Ras*^*V12*^). Oncogene-induced upregulation of p62 has been shown to occur consecutive to activation of the Nrf2-dependent unfolded protein response downstream of Myc in *Drosophila*,^56^ as well as upon RAS activation in mammalian systems.^57, 58^ Thus, upregulation of p62 levels is not necessarily the sign of autophagy blockage, but can also reflect upregulation of the pathway from a low baseline (as in developing epithelia). In our case, endogenous p62 upregulation could increase the pool of p62 and explain the difference in clearance of the GFP-p62 fusion protein compared with control (see also Figure 7c). Supporting this hypothesis, blocking autophagic flux in the *Ras*^*V12*^-expressing Dpp strip by expressing *Atg8a-RNAi* led to massive accumulation of Ref(2)P aggregates, much more than upon *Atg8a* knockdown alone (Figures 6f and g—*Ras*^*V12*^ *Atg8a*^*RNAi*^), suggesting that *Ras*^*V12*^ expression sensitises the cells to autophagy impairment.

Importantly, *dpp>Ras*^*V12*^ expression induced overgrowth of the Dpp strip, which was enhanced by *Atg8a* knockdown (Figure 6h). Of note, the wing disc outside of the Dpp strip was distorted in *Ras*^*V12*^ *Atg8a*^*RNAi*^ samples (Figures 6a and f), suggesting that non-cell-autonomous tissue growth effects might also be occurring, as was observed in the eye-antennal disc.

In summary, our data shows that, in developing epithelia, autophagy is induced cell and non-cell autonomously in response to Ras activation. Impairing autophagy (by *Atg8a* knockdown) in a Ras-activated background leads to massive accumulation of Malumbres and Barbacid^2^ P aggregates and promotes non-cell-autonomous autophagy (revealed by Atg8a aggregates). Finally, as in the eye epithelium, autophagy inhibition enhanced growth of the *Ras*^*V12*^-expressing wing epithelium, confirming the tumour-suppressive role of autophagy in Ras-activated *Drosophila* epithelia.

### ERK and JNK signalling are enhanced upon autophagy blockage in Ras-activated tissues

We and others have previously shown that Ras-cooperative overgrowth often depends on the co-activation of the stress-induced JNK branch of the mitogen-activated protein kinase (MAPK) pathway.^35, 36, 37^ To test whether blocking autophagy in a Ras-activated background could induce the JNK pathway, we assessed the levels of the well-known JNK target *misshapen* (*msn*), using a *msn-lacZ* enhancer-trap reporter^59^ in eye-antennal disc clones (Figure 7a). While no expression of the *msn*-*lacZ* reporter was detected in control, *Atg1*^*RNAi*^ or *Atg8a*^*RNAi*^ clones, strong induction of *msn-lacZ* was detected in *Ras*^*V12*^ *Atg1*^*RNAi*^ and *Ras*^*V12*^ *Atg8a*^*RNAi*^ clones, relative to the milder induction that occurred in *Ras*^*V12*^ *GFP* clones (Figure 7a, quantified in Figure 7b).

Cross-talk and feedback mechanisms between autophagy and MAPK signalling have been reported previously in mammals.^60, 61, 62, 63^ To investigate further the activation of the extracellular signal-regulated kinase (ERK) and JNK branches of the MAPK pathway when autophagy is blocked in a Ras-activated background, we looked at protein levels in lysates of *ey-FLP-out*, *Act*»*GAL4* eye-antennal discs (Figure 7c). First, we confirmed our previous observation that basal autophagy is induced upon Ras activation, as seen by increased Atg8a levels (Figure 7c—lane 3, quantified in Supplementary Figure 6). Again, blocking autophagic flux with *Atg8a*^*RNAi*^ in this context led to a strong accumulation of Ref(2)P (Figure 7c—lane 4 and Supplementary Figure 6).

Then, we monitored the levels of phospho-ERK (active) and total-ERK in eye tissue lysates. Phospho-ERK was below detectable levels in control *lacZ* and *Atg8a*^*RNAi*^ samples, and was elevated as expected in *Ras*^*V12*^ *mRFP* samples (Figure 7c—lanes 1–3). Strikingly, strong upregulation of phospho-ERK levels was detected in *Ras*^*V12*^ *Atg8a*^*RNAi*^ samples, suggesting that blocking autophagy increased the flux through the Ras-ERK pathway (Figure 7c—lane 4 and Supplementary Figure 6). These observations are consistent with recent mammalian studies showing that autophagy proteins regulate ERK phosphorylation and that autophagosomes can serve as intracellular scaffolds for the MAPK cascade.^61^

Finally, we monitored the levels of MMP1, a known JNK target. MMP1 was upregulated by *Ras*^*V12*^ relative to the *lacZ* control and *Atg8a*^*RNAi*^ alone (Figure 7c—lane 3), and a synergistic increase in MMP1 levels was observed in *Ras*^*V12*^ *Atg8a*^*RNAi*^ tissue (Figure 7c—lane 4, quantified in Supplementary Figure 6). This observation, along with the high levels of *msn-lacZ* induced in *Ras*^*V12*^ *Atg8a*^*RNAi*^ mosaic discs (Figure 7a), confirms that, upon autophagy inhibition, the JNK pathway is robustly induced in Ras-activated tissues.

### Autophagy inhibition in Ras-activated tissue induces massive accumulation of reactive oxygen species

Autophagy is a key homeostasis mechanism by which cells remove defective organelles and protein aggregates to prevent oxidative stress.^16, 17^ Moreover, it has been documented that the JNK stress response module is activated in response to reactive oxygen species (ROS) accumulation.^64^ Thus, we sought to investigate whether the enhanced overgrowth observed in *Ras*^*V12*^ *Atg*^*RNAi*^ tissues is caused by ROS-induced JNK activation.

**Figure 8:**
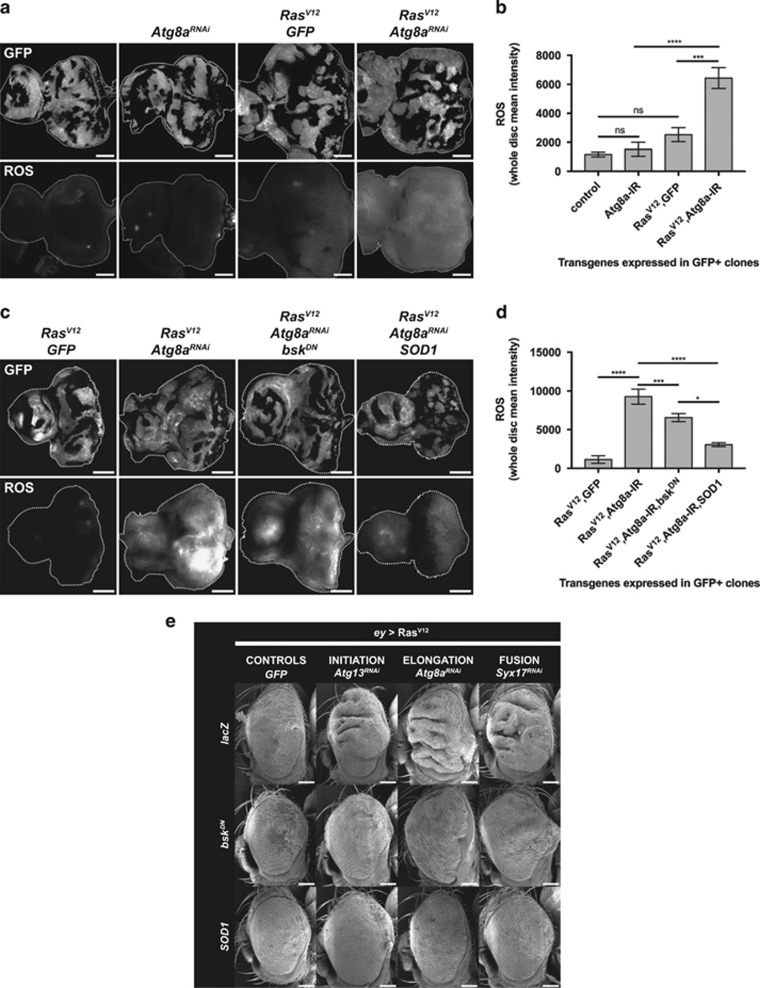
Upon autophagy inhibition, ROS accumulate in Ras-activated tissue and lead to cooperative tissue overgrowth via JNK activation. (**a**) Oxidative stress levels in eye-antennal mosaic discs as detected by CellROX Deep Red staining. Autophagy inhibition using *Atg8a*^*RNAi*^ did not augment ROS levels compared with control discs. Ras activation (*Ras*^*V12*^ *GFP*) slightly increased ROS levels. However, massive accumulation of ROS was detected in *Ras*^*V12*^*Atg8a*^*RNAi*^ mosaic tissue. Interestingly, ROS are not limited to *Ras*^*V12*^*Atg8a*^*RNAi*^ clones, but spread in the whole disc, suggesting a non-cell-autonomous regulation of oxidative stress levels in *Ras*^*V12*^*Atg*^*RNAi*^ mosaic tissue. Scale bar: 50 μm. (**b**) Quantification of ROS levels in whole discs from (**a**). (**c**) ROS levels in *Ras*^*V12*^ *Atg8a*^*RNAi*^ mosaic discs are reduced upon *SOD1* overexpression, but not upon JNK blockage, confirming that JNK activation is downstream of ROS accumulation in *Ras*^*V12*^ *Atg8a*^*RNAi*^ discs. Note that overexpression of *SOD1* rescues both cell-autonomous and non-cell-autonomous accumulation of ROS, and restores the size and shape of *Ras*^*V12*^ *Atg8a*^*RNAi*^ eye discs. (**d**) Quantification of ROS levels in whole discs from (**c**). (**e**) Scanning electron micrographs of adult eyes expressing *Ras*^*V12*^ and *GFP* (first column), *Atg13*^*RNAi*^ (second column), *Atg8a*^*RNAi*^ (third column) or *Syx17*^*RNAi*^ (fourth column), in combination with control *lacZ* (first row), *bsk*^*DN*^ (second row) or *SOD1* (third row). *Ras*^*V12*^*Atg*^*RNAi*^ eye overgrowth is rescued by overexpression of *bsk*^*DN*^ or *SOD1*, showing that autophagy-impaired *Ras*^*V12*^-driven tumourigenesis is due to elevated ROS levels and JNK signalling. Note that overexpression of *SOD1* and *bsk*^*DN*^ also rescues Ras overgrowth alone (first column). Error bars=s.e.m. Statistics: one-way ANOVA with Tukey's multiple correction.

First, we monitored ROS levels upon autophagy inhibition of Ras-activated tissues by performing CellROX Deep Red staining on mosaic eye-antennal discs (Figure 8a). Expression of *lacZ* control or *Atg8a*-RNAi only did not lead to ROS accumulation, and expression of *Ras*^*V12*^ alone only slightly increased oxidative stress levels (Figure 8a, quantified in Figure 8b). However, when autophagy was blocked in *Ras*^*V12*^-expressing clones, we observed massive accumulation of ROS (Figures 8a and b—*Ras*^*V12*^ *Atg8a*^*RNAi*^). Surprisingly, the accumulation of CellROX staining was not entirely cell autonomous, suggesting that either ROS diffused from mutant clones into the microenvironment or that mutant tissue induced ROS production in neighbouring wild-type tissue (Figure 8a and Supplementary Figure 7a—*Ras*^*V12*^ *Atg8a*^*RNAi*^).

Blocking JNK by overexpressing a dominant-negative form of the JNK orthologue basket (*bsk*^*DN*^) in *Ras*^*V12*^ *Atg8a*^*RNAi*^ clones restored the architecture of the tissue but only partially reduced ROS levels (Figure 8c, quantified in Figure 8d), confirming that oxidative stress cues are mostly upstream of JNK activation. However, reducing oxidative stress by overexpressing *superoxide dismutase 1* (*SOD1*) strikingly rescued disc overgrowth, restored tissue architecture and markedly reduced ROS levels (Figures 8c and d).

### Accumulation of ROS and JNK activation are responsible for the cooperation of Atg knockdown and Ras^V12^ in tissue overgrowth

To test whether JNK activation was causal to the overgrowth seen in *Ras*^*V12*^ *Atg*^*RNAi*^ adult *Drosophila* eye, we overexpressed *bsk*^*DN*^ in Ras-activated tissues in which autophagy was blocked at the induction (*Atg13*^*RNAi*^), elongation (*Atg8a*^*RNAi*^) or fusion to the lysosome (*Syx17*^*RNAi*^) steps (Figure 8c). *bsk*^*DN*^ rescued the overgrowth of *Ras*^*V12*^ *Atg13*^*RNAi*^, *Ras*^*V12*^ *Atg8a*^*RNAi*^ and *Ras*^*V12*^ *Syx17*^*RNAi*^ eye epithelial tissue (Figure 8c, quantified in Supplementary Figure 7b). Strikingly, reducing ROS levels, by overexpressing *SOD1*, strongly rescued the overgrowth of *Ras*^*V12*^ *Atg13*^*RNAi*^, *Ras*^*V12*^ *Atg8a*^*RNAi*^ and *Ras*^*V12*^ *Syx17*^*RNAi*^ eye epithelial tissue to a greater extent than blocking JNK (Figure 8c and Supplementary Figure 7b).

Altogether, these data show that, in Ras-activated tissues, blocking autophagy at several steps of the pathway leads to marked accumulation of ROS and JNK activation, and that these events are responsible for cooperative overgrowth with activated Ras.

## Discussion

**Figure 9:**
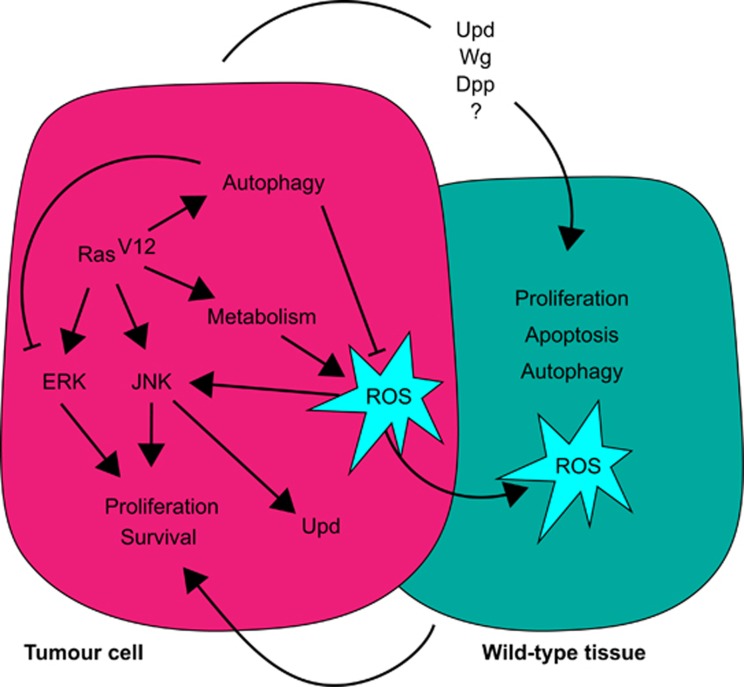
Model of cooperation between Ras and the autophagy pathway. Autophagy induction upon Ras-activation limits the accumulation of reactive oxygen species (ROS) and the activation of the ERK pathway. When autophagy is blocked in *Ras*^*V12*^-expressing cells, ERK is further activated, ROS accumulate and activate the JNK pathway, resulting in cooperative overgrowth of Ras^*V12*^ tissues by cell-autonomous and non-cell autonomous mechanisms. ROS are detected in neighbouring wild-type tissue, and non-cell autonomous activation of autophagy, proliferation, caspase activation and cell death is observed, which might feed back to promote the proliferation and survival of the *Ras*^*V12*^ cells.

Autophagy’s involvement in cancer initiation and progression has drawn much attention over the past few years. Autophagy can be oncogenic as well as tumour suppressive depending on context and tumour stage.^21, 22, 23^ In this study, following up on a genome-wide screen for Ras-cooperative tumour suppressors that identified members of the autophagy pathway, we investigate the mechanisms by which autophagy contains the growth of Ras-activated tissues. We show that, in *Ras*^*V12*^-expressing epithelial tissues, inhibition of autophagic flux at different steps of the pathway results in the accumulation of ROS and activation of the JNK stress response module, leading to overgrowth of mutant tissue (Figure 9).

In our model, functional autophagy is required to restrain the growth of Ras-activated epithelial tissue growth in a clonal or whole-tissue setting, as well as in combination with impaired cell polarity. This is in contrast with a previous report where autophagy depletion was reported to reduce the growth of Ras-activated *Drosophila* tissues, alone or in combination with *scrib* loss of function.^65^ One factor that might explain the difference between the two studies is the different promoters used to drive the *Ras* oncogene, and the overall number of *UAS* constructs in the system, raising questions about levels of Ras expression in our study and theirs. With Ras levels possibly determining the differential output of autophagy,^26^ this observation could be instrumental for further investigating the interplay between the two pathways.

In our analysis, we observed cross-talk between the autophagy and Ras signalling pathways: activating Ras-induced autophagy, and blocking autophagy in this context further increased ERK activity (Figures 7 and 9). By its activation of the PI3K/AKT/mTOR pathway, Ras is predicted to inhibit autophagy.^66^ However, there is increasing evidence that Ras positively regulates autophagy, and higher levels of autophagy are observed in Ras-expressing tumour cells.^24, 58, 67, 68^ Furthermore, the proto-oncogene Myc, a downstream activator of Ras signalling, also induces autophagy and tissue growth in the *Drosophila* wing disc.^56^ We show here that positive regulation of autophagy by Ras is conserved in *Drosophila*. Conversely, autophagy regulates the Ras signalling pathway. For instance, it has been suggested that autophagosomes can act as intracellular scaffolds for players of the MAPK cascade, and that members of the LC3 family, including GABARAP, colocalise with phospho-ERK at the surface of autophagosomes.^61^ This direct regulation of intracellular signalling by autophagy is not unique: it has been shown in a lung cancer cell line that autophagy specifically promotes RHOA degradation and therefore downregulates RHOA signalling.^69^ Thus, these observations raise the possibility that some of the effects of autophagy inhibition on Ras-driven overgrowth lie in direct regulation of intracellular signalling by the autophagic machinery.

An important insight from our study is our demonstration that ROS accumulation and the induction of the JNK stress response pathway potentiates the tissue growth induced by activated Ras. ROS accumulation upon autophagy inhibition in Ras-driven cancer is usually detrimental to tumour growth, as it leads to DNA damage, chromosomal instability, and apoptosis.^70, 71^ In line with these observations, increasing ROS levels has been used as a way to trigger apoptosis in pancreatic cancer cells,^71^ and strong induction of ROS and JNK activity also triggers apoptosis in *Drosophila* models of chromosomal instability.^72^ In our model however, the apoptosis-inducing effects of ROS and JNK are prevented by the cell survival properties of the Ras oncogene, thereby repurposing the action of JNK towards blocking differentiation, pupariation and promoting an invasive phenotype.^9, 11, 73^

The interplay between autophagy, ROS and JNK has been evidenced in several mammalian studies: in response to oncogenic RAS, JNK is critical for *Atg5* and *Atg7* upregulation and autophagy induction,^62 63^ and treatment with antioxidants is sufficient to abolish RAS-induced JNK activation.^62^ We show that lowering oxidative stress levels by overexpressing *SOD1* is able to rescue overgrowth of autophagy-impaired, Ras-activated tissues, to a greater extent than blocking JNK. This suggests that there might be JNK-independent pathways that are activated by ROS that contribute to tissue overgrowth. Indeed, other studies have shown that the p38 MAPK stress response pathway is also activated upon oxidative stress,^74^ and ROS-induced JNK and p38 signalling synergise to promote tissue regeneration in *Drosophila*.^75^

One issue that we are yet to resolve is the origin of ROS upon autophagy impairment in *Drosophila Ras*^*V12*^-expressing tissues. Interestingly, in mammalian cells, p62 accumulation promotes tumourigenesis *via* upregulation of oxidative stress, and removal of p62 by autophagy is sufficient to reduce tumour burden.^76^ Thus, the strong accumulation of Ref(2)P/p62 we see upon autophagy inhibition in the Ras-activated background raises the possibility that a similar p62-dependent mechanism might be at work in our *Drosophila* model. On the other hand, oncogenic Ras-driven transformation is associated with upregulation of metabolic pathways and increased ROS production due to the accumulation of dysfunctional mitochondria,^26, 77^ a situation that would be exacerbated by blocking autophagy. Supporting this model, ROS-induced JNK activation and non-cell-autonomous induction of proliferation has been documented in mosaic *Drosophila* tissues expressing oncogenic Ras and inactivating mutations in mitochondrial genes.^14^

In our system, we also observed non-cell-autonomous effects on wild-type tissues surrounding *Ras*^*V12*^-autophagy-impaired mutant cells, including the induction of autophagy, increased active caspase, cell death and cell proliferation. Since blocking autophagy in *Ras*^*V12*^-expressing cells markedly augments ROS levels, ROS could be leading to these effects on the surrounding wild-type tissue by diffusion (Figure 9). In parallel, the upregulation of ROS and JNK could lead to the induction of paracrine factors, such as Dpp (TGFβ), Wingless (WNT) or Upd (IL6), that affect the surrounding tissue^75, 78^ (Figure 9). Similar non-cell-autonomous effects of autophagy impairment have been observed in mammalian systems, where blocking autophagy results in an inflammatory microenvironment.^79, 80^ It is possible that this ROS-mediated inflammatory signalling also participates in the non-cell-autonomous growth we observe in our model. In line with this hypothesis, a new study has highlighted the importance of extracellular ROS in inducing apoptosis-induced proliferation *via* the recruitment of macrophage-like cells (haemocytes) to regenerating *Drosophila* tissues,^81^ raising the possibility that similar cross-talk between immune cells and the epithelium drives overgrowth of Ras-activated tissues upon autophagy inhibition.

During the course of this work, a study by Katheder *et al.*^82^ highlighted the non-cell-autonomous contribution of autophagy to *Ras*^*V12*^, *scrib*^*−/−*^ tumour growth. In mosaic tissues, autophagy is induced in the wild-type cells surrounding *Ras*^*V12*^ clones, and is further enhanced in the neighbouring cells of *Ras*^*V12*^*, scrib*^*−/−*^ clones to sustain their proliferation, suggesting that non-cell-autonomous regulation of autophagy and proliferative/apoptotic mechanisms in tissues expressing a constitutively active form of the Ras oncogene, or in Ras-cooperative tumourigenesis settings, is a general mechanism.

In summary, our *Drosophila* model of Ras-driven cooperative tumourigenesis has elucidated that ROS and JNK activation are critical for synergistic tissue growth. Given the effect of autophagy inhibition on ROS levels upon Ras activation, and the active development of autophagy blockers as cancer therapeutic agents, it is now crucial to identify situations in which inhibiting this pathway could lead to enhanced tumourigenesis rather than block progression of tumours. Indeed, recent studies are revealing that the protective effect of autophagy on ROS levels is sufficient to suppress tumour progression and metastasis.^83^ Therefore, understanding the complex interplay between Ras, ROS and autophagy is crucial to enable the development of new therapeutic protocols targeting the autophagy pathway in cancer patients. Our data indicate that *Drosophila* will be a powerful model by which to decipher this interplay.

## Materials and methods

### Fly genetics

*Drosophila* stocks used:

- *y,w,ey-FLP1;act>CD2>GAL4,UAS-GFP* (*eyeless-FLP-out, actin-GAL4* system)
- *ey-GAL4,UAS-Ras* ^*V12*^ */CyO*
- *y,w,ey-FLP1,UAS-mCD8-GFP;tub-GAL4,FRT82B,tub-GAL80/TM6B,Tb* (MARCM3R)
- *ey-FLP1;UAS-dlg* ^*RNAi*^ *,UAS-Ras* ^*V12*^ *;act>CD2>GAL4,UAS-GFP*
- *ey-GAL4,UAS-Ras* ^*V12*^ */CyO,tub-GAL80;UAS-SOD1*
- *ey-GAL4,UAS-Ras* ^*V12*^ */CyO,tub-GAL80;UAS-bsk* ^*DN*^
- *ey-GAL4,UAS-Ras* ^*V12*^ */CyO,tub-GAL80;UAS-GFP*
- *pmCherry-Atg8a;dpp* ^*blk*^ *-GAL4,UAS-GFP/SM6a-TM6B,Tb*
- *UAS-GFP-* *ref(2* **)*P;dpp* ^*blk*^ *-GAL4,UAS-His2Av:mRFP/SM6a-TM6B,Tb*
- *UAS-Ras* ^*V12*^ *,UAS-Atg8a-IR* ^*KK109654*^
- *UAS-Ras* ^*V12*^ *,UAS-Atg1-IR* ^*GD16133*^
- *UAS-Ras* ^*V12*^ *;UAS-Atg9-IR* ^*GD10045*^
- *UAS-Ras* ^*V12*^ *,UAS-GFP*
- *UAS-Ras* ^*V12*^ *,UAS-myr-mRFP*
- *UAS-SOD1* (BL33606, kindly provided by S Gregory)
- *UAS-bsk* ^*DN*^
- *UAS-lacZ*
- *msn-lacZ,FRT82B (P(PZ)msn* ^*06948*^)
- *pmCherry-Atg8a* (a gift from D Denton)
- *UAS-GFP-* *ref(2* **)*P* (a gift from T Neufeld)

In all stocks, *UAS-Ras*^*V12*^ refers to *UAS-dRas85D*^*V12*^ on the second chromosome.

### RNAi lines

See Supplementary Table 1.

All stocks and experimental crosses were performed on molasses and yeast based food, in 25 °C or 29 °C incubators. In all experiments involving dissection of larvae, flies were left laying for 8 to 16 h, and wandering L3 larvae were collected at day 5 AEL) (when raised at 25 °C), or day 4 AEL (when raised at 29 °C).

### Pupariation assays

In *ey-FLP-out, Act»GAL4* pupariation assays, late second to early third instar larvae of correct genotypes were collected at day 4 AEL, counted and transferred (50 individuals) into fresh vials of food. Pupae were counted every 24 h after sorting, from day 5 AEL to day 9 AEL. Hatched adults were counted from day 10 AEL to day 14 AEL. Results were plotted in GraphPad Prism (GraphPad Software, La Jolla CA, USA) with 95% confidence intervals. Pupariation assays were performed two times.

### Western blot analysis

Twenty to 40 pairs of eye/antennal or wing imaginal discs were dissected in cold phosphate-buffered saline (PBS) and homogenised in RIPA. Six micrograms of proteins were separated by electrophoresis on a 4–12% Bis-Tris gel (Thermo Fisher, Waltham, MA, USA) and blotted onto a PVDF membrane. Blocking and antibody incubations were performed in TBS+0.1% Tween-20 (TBST)+5% skimmed milk. Three biological repeats were performed for the western blot experiment in Figure 7c (quantified in Supplementary Figure 6). Western blot in Figure 6e was performed once.

### Immunofluorescence analysis

Wandering third instar larvae were dissected in cold PBS and inverted cuticles were fixed in 4% paraformaldehyde. Blocking and antibody incubations were performed in PBS+0.3% Triton X-100+2% goat serum. Primary incubations were performed overnight at 4 °C, protected from light on a rotating wheel. Secondary incubations were performed for 2 h at room temperature (RT), protected from light on a nutator platform. All washes were performed in PBS+0.3% Triton X-100. Samples were then immersed in 80% glycerol: 20% PBS, and mounted

### Antibodies

Primary antibodies used were as follows: rabbit polyclonal anti-ref(2)p (1:10 000 (WB), 1:2000 (IF); a gift from G Juhász), rabbit polyclonal anti-Atg8a (1:5000 (WB); a gift from T Neufeld), mouse anti-MMP1 (1:100 (WB); Developmental Studies Hybridoma Bank, University of Iowa, IA, USA), mouse monoclonal anti-Elav (1:200; Developmental Studies Hybridoma Bank), rabbit polyclonal anti-β-galactosidase (1:200; Molecular Probes, Eugene, OR, USA), rabbit polyclonal anti-Dcp1 (1:200; Cell Signaling, Danvers, MA, USA; a kind gift of L Cheng), mouse monoclonal anti-activated ERK1/2 (phospho-ERK 1:5000; Sigma, St Louis, MO, USA), rabbit polyclonal anti-ERK1/2 (total-ERK 1:5000; Cell Signaling), mouse monoclonal anti-α-tubulin (1:10 000; Sigma), rabbit polyclonal anti-mCherry (1:6000; Kerafast, Boston, MA, USA; EMU109). The secondary antibodies used were as follows: goat anti-rabbit:HRP (1:5000), goat anti-mouse:HRP (1:15 000), goat anti-rabbit:Alexa-647(1:500; Molecular Probes) and goat anti-mouse:Alexa-568 (1:500; Molecular Probes). Dcp1 staining experiments were conducted once, mCherry-Atg8a detection experiments was performed more than five times and GFP-p62 detection experiments were performed three times.

### EdU incorporation assay

EdU incorporation assay (Life Technologies, Thermo Fisher) was performed as follows. Inverted L3 larvae were incubated for 20 min in PBS+50 nm EdU (check) at RT. Tissue was fixed in 4% paraformaldehyde for 20 min at RT, washed 3x times with PBS+0.5% Triton X-100, incubated with antibody against GFP and washed and incubated with secondary antibodies, before EdU revelation using Click-iT protocol (Life Technologies, Thermo Fisher). EdU assays were carried out two times.

### TUNEL assay

TUNEL assay was performed using the Click-iT Plus TUNEL assay (Molecular Probes; cat. no. C10619) following guidelines for cells with the following differences: permeabilization of tissues was performed overnight in PBS+0.3% Triton X-100, TdT reaction was performed by incubating overnight followed by 1.5 h at 37 °C, and incubation with Click-iT reagent was performed for 45 min at 37 °C. TUNEL experiments were performed two times.

### Detection of ROS

Detection of ROS was performed in imaginal discs using CellROX Deep Red (Life Technologies, Thermo Fisher), following the protocol published previously.^72^ Briefly, eye-antennal imaginal discs were dissected in Shields and Sang M3 medium at RT, and transferred into a 1.5 ml Eppendorf tube. Dissection medium was removed and replaced by a freshly made solution of 5 μm CellROX Deep Red in M3 medium at RT. Samples were incubated for 8 min on a nutator, protected from light. CellROX was removed and samples were washed quickly two times in RT PBS, and briefly fixed with 4% paraformaldehyde for 4 min on a nutator, protected from light. Paraformaldehyde was removed and samples were washed quickly two times in RT PBS. Discs were immersed in 80% glycerol in PBS, mounted on SuperFrost microscope slides with coverslips and imaged within 30 min of mounting. For detection of CellROX, acquisition parameters were set up on *Ras*^*V12*^ *Atg8a*^*RNAi*^ samples, as they accumulated the most dye, and the same parameters were used on to image other genotypes. ROS assays were carried out three times.

### Confocal microscopy

Confocal images were taken on a Nikon C2 confocal microscope (Nikon, Minato, Tokyo, Japan) or Zeiss LSM 780 Laser Scanning Microscope (Zeiss, Oberkochen, Germany).

### Quantification of immunofluorescence data

For quantification of GFP clones in Figure 4, image stacks were imported into Imaris 7.4 (BIOT). GFP and DAPI volumes were calculated for at least eight discs of each genotype, using the same parameters across samples and genotypes. The ratio of GFP/DAPI was calculated and averaged within each genotype and statistical significance was assessed by one-way analysis of variance (ANOVA) with multiple comparisons in GraphPad Prism (GraphPad Software), which was also used to generate graphical results. To control for dosage effects of the *UAS*/GAL4 system, we used a *UAS-Ras*^*V12*^*,UAS-mRFP* stock as a control for *UAS-Ras*^*V12*^*,UAS-Atg1*^*RNAi*^ and *UAS-Ras*^*V12*^*,UAS-Atg8a*^*RNAi*^ lines. This allowed us to directly compare GFP levels between *Ras*^*V12*^*,mRFP*, *Ras*^*V12*^*,Atg1*^*RNAi*^ and *Ras*^*V12*^*,Atg8a*^*RNAi*^ samples. Summary of *UAS* constructs in MARCM crosses: control FRT=1x*UAS* (*UAS-CD8-GFP*); *Atg1*^*RNAi*^ and *Atg8a*^*RNAi*^=2x*UAS* (*UAS-CD8-GFP* and *UAS-RNAi*); *Ras*^*V12*^,*mRFP*, *Ras*^*V12*^,*Atg1*^*RNAi*^, *Ras*^*V12*^,*Atg8a*^*RNAi*^=3x*UAS* (*UAS-CD8-GFP, UAS-Ras*^*V12*^ *and UAS-mRFP or UAS-RNAi*).

For quantification of EdU and TUNEL, positive cells anterior to the MF were counted in at least four eye-antennal discs.

For quantification of βgal and Elav staining (Figure 7a and Supplementary Figure 5), fluorescence intensity was measured in Photoshop in a 30px diameter area within a GFP clone, and in a 30px area in the wild-type tissue neighbouring the measured clone. Three to six individual discs, and at least six clones per disc, were measured.

For mCherry-Atg8a quantification (Figure 6b), at least three discs of each genotype were imaged and maximum intensity projections were generated. In Photoshop (Adobe Systems Inc., San Jose, CA, USA), a mask was applied over the GFP channel to highlight the region of the *dpp*^*blk*^*-GAL4* driver expression (Dpp domain). mCherry intensity was measured in an area of 30x30 px^2^ within the Dpp domain or within the wild-type domain of the same disc, measurements were exported and intensity ratios were (Dpp/wt) were calculated.

For GPF-ref(2)P quantification at x20 (Figure 6e), mean intensity was measured in single slices throughout the Dpp domain. For quantification of punctae (Supplementary Figures 4b and c), single images were acquired with the x40 objective and a 2x zoom in the middle of the wing pouch. Images were processed in Image J (Bethesda, MD, USA) as follows: first, same threshold was applied on the GFP channel to generate binary images, then a watershed filter was used to separate juxtaposed punctae, and finally, particles were counted in a 80 × 80 px^2^ box within the Dpp domain using the analyse particles plugin. At least eight independent discs were measured per RNAi line.

All measurements were exported and plotted into GraphPad Prism (GraphPad Software), and statistical significance was calculated by one-way ANOVA with multiple comparisons with Tukey's multiple correction test. All statistical tests were two-sided, and the variance is presented as s.e.m.

### Electron microscopy

Adult flies were progressively dehydrated in concentrations of ethanol ranging from 25 to 100% over the course of 4 days on a nutator. Flies were then desiccated by critical point drying in a Leica critical point dryer (Wetzlar, Germany), mounted on steel stubs and coated with 20 nm of gold particles. Representative images of each phenotype were taken on a JEOL JCM-6000 NeoScope scanning electron microscope (Akishima, Tokyo, Japan) at x80 and x300 magnifications. Images were cropped and aligned in Adobe Photoshop.

### Assessment of adult eye overgrowth

*ey-GAL4, UAS-Ras*^*V12*^*/CyO* (*ey>Ras*^*V12*^) female flies were crossed to *UAS-Atg*^*RNAi*^ males or *UAS-lacZ* males for control. Crosses were raised at 29 °C for 11 days before scoring. Enhancement of Ras-driven overgrowth was assessed by eye in at least 20 F1 *ey-GAL4, UAS-Ras*^*V12*^*/UAS-Atg*^*RNAi*^ females by comparing with *ey-GAL4, UAS-Ras*^*V12*^*/UAS-lacZ* females.

### Human data analysis

Human orthologues of *Drosophila* autophagy genes (*Atg8a*, *Atg9*, *Atg7*, *Syx17*, *Vamp7*, *Snap29*, *nSyb*) were defined from the DIOPT v.5.3^84^ reported at FlyBase. We retrieved Cancer Genome Atlas patient samples of RNAseq, KRAS mutation profiles and overall survival data (version 2016_01_28) from the BROAD GDAC database (https://gdac.broadinstitute.org/) for PAAD. The mutational profiles (variant calls) were based on the MutSig algorithm^85^ for PAAD (TP.MutSigNozzleReport2.0.Level_4.2016012800).

For the survival analysis we considered only patient samples that were present in the MutSig report ‘TP.final_analysis_set.maf’ and had clinical overall survival information (last day follow up and status). We retrieved normalised illumina hiseq rnaseqv2 RSEM Level 3 data, the Clinical Level_1 for PAAD. For the analysis only primary solid tumour samples were considered.

For PAAD 132/177 (74%) RNAseq patient samples with KRAS mutations were available. We categorised the patient samples in ‘G12’, ‘other’ for samples with KRAS mutations assigned and ‘none’ for the remaining RNAseq samples.

For the survival analysis based on individual autophagy genes, we split patient samples for each gene into a low expression and a high expression group based on multiple cutoff of gene expression percentiles ranging from 0.2 to 0.8. For the best split cutoff that was estimated based on the lowest *P*-value of a log-rank test, we tested for the enrichment and for the underrepresentation of *KRAS-G12* mutations in the low group compared with the high group using a one-sided Fisher’s exact test. For the enrichment and underrepresentation test for *G12* mutations, the category ‘none’ and ‘other’ were aggregated.

For the survival analysis that considered multiple autophagy genes jointly, we performed a log-rank test between two patient groups that were defined based on a k-means clustering. The patient cohort with a larger average RNAseq expression was denoted as ‘high’ and the patient cohort with lower average expression as ‘low’. The k-means clustering procedure was repeated 100 times on all samples. The patient samples were designated either to the low or high expression cohort based on their most frequently assigned group. For the clustering procedure, we log_e(1+*x*) transformed the normalised RSEM gene RNAseq samples and scaled the data (*z*-transformed and centred) for each gene. For the heatmap visualisations, we ranged the data to values between 0 and 1 by a normal probability distribution function.

The processing and analysis of the data was performed in R. For the survival analysis, we used the survival R package^84^ and survplot function (http://www.cbs.dtu.dk/~eklund/survplot/). The heatmap visualisations were performed with the Complex Heatmap Bioconductor package.

## Figures and Tables

**Table 1:** Autophagy-related genes in *Drosophila* and their corresponding human orthologues

*Autophagy step*	*Drosophila gene*	*CG no.*	*Human orthologue*	*Molecular function*
*Induction*	Atg1	CG10967	ULK1, ULK2	Kinase
	Atg101	CG7053	ATG101	Protein binding
	Atg13	CG7331	ATG13	Protein kinase binding
	Atg17/FIP200	CG1347	RB1CC1	Protein kinase binding
				
*Nucleation*	Atg6	CG5429	BECN1	Protein binding
	Atg14	CG11877	ATG14	Protein binding
	Vps15/ird1	CG9746	PIK3R4	Kinase
	Vps34/Pi3K59F	CG5373	PIK3C3	Kinase
	Atg18a	CG7986	WIPI2	PIP2 binding
	***Atg9***^a^	***CG3615***	***ATG9A, ATG9B***	***Protein binding***
	Atg2	CG1241	ATG2A, ATG2B	Protein binding
				
*Elongation*	Atg3/Aut1	CG6877	ATG3	Ubiquitin-like ligase
	Atg4a	CG4428	ATG4A, ATG4B	Cysteine-type endopeptidase
	Atg4b	CG6194	ATG4C	Cysteine-type endopeptidase
	***Atg7***^a^	***CG5489***	***ATG7***	***Ubiquitin-activating enzyme***
	***Atg8a***^a^	***CG32672***	***GABARAP***	***Ubiquitin-like***
	Atg8b	CG12334	MAP1LC3C, MAP1LC3B2	Ubiquitin-like modifying enzyme
				
*Completion*	Atg10	CG12821	ATG10	Ubiquitin-like ligase
	Atg12	CG10861	ATG12	Ubiquitin-like
	Atg5	CG1643	ATG5	Ubiquitin-like ligase
	Atg16	CG31033	ATG16L1, ATG16L2	Ubiquitin-like ligase
				
*Fusion to the lysosome*	***Syx17***^a^	***CG7452***	***STX17***	***SNAP/SNARE protein binding***
	***Vamp7***^a^	***CG1599***	***VAMP7, VAMP8***	***SNARE protein binding***
	***Snap29/usnp***^a^	***CG11173***	***SNAP29***	***SNAP receptor***
	***nSyb***^a^	***CG17248***	***VAMP1, VAMP2, VAMP3***	***SNARE protein binding***

a Bold Italics indicate genes identified in a screen for Ras-cooperative tumour suppressor genes.

## Supplementary Material

Supplementary Figures and Tables

Supplementary Data set 1

Supplementary Data set 2
