# Supplementary material for: Autophagy suppresses Ras-driven epithelial tumourigenesis by limiting the accumulation of reactive oxygen species
Source: Oncogene. 2017 Jun 5;36(40):5576–92. doi: 10.1038/onc.2017.175 (PMC5633656; doi:10.1038/onc.2017.175)
Supplement: Supplementary Information [file onc2017175x1.pdf]

SUPPLEMENTARY INFORMATION

Manent *et al.*, "Autophagy suppresses Ras-driven epithelial tumourigenesis by limiting the accumulation of reactive oxygen species"

Supplementary Figure 1: Blocking autophagy cooperates with oncogenic Ras in tumourigenesis.

Supplementary Figure 2: Independent RNAi lines targeting the autophagy pathway enhance Ras-driven overgrowth of the adult *Drosophila* eye.

Supplementary Figure 3: Efficient blockage of autophagic flux in Ras-activated epithelial tissue by RNA interference as seen by monitoring of a GFP-ref(2)P reporter.

Supplementary Figure 4: Classification of pancreatic ductal adeno carcinomas (PAAD) on the mean expression of autophagy-related genes is predictive of poorer outcome and correlates with higher percentage of KRAS G12 activating mutations.

Supplementary Figure 5: Blocking autophagy does not alter Elav expression in *Ras^{V12}*-expressing clones.

Supplementary Figure 6: Quantification of Western blot analysis in the *ey-FLP-out, act>>GAL4* system at 29°C (Figure 7c).

Supplementary Figure 7: Detection of ROS is not limited to clonal tissue in *Ras^{V12} $Atg8a^{RNAi}$* mosaic discs and *bsk^{DN}* or *SOD1* overexpression rescues head overgrowth of *Ras^{V12} Atg^{RNAi}* flies.

Supplementary Table 1: RNAi lines and their *ey-GAL4*, *UAS-Ras^{V12}* enhancement score at 29°C.

Supplementary Table 2: RNAseq data analysis of human orthologs of the *Drosophila* screen hits mapping to the autophagy pathway in pancreatic adenocarcinoma.

Supplementary Table 3: Removal of single genes from K-means clustering survival analysis in Supp. Table 2.
