# Supplementary material for: Autophagy suppresses Ras-driven epithelial tumourigenesis by limiting the accumulation of reactive oxygen species
Source: Oncogene. 2017 Jun 5;36(40):5576–92. doi: 10.1038/onc.2017.175 (PMC5633656; doi:10.1038/onc.2017.175)
Supplement: Supplementary Figures and Tables [file onc2017175x2.pdf]

a

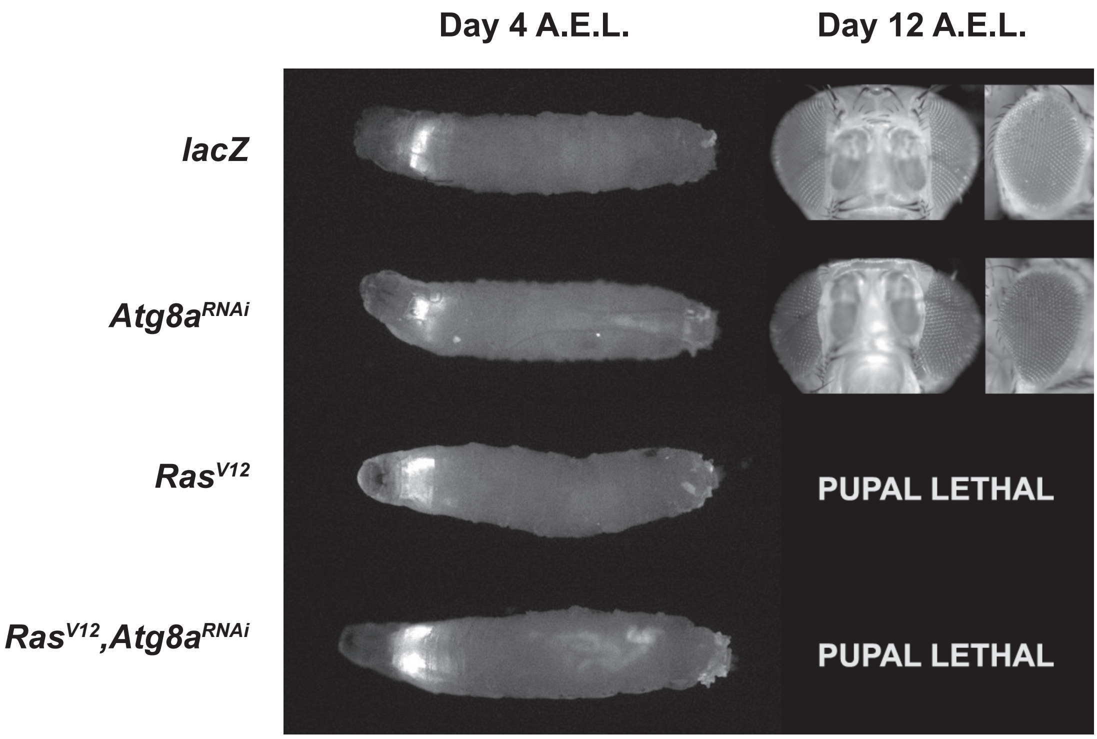

b

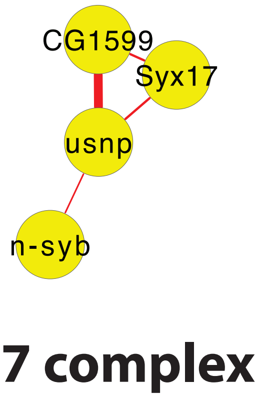

Supplementary Figure 1: Blocking autophagy cooperates with oncogenic Ras in tumourigenesis.
a) Control flies hatch into live, fertile adults, whereas *Ras*^{V12}-expressing individuals die at the pupal stage. b) Proteomics analysis of primary hits identifies a Snap/SNARE protein complex previously involved in autophagy.

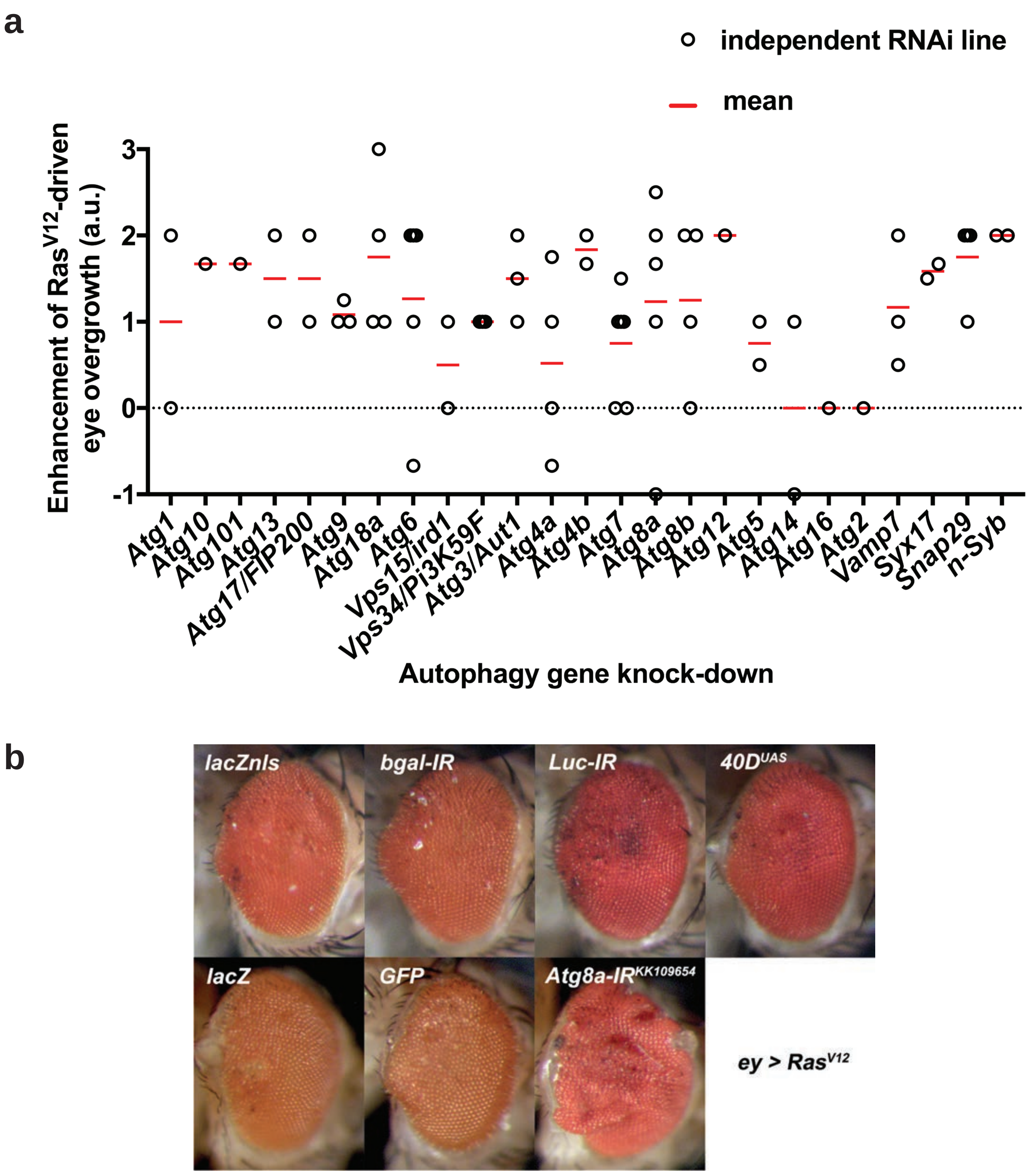

Supplementary Figure 2. Independent RNAi lines targeting the autophagy pathway enhance Ras-driven overgrowth of the adult *Drosophila* eye. a) Individual RNAi lines were screened for their ability to enhance Ras-induced hyperplastic eyes. Adult females were scored as follow: -1 = suppressed compared to control cross, 0=similar to control cross, +1 enhanced compared to control cross, +2/+3 = very/greatly enhanced compared to control cross. Each plot represents an independent RNAi line, and when the same cross was repeated, it represents the average of the different scores (see also Supplementary Table 1). The red line represents the mean enhancement across independent RNAi lines. b) representative side views of *ey-GAL4,UAS-dRas*^{V12} female eyes crossed to different controls or to *Atg8a-IR*. None of the control UAS lines tested significantly altered the size or morphology of Ras^{V12}-expressing adult eyes, unlike expression of *Atg8a* RNAi, that lead to a bigger and more dysmorphic eye (scored 2 in this case).

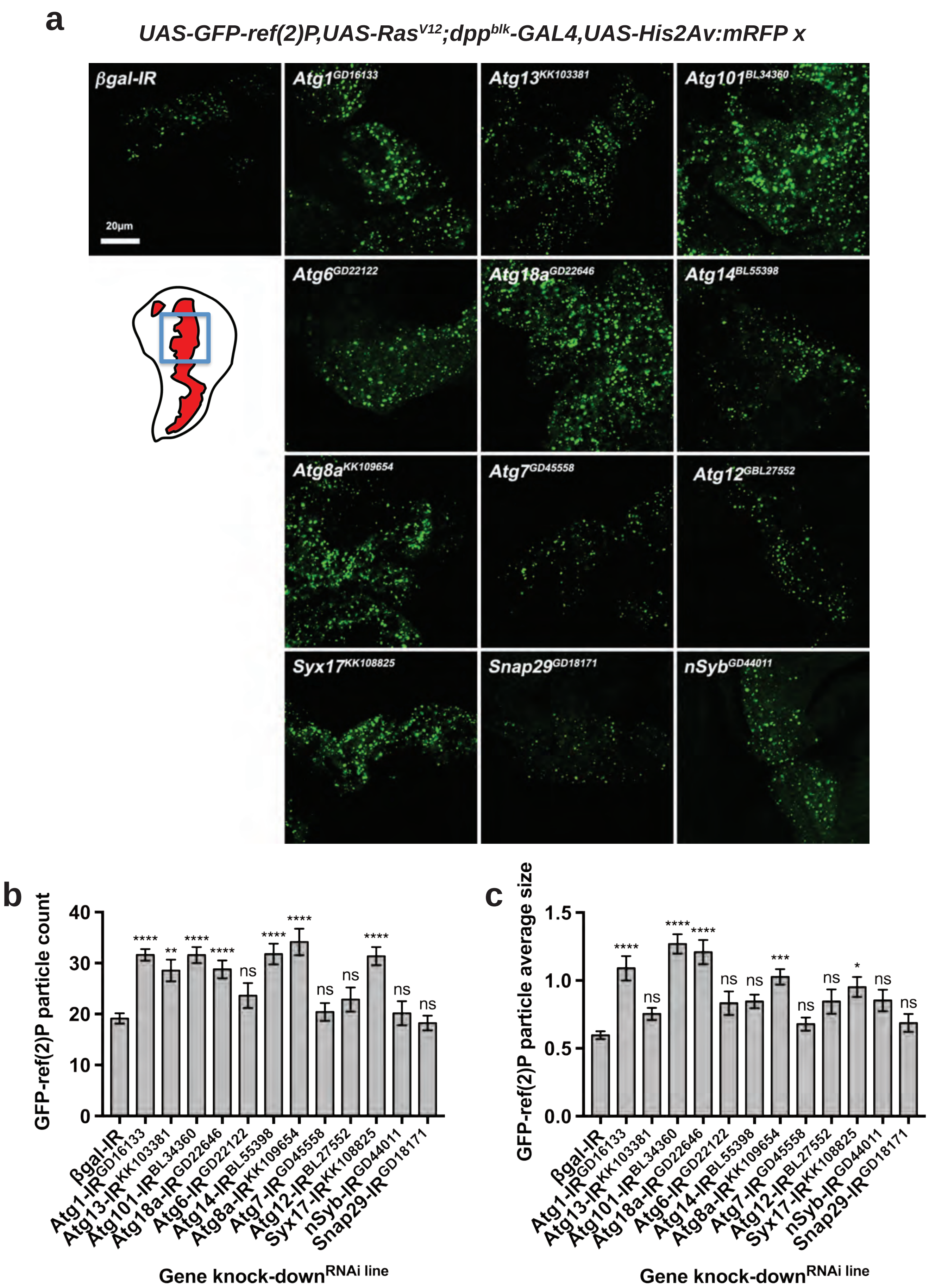

Supplementary Figure 3. Efficient blockage of autophagic flux in Ras-activated epithelial tissue by RNA intereference as seen by monitoring of a GFP-ref(2)P reporter. a) GFP-ref(2)P punctae in *dpp>Ras*^{V12} + RNAis targetting different steps of the autophagy pathway. b) Image J quantification of GFP-ref(2)P particle count and particle average size (c) in a 80x80 px^2 box.
Scale bar: 20µm. Error bars: SEM.
Statistics: one-way ANOVA with Tukey multiple correction, significance as compared to control βgal-IR sample.

Manent et al., Supplementary Figure 4

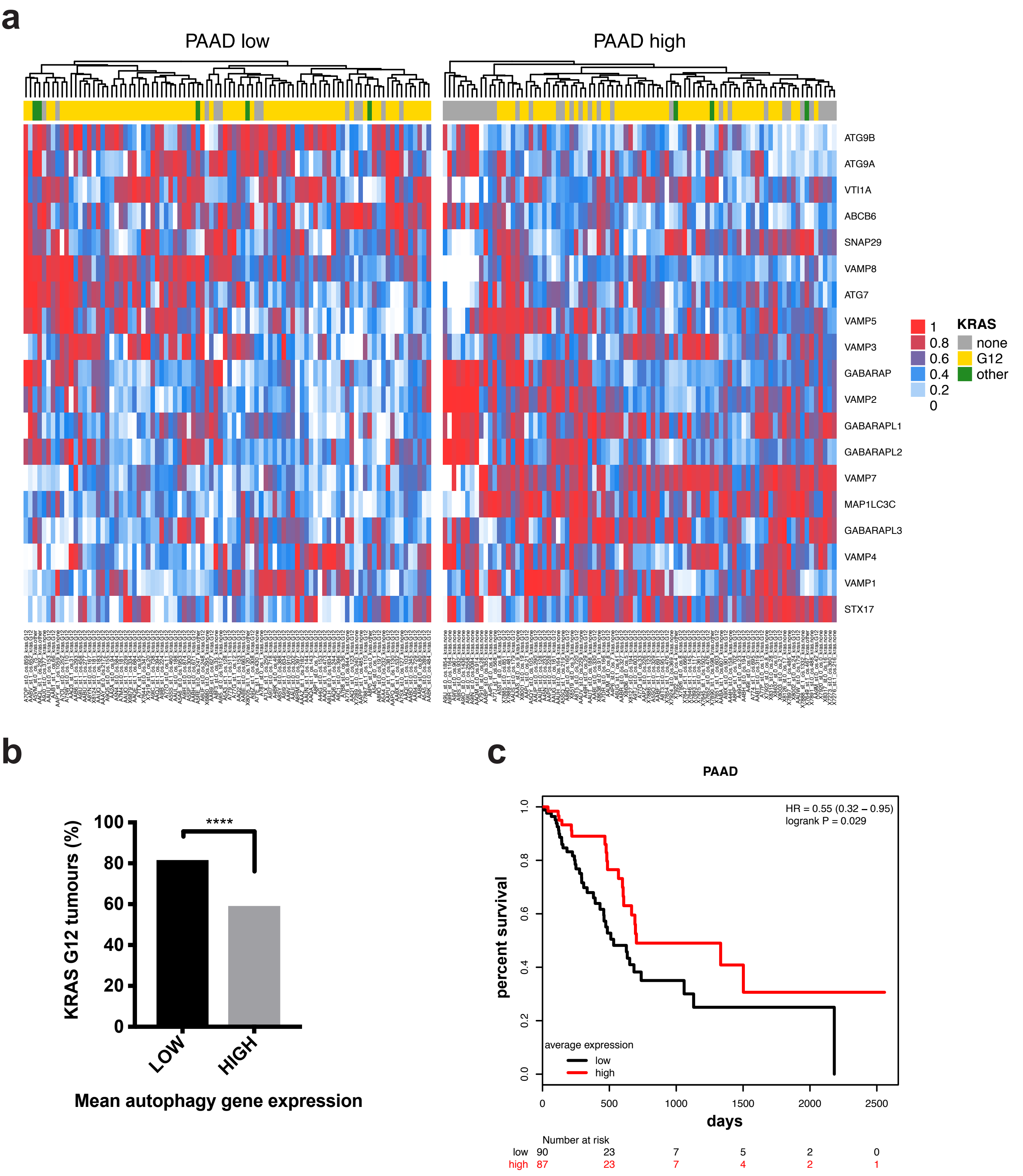

Supplementary Figure 4: Classification of pancreatic ductal adeno carcinomas (PAAD) on the mean expression of autophagy-related genes is predictive of poorer outcome and correlates with higher percentage of *KRAS G12* activating mutations.
a) Heat map of LOG1-ranked expression values for a subset of autophagy genes identified in the Ras-cooperative tumour suppressor screen in *Drosophila*. b) PAAD expressing lower levels of autophagy-related genes are enriched in activating G12 mutations in *KRAS*. c) Survival curves of low (black) and high (red) autophagy-related gene expression groups of PAAD. Lower mean expression of autophagy genes is predictive of poorer survival. Statistics: Fisher's t-test

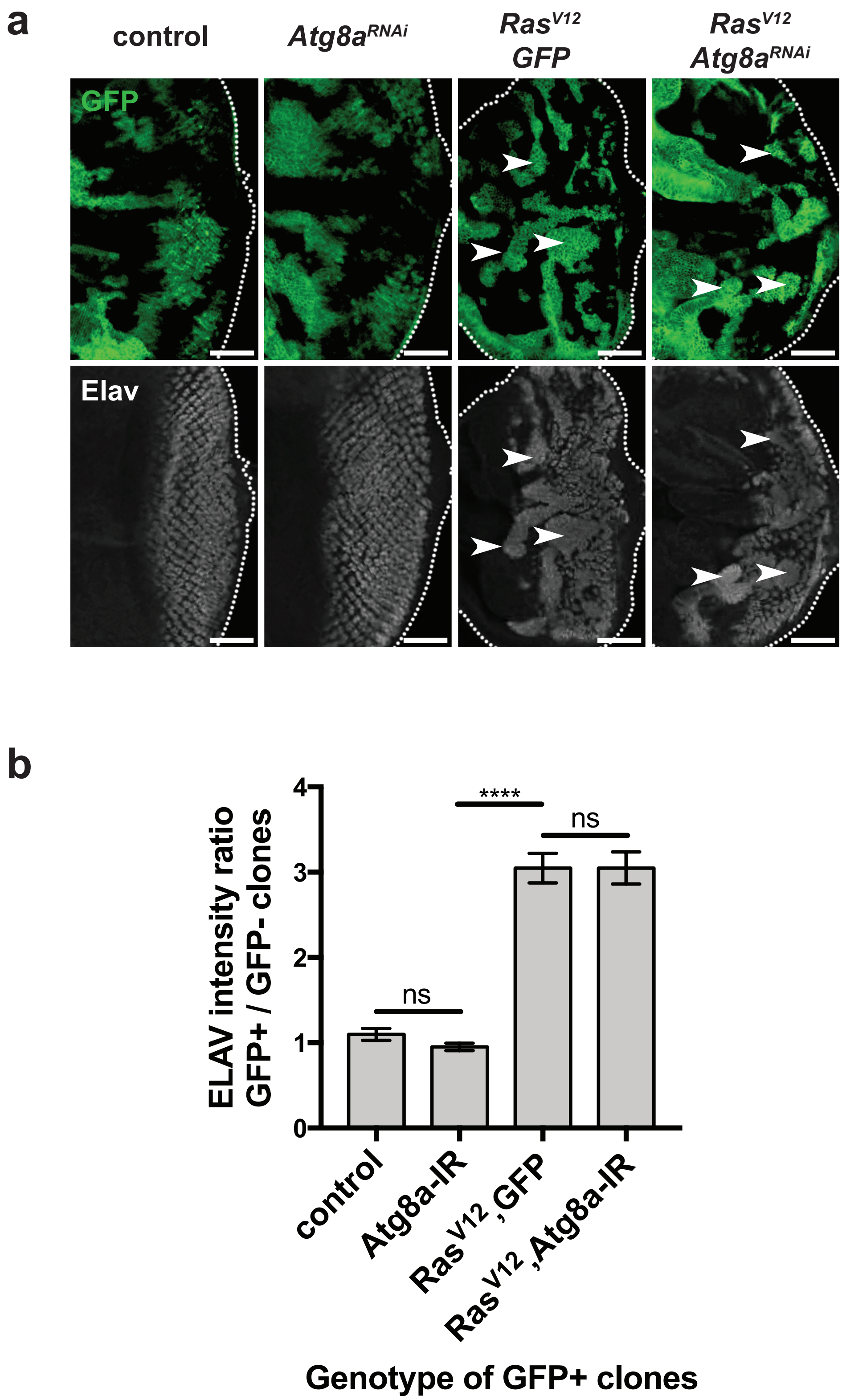

Supplementary Figure 5: Blocking autophagy does not alter Elav expression in *Ras*^{V12}-expressing clones.

a) Elav staining of 3rd instar, mosaic, eye-antennal imaginal discs, showing that as previously documented, ectopic differentiation is observed in *Ras*^{V12}-expressing clones (arrowheads), but that no significant further ectopic differentiation is seen in *Ras*^{V12} *Atg8a*^{RNAi} clones. b), Quantification of Elav levels in the indicated genotypes. Error bars = SEM. Statistics: one-way ANOVA with Tukey test

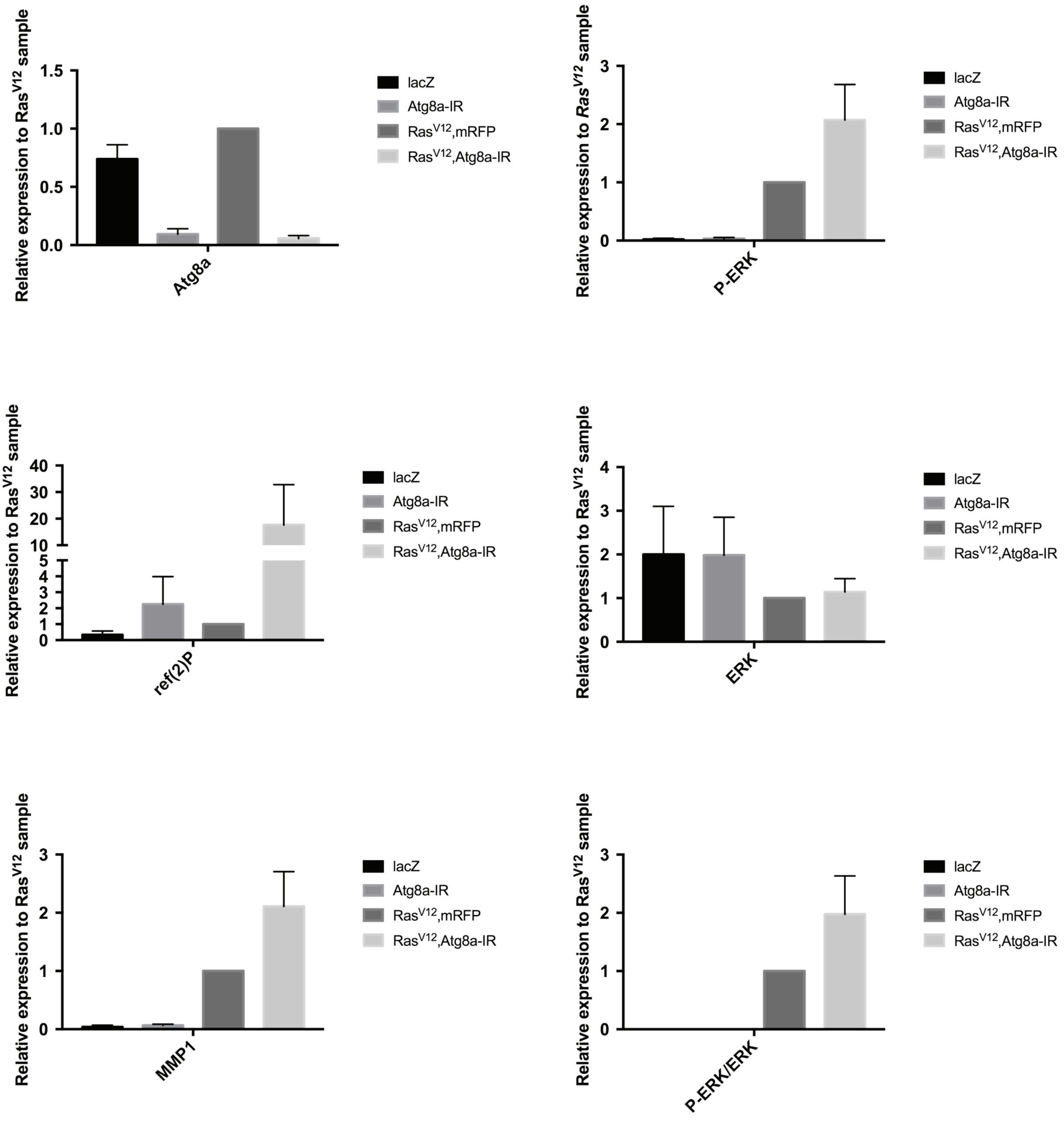

Supplementary Figure 6. Quantification of western blot analysis in the *ey-FLP-out, Act>>GAL4* system at 29°C (Figure 7c). Samples are normalised on a-tubulin, and ratios are calculated on Ras^{V12},mRFP. n=3, error bars: SEM.

a

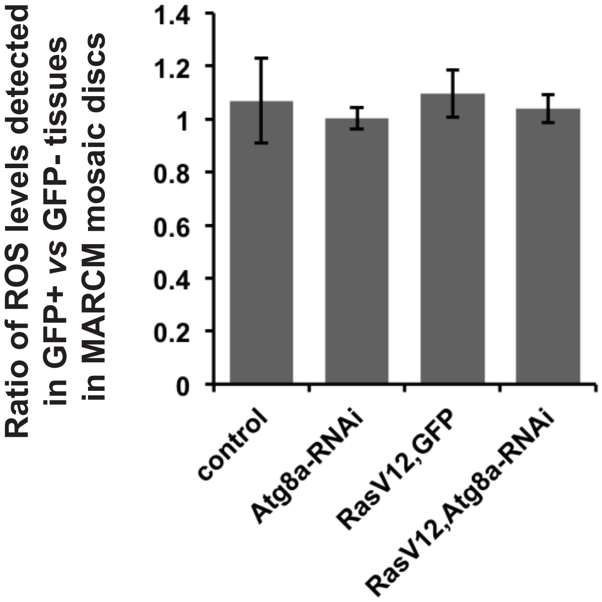

b

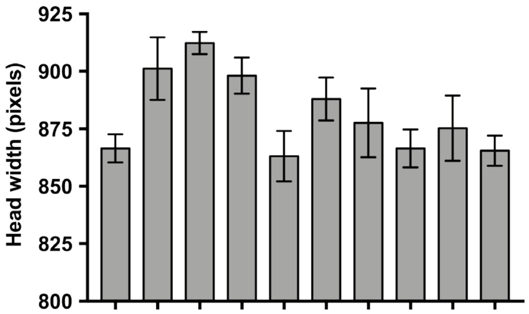

Ras^{V12}	+	+	+	+	+	+	+	+	+	+
GFP	+	+	+	+	-	-	-	-	-	-
bsk^{DN}	-	-	-	-	+	+	+	-	-	-
SOD1	-	-	-	-	-	-	-	+	+	+
lacZ	+	-	-	-	-	-	-	-	-	-
Atg13^{RNAi}	-	+	-	-	+	-	-	+	-	-
Atg8a^{RNAi}	-	-	+	-	-	+	-	-	+	-
Syx17^{RNAi}	-	-	-	+	-	-	+	-	-	+

Supplementary Figure 7. Detection of ROS is not limited to clonal tissue in *Ras^{V12} Atg8a^{RNAi}* mosaic discs and *bsk^{DN}* or *SOD1* overexpression rescues head overgrowth of *Ras^{V12} Atg^{RNAi}* flies.
a) Ratio of clonal to wild-type (GFP+/GFP-) ROS signal in Figure 8 a, b.
b) *bsk^{DN}* or *SOD1* overexpression rescue head overgrowth of *Ras^{V12} Atg^{RNAi}* flies. Adult female flies were photographed from the top, and head width was measured in pixels. *bsk^{DN}*, and even to a greater extent *SOD1* overexpression rescued the head overgrowth due to autophagy inhibition at three different steps of the pathway in Ras-driven overgrowth.
Error bars: SEM. n≥8 per genotype.

Supplementary Table 1- RNAi lines and their *ey*-GAL4,*UAS-Ras*^{V12} enhancement score at 29°C

Drosophila gene	Autophagy process	CG #	Human orthologue	RNAi Stock #	TRiP line	E1	E2	E3	E4	E5	E6	E7	SEM	RNAi Average	RNAi Max
Atg1	Induction	CG10967	ULK1, ULK2	BL26731	JF02273				0					0.00	0.00
Atg1	Induction	CG10967	ULK1, ULK2	GD16133						2			yes	2.00	2.00
Atg10	Completion	CG12821	ATG10	KK106317				1	2	2			yes	1.67	2.00
Atg101	Induction	CG7053	ATG101	BL34360	HMS01349			2	1			2	yes	1.67	2.00
Atg12	Completion	CG10861	ATG12	BL27552	JF02704			2	1	2			yes	1.67	2.00
Atg13	Induction	CG7331	ATG13	GD27956					1					1.00	1.00
Atg13	Induction	CG7331	ATG13	KK103381				2	2	2			yes	2.00	2.00
Atg14	Nucleation	CG11877	ATG14	BL40858	HMS02025							-1		-1.00	-1.00
Atg14	Nucleation	CG11877	ATG14	BL55398	HMC04086							1	yes	1.00	1.00
Atg16	Completion	CG31033	ATG16L1, ATG16L2	BL34358	HMS01347			-1	0					-0.50	0.00
Atg17/FIP200	Induction	CG1347	RB1CC1	BL36918	HMS01611				1					1.00	1.00
Atg17/FIP200	Induction	CG1347	RB1CC1	KK104864					2			2	yes	2.00	2.00
Atg18a	Nucleation	CG7986	WIPI2	BL28061	JF02898				1					1.00	1.00
Atg18a	Nucleation	CG7986	WIPI2	KK105366								1		1.00	1.00
Atg18a	Nucleation	CG7986	WIPI2	BL34714	HMS01193			2	2	3			yes	2.33	3.00
Atg18a	Nucleation	CG7986	WIPI2	GD22646								2	yes	2.00	2.00
Atg2	Nucleation	CG1241	ATG2A, ATG2B	BL27706	JF02786				0					0.00	0.00
Atg3/Aut1	Elongation	CG6877	ATG3	BL34359	HMS01348				1					1.00	1.00
Atg3/Aut1	Elongation	CG6877	ATG3	GD22455					1			2	yes	1.50	2.00
Atg3/Aut1	Elongation	CG6877	ATG3	KK101364								2	yes	2.00	2.00
Atg4a	Elongation	CG4428	ATG4A, ATG4B	BL35740	HMS01482			0	-2	0			yes	-0.67	0.00
Atg4a	Elongation	CG4428	ATG4A, ATG4B	BL28367	JF03003			1	-1					0.00	1.00
Atg4a	Elongation	CG4428	ATG4A, ATG4B	KK107317								1		1.00	1.00
Atg4a	Elongation	CG4428	ATG4A, ATG4B	GD34843				2	1	2		2	yes	1.75	2.00
Atg4b	Elongation	CG6194	ATG4C	GD22294				1	2			2	yes	1.67	2.00
Atg4b	Elongation	CG6194	ATG4C	KK108299								2	yes	2.00	2.00
Atg5	Completion	CG1643	ATG5	BL27551	JF02703				0			1	yes	0.50	1.00
Atg5	Completion	CG1643	ATG5	BL34899	HMS01244				1	1			yes	1.00	1.00
Atg6	Nucleation	CG5429	BECN1	BL35741	HMS01483			-2	1	-1			yes	-0.67	1.00
Atg6	Nucleation	CG5429	BECN1	BL28060	JF02897				1					1.00	1.00
Atg6	Nucleation	CG5429	BECN1	GD22123							2			2.00	2.00
Atg6	Nucleation	CG5429	BECN1	KK110197							2			2.00	2.00
Atg6	Nucleation	CG5429	BECN1	GD22122							2			2.00	2.00
Atg7	Elongation	CG5489	ATG7	BL27707	JF02787				0					0.00	0.00
Atg7	Elongation	CG5489	ATG7	BL34369	HMS01358				0					0.00	0.00
Atg7	Elongation	CG5489	ATG7	GD45560					1					1.00	1.00
Atg7	Elongation	CG5489	ATG7	GD45561					1	1			yes	1.00	1.00
Atg7	Elongation	CG5489	ATG7	GD27432				1	1					1.00	1.00
Atg7	Elongation	CG5489	ATG7	GD45558					1	2			yes	1.50	2.00
Atg8a	Elongation	CG32672	GABARAP	BL34340	HMS01328				-1					-1.00	-1.00
Atg8a	Elongation	CG32672	GABARAP	GD43096		3	1		1					1.67	3.00
Atg8a	Elongation	CG32672	GABARAP	BL28989	JF02895				1					1.00	1.00
Atg8a	Elongation	CG32672	GABARAP	GD43097		2			1	3			yes	2.00	3.00
Atg8a	Elongation	CG32672	GABARAP	KK109654		3	2		2	3			yes	2.50	3.00
Atg8b	Elongation	CG12334	MAP1LC3C, MAP1LC3B2	BL34900	HMS01245				0					0.00	0.00
Atg8b	Elongation	CG12334	MAP1LC3C, MAP1LC3B2	BL27554	JF02706				1	1			yes	1.00	1.00
Atg8b	Elongation	CG12334	MAP1LC3C, MAP1LC3B2	KK101922								2	yes	2.00	2.00
Atg8b	Elongation	CG12334	MAP1LC3C, MAP1LC3B2	GD17079								2	yes	2.00	2.00
Atg9	Induction	CG3615	ATG9A, ATG9B	BL28055	JF02891				1					1.00	1.00
Atg9	Induction	CG3615	ATG9A, ATG9B	BL34901	HMS01246				1	1			yes	1.00	1.00
Atg9	Induction	CG3615	ATG9A, ATG9B	GD10045			1	1	1	2			yes	1.25	2.00
n-Syb	Fusion to lysosome	CG17248	VAMP1/VAMP2/VAMP3	GD49201		2					2			2.00	2.00
n-Syb	Fusion to lysosome	CG17248	VAMP1/VAMP2/VAMP3	GD44011		2					2		yes	2.00	2.00
Syx17	Fusion to lysosome	CG7452	STX17	GD36596		1					2			1.50	2.00
Syx17	Fusion to lysosome	CG7452	STX17	KK108825		1	2				2		yes	1.67	2.00
usnp/Snap29	Fusion to lysosome	CG11173	SNAP29	KK107947							1			1.00	1.00
usnp/Snap29	Fusion to lysosome	CG11173	SNAP29	GD18173							2			2.00	2.00
usnp/Snap29	Fusion to lysosome	CG11173	SNAP29	GD18172							2		yes	2.00	2.00
usnp/Snap29	Fusion to lysosome	CG11173	SNAP29	GD18171							2			2.00	2.00
Vamp7	Fusion to lysosome	CG1599	VAMP7/VAMP8	GD13316		0					1			0.50	1.00
Vamp7	Fusion to lysosome	CG1599	VAMP7/VAMP8	KK108733		1					1			1.00	1.00
Vamp7	Fusion to lysosome	CG1599	VAMP7/VAMP8	GD13317							2		yes	2.00	2.00
Vps15/ird1	Nucleation	CG9746	PIK3R4	BL34092	HMS00908							0		0.00	0.00
Vps15/ird1	Nucleation	CG9746	PIK3R4	BL35209	GL00085							1	yes	1.00	1.00
Vps34/Pi3K59F	Nucleation	CG5373	PIK3C3	BL33384	HMS00261							1		1.00	1.00
Vps34/Pi3K59F	Nucleation	CG5373	PIK3C3	BL36056	GL00175							1		1.00	1.00
Vps34/Pi3K59F	Nucleation	CG5373	PIK3C3	KK100296							1	1	yes	1.00	1.00
UAS-GFP	Control			GFP		0	0				0		yes	0.00	0.00
UAS-lacZ	Control			lacZ		0	0	0	0	0	0	0	yes	0.00	0.00

RNAi Stock prefix	***Origin***
GD, KK	VDRC lines
BL	Bloomington/TRiP lines

E1-E7
Independent experiments at 29°C

Scoring

0	Did not enhance RasV12 overgrowth
1-3	Enhanced RasV12 overgrowth
< 0	Suppressed RasV12 overgrowth

Supplementary Table 2: RNAseq data analysis of human orthologs of the *Drosophila* screen hits mapping to the autophagy pathway in pancreatic adenocarcinoma

Gene Symbol	logrankpvalue	padj	hazard	percentile	cutvalue	perc_kraslow	perc_krashigh	kraslow	krashigh	pfisherless	pfishergreater
VAMP2	0.000000263	0.00000526	0.25	0.22	0.88	0.87	0.65	34	90	0.9987	0.005248
GABARAP	0.0000435	0.000435183	0.33	0.49	1.46	0.82	0.59	71	53	0.9998	0.000781
VAMP3	0.000674228	0.004046584	2.41	0.64	1.17	0.64	0.81	72	52	0.01031	0.9963
GABARAPL2	0.000809317	0.004046584	0.26	0.8	1.09	0.74	0.53	105	19	0.9962	0.01124
GABARAPL3	0.014118147	0.028236294	0.47	0.66	-1.33	0.77	0.57	90	34	0.9983	0.004878
VAMP8	0.005622922	0.014057305	2.24	0.42	1.15	0.57	0.8	42	82	0.0009688	0.9997
VAMP1	0.003418694	0.013674777	0.47	0.34	0.06	0.7	0.7	42	82	0.5612	0.5762
SNAP29	0.004375885	0.014057305	3.95	0.2	0.68	0.72	0.7	26	98	0.6946	0.4612
VAMP7	0.039119741	0.065199569	2.04	0.2	0.65	0.61	0.72	22	102	0.1343	0.9333
ATG7	0.008225008	0.018277795	2.54	0.27	0.7	0.62	0.73	30	94	0.1248	0.9347
GABARAPL1	0.017776079	0.032320144	0.54	0.46	0.99	0.78	0.64	63	61	0.9875	0.02841
ATG9B	0.208773247	0.219761313	0.65	0.21	-0.49	0.57	0.74	21	103	0.03934	0.984
ATG9A	0.143985647	0.169394879	0.66	0.74	1.07	0.72	0.65	94	30	0.8461	0.257
MAP1LC3C	0.11372567	0.142157087	1.52	0.53	-1.1	0.73	0.66	69	55	0.8848	0.192
ABCB6	0.05176681	0.079641246	0.57	0.73	0.56	0.72	0.65	93	31	0.8752	0.215
VTI1A	0.057517787	0.082168267	1.78	0.33	0.4	0.59	0.75	35	89	0.02203	0.9907
STX17	0.2016014	0.219761313	1.44	0.68	0.72	0.75	0.6	90	34	0.9873	0.02926
VAMP5	0.066522577	0.088696769	1.63	0.63	0.62	0.68	0.73	76	48	0.336	0.7779
VAMP4	0.315855313	0.315855313	1.32	0.72	0.54	0.72	0.66	91	33	0.822	0.2864

Table legend

GABARAP	Low expression correlating with poorer outcome and enrichment for KRAS G12 mutations
VAMP8	High expression correlating with poorer outcome and enrichment for KRAS G12 mutations
ATG9B	Low expression correlating with poorer outcome and underepresenation of KRAS G12 mutations

**logrankpvalue**	For multiple percentiles the patient samples were separted into a high and low group.<br>The logrank test allows to compare the survival distribution between two groups.<br>The logrank test p-values represent the loweset p-value that we could find by splitting the samples into a low and high group for each gene.<br>The test is performed for multiple split points and the lowest is choosen.
**padj**	Adjusted p value
**hazard**	Hazard ratio below 1 means that the LOW expressed group is at greater risk for decreased survival (e.g. tumor supressor)<br>Hazard ration above 1 means that the HIGH expressed group is at greater risk for decreased survival (e.g. oncogene)
**percentile**	Fraction of patient samples corresponding to the best split point to define a low expresssion and a high expression group for a particular gene.<br>We defined multiple percentiles between 0.2, 0.25, 0.3,.... 0.8.<br>For example, the first percentile "0.2" means 20 % of the samples with lowest expression for a given gene with the remaining 80% samples designated as group with higher expression
**cutvalue**	The actual expression value that splits a group into low and high expression group and which produced the lowest logranktest p-value.
**perc_kraslow**	Percentage of samples in the low group with kras mutations
**perc_krashigh**	Percentage of samples in the high group with kras mutations
**kraslow**	Actual number of samples with KRAS mutation (G12) in the low group
**krashigh**	Actual number of samples with KRAS mutations in the high group
**pfisherless**	One-sided Fisher's exact test for the underrepresentation of KRAS (G12) mutations in the low expression group
**pfishergreater**	One-sided Fisher's exact test for the overrepresentation of KRAS (G12) mutations in the low expression group

Supplementary Table 3: Removal of single genes from K-means clustering survival analysis in Supp. Table 2

Permutation	logrankpvalue	hazard	kraslow	krashigh	none.otherlow	none.otherhigh	pfisherless	pfishergreater
removed GABARAP	0.8555	0.951861449	68	56	23	30	0.9406	0.1092
removed GABARAPL1	0.7566	0.920339007	65	59	23	30	0.897	0.1748
removed VAMP2	0.7349	1.097102827	69	55	24	29	0.9235	0.1357
removed GABARAPL2	0.5824	0.863221069	65	59	22	31	0.9327	0.1218
removed VAMP4	0.4594	0.819269271	66	58	23	30	0.9135	0.1506
removed ATG7	0.05823	0.599961636	73	51	17	36	0.9997	0.0008994
removed VAMP1	0.05823	0.599961636	73	51	17	36	0.9997	0.0008994
removed VAMP3	0.02953	0.549508748	75	49	16	37	1	0.0001875
removed VAMP8	0.02869	0.551865148	73	51	17	36	0.9997	0.0008994
ALL	0.02869	0.551865148	73	51	17	36	0.9997	0.0008994
removed ATG9B	0.02125	0.538553037	69	55	17	36	0.9989	0.003183
removed ABCB6	0.0158	0.529362004	65	59	13	40	0.9999	0.0004586
removed VAMP5	0.01477	0.516456985	73	51	16	37	0.9999	0.0003905
removed GABARAPL3	0.01394	0.508761357	75	49	16	37	1	0.0001875
removed VTI1A	0.01257	0.499956075	75	49	17	36	0.9999	0.0004504
removed ATG9A	0.0114	0.499977237	73	51	16	37	0.9999	0.0003905
removed SNAP29	0.005411	0.471672739	70	54	17	36	0.9992	0.002354
removed MAP1LC3C	0.004185	0.466435002	64	60	15	38	0.9989	0.003232
removed STX17	0.003597	0.45193371	71	53	17	36	0.9994	0.001725
removed VAMP7	0.001149	0.306682531	96	28	23	30	1	1.47E-05
