# Supplementary material for: Autophagy suppresses Ras-driven epithelial tumourigenesis by limiting the accumulation of reactive oxygen species
Source: Oncogene. 2017 Jun 5;36(40):5576–92. doi: 10.1038/onc.2017.175 (PMC5633656; doi:10.1038/onc.2017.175)
Supplement: Supplementary Data set 1 [file onc2017175x3.pdf]

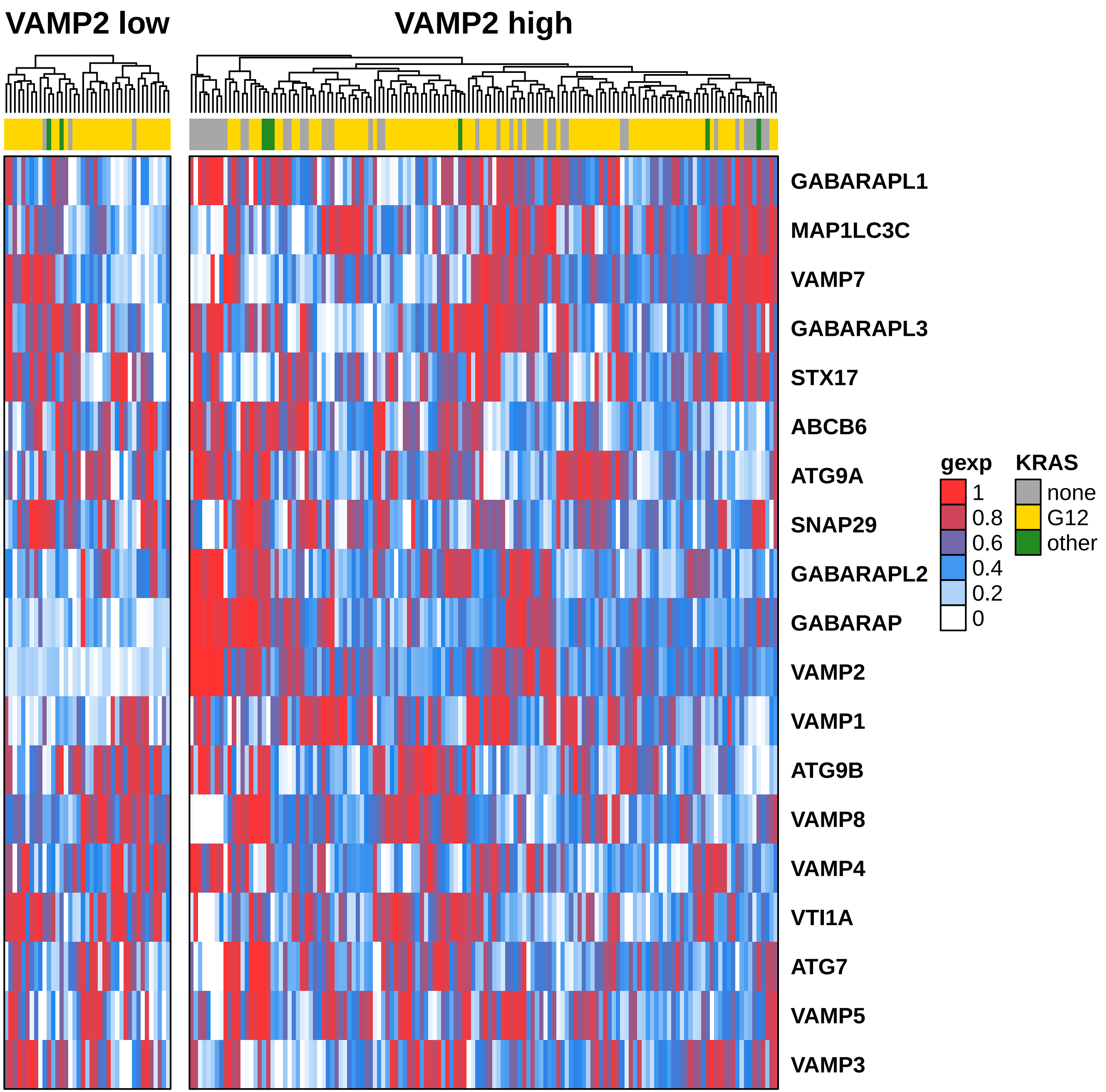

VAMP2 low
VAMP2 high
GABARAPL1
MAP1LC3C
VAMP7
GABARAPL3
STX17
ABCB6
ATG9A
SNAP29
GABARAPL2
GABARAP
VAMP2
VAMP1
ATG9B
VAMP8
VAMP4
VTI1A
ATG7
VAMP5
VAMP3
gexp
1
0.8
0.6
0.4
0.2
0
KRAS
none
G12
other

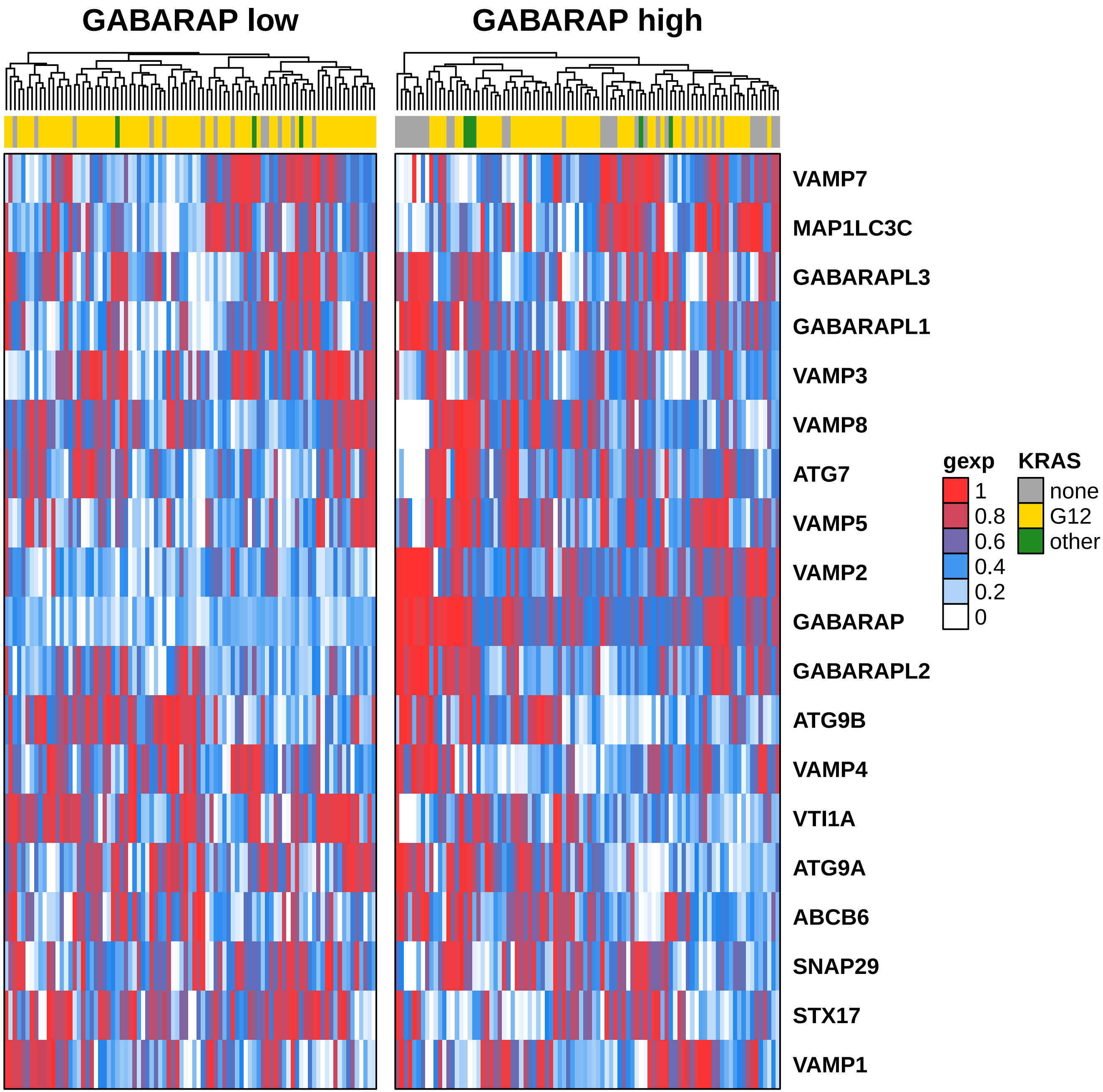
GABARAP low
GABARAP high
VAMP7
MAP1LC3C
GABARAPL3
GABARAPL1
VAMP3
VAMP8
ATG7
VAMP5
VAMP2
GABARAP
GABARAPL2
ATG9B
VAMP4
VTI1A
ATG9A
ABCB6
SNAP29
STX17
VAMP1
gexp
1
0.8
0.6
0.4
0.2
0
KRAS
none
G12
other

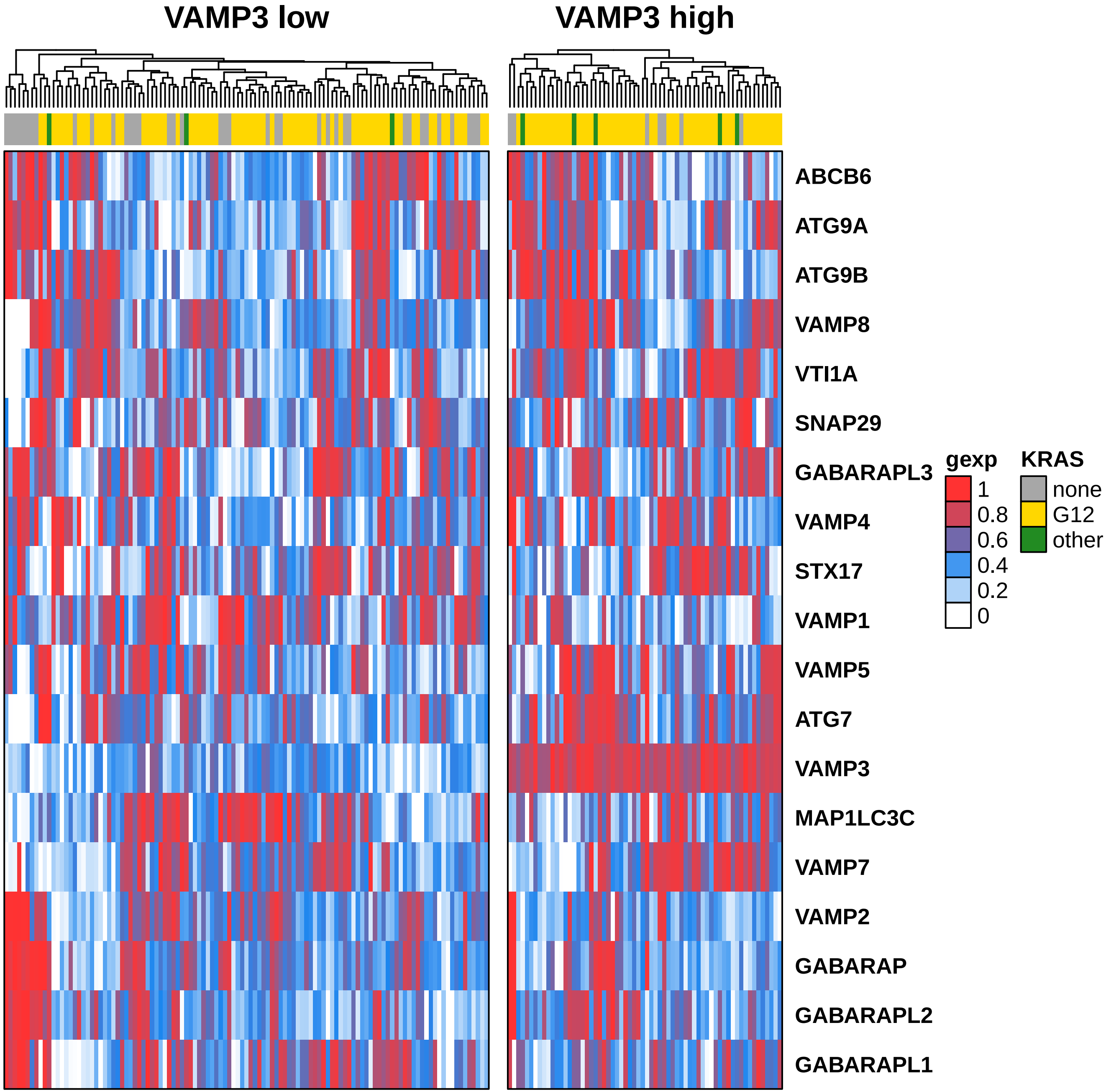

VAMP3 low
VAMP3 high
ABCB6
ATG9A
ATG9B
VAMP8
VTI1A
SNAP29
GABARAPL3
VAMP4
STX17
VAMP1
VAMP5
ATG7
VAMP3
MAP1LC3C
VAMP7
VAMP2
GABARAP
GABARAPL2
GABARAPL1
gexp
1
0.8
0.6
0.4
0.2
0
KRAS
none
G12
other

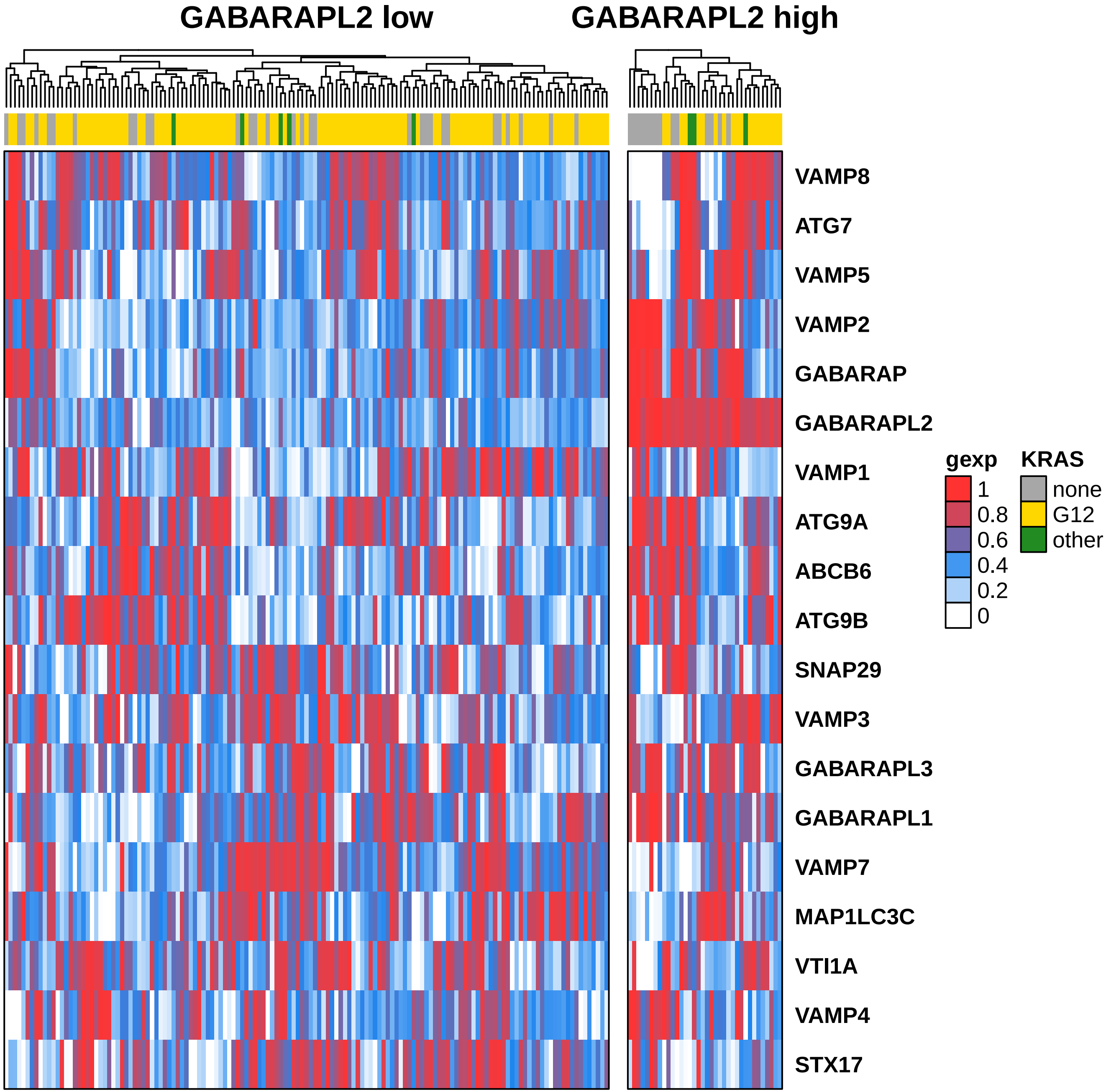

GABARAPL2 low
GABARAPL2 high
VAMP8
ATG7
VAMP5
VAMP2
GABARAP
GABARAPL2
VAMP1
ATG9A
ABCB6
ATG9B
SNAP29
VAMP3
GABARAPL3
GABARAPL1
VAMP7
MAP1LC3C
VTI1A
VAMP4
STX17
gexp
1
0.8
0.6
0.4
0.2
0
KRAS
none
G12
other

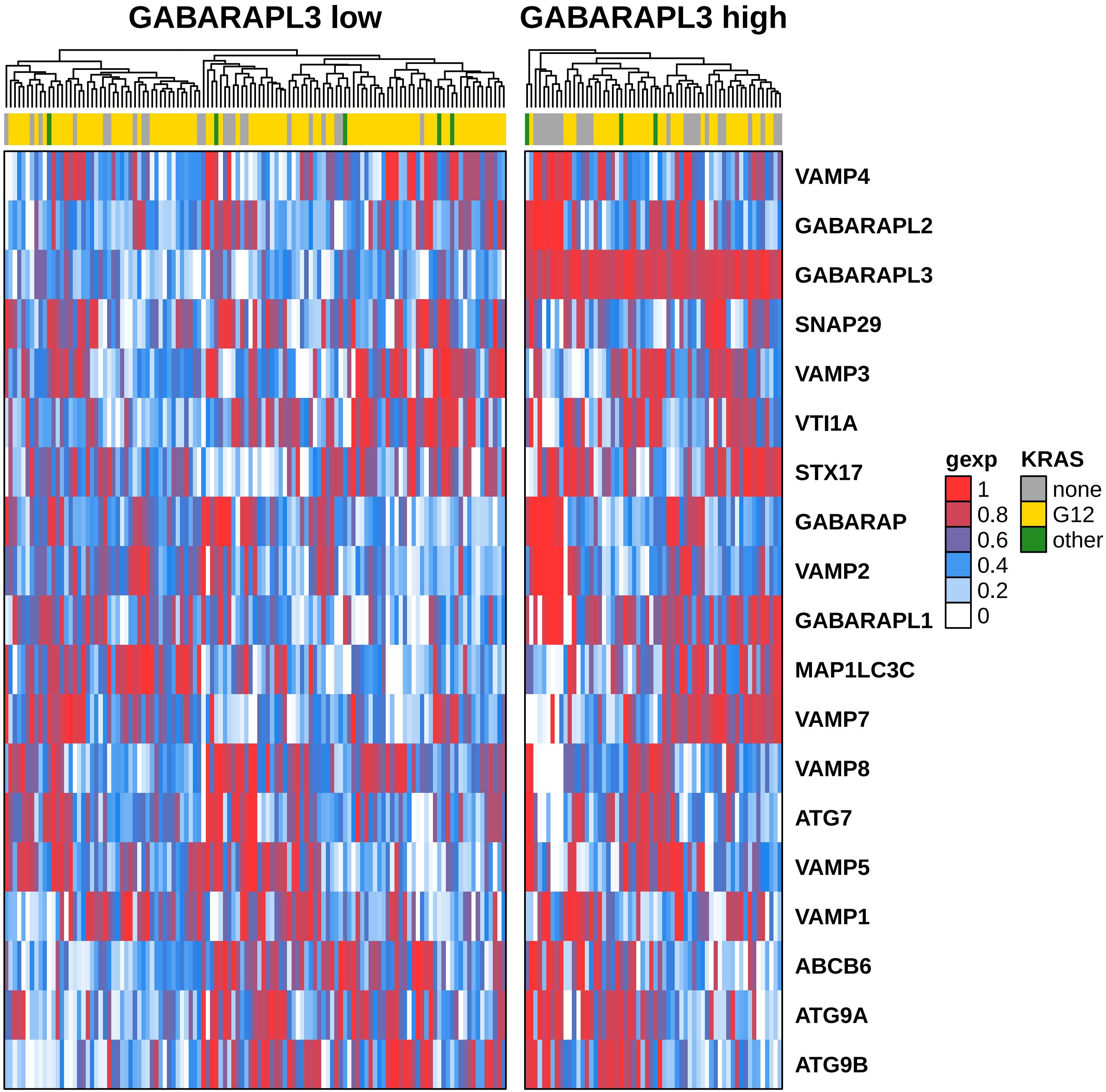
GABARAPL3 low
GABARAPL3 high
VAMP4
GABARAPL2
GABARAPL3
SNAP29
VAMP3
VTI1A
STX17
GABARAP
VAMP2
GABARAPL1
MAP1LC3C
VAMP7
VAMP8
ATG7
VAMP5
VAMP1
ABCB6
ATG9A
ATG9B
gexp
1
0.8
0.6
0.4
0.2
0
KRAS
none
G12
other

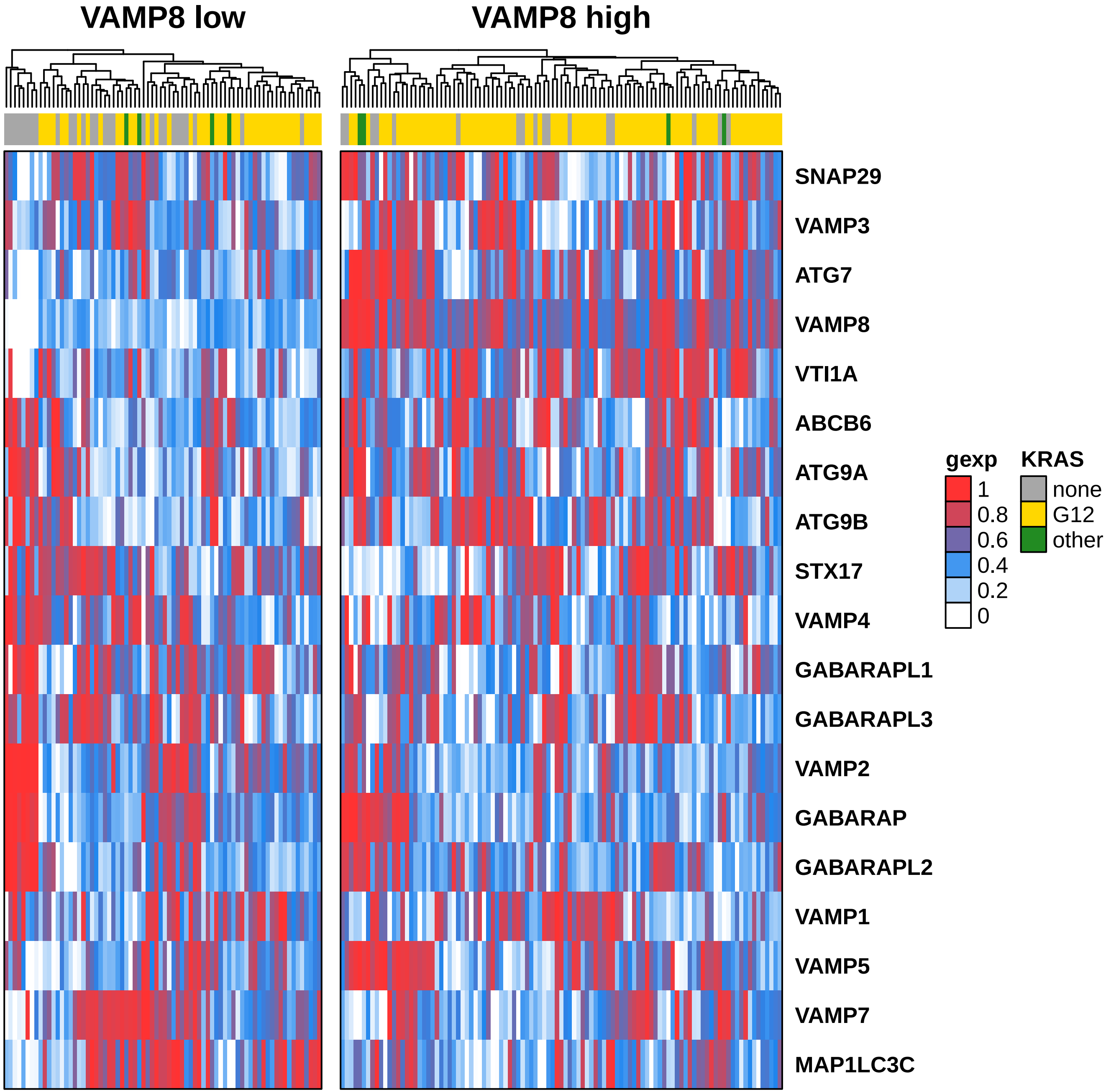
VAMP8 low
VAMP8 high
SNAP29
VAMP3
ATG7
VAMP8
VTI1A
ABCB6
ATG9A
ATG9B
STX17
VAMP4
GABARAPL1
GABARAPL3
VAMP2
GABARAP
GABARAPL2
VAMP1
VAMP5
VAMP7
MAP1LC3C
gexp
1
0.8
0.6
0.4
0.2
0
KRAS
none
G12
other

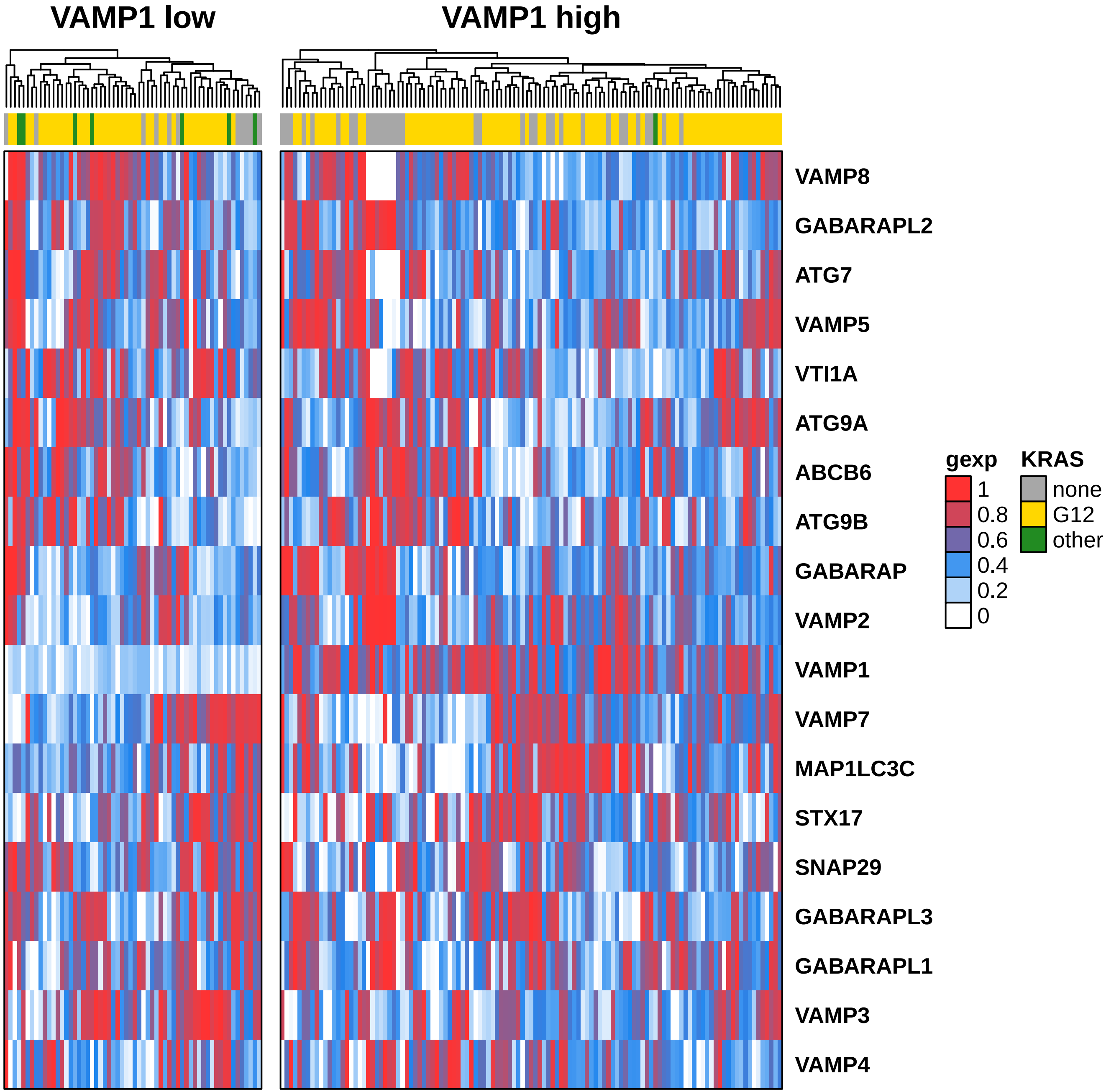
VAMP1 low
VAMP1 high
VAMP8
GABARAPL2
ATG7
VAMP5
VTI1A
ATG9A
ABCB6
ATG9B
GABARAP
VAMP2
VAMP1
VAMP7
MAP1LC3C
STX17
SNAP29
GABARAPL3
GABARAPL1
VAMP3
VAMP4
gexp
1
0.8
0.6
0.4
0.2
0
KRAS
none
G12
other

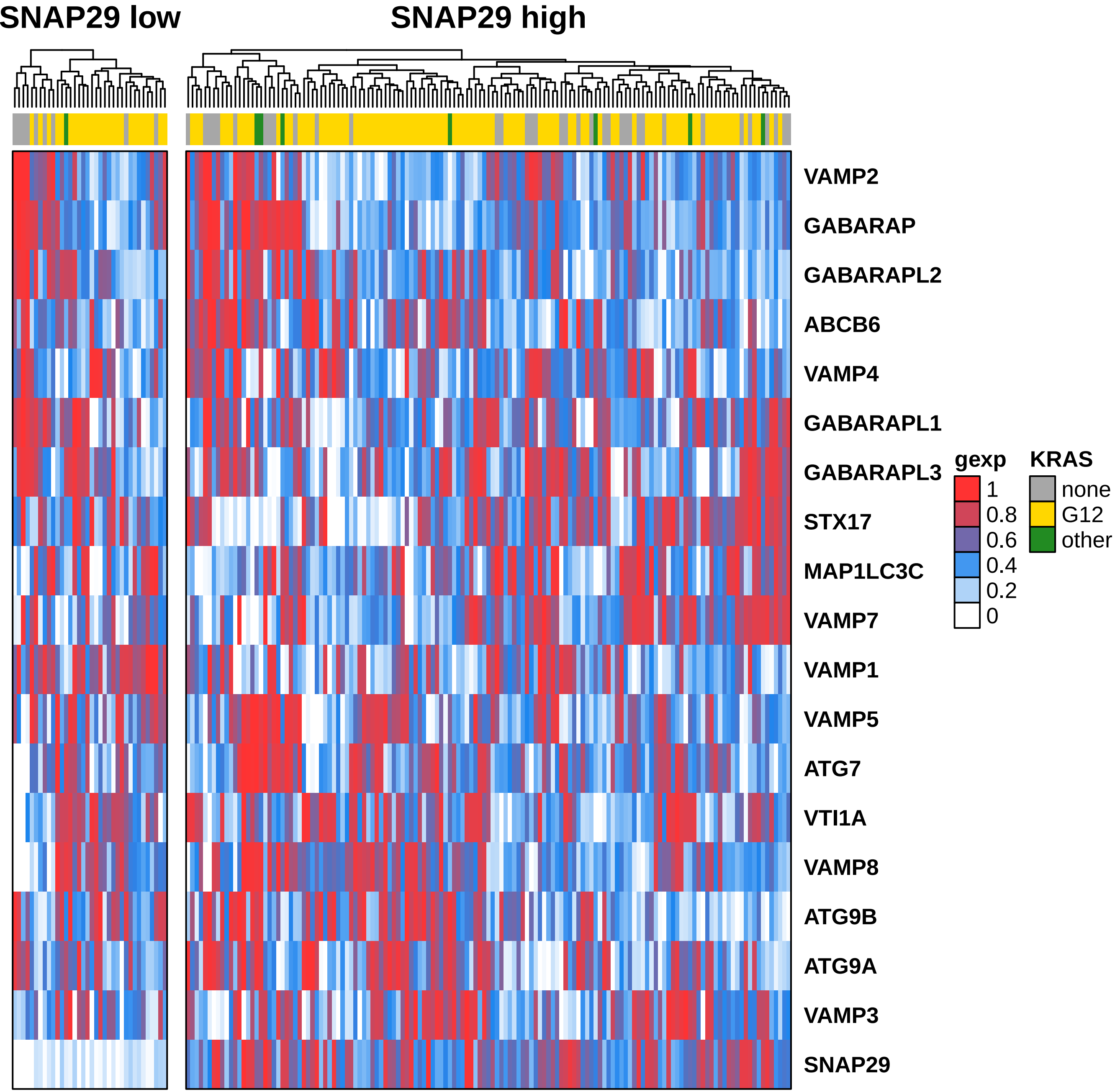
SNAP29 low
SNAP29 high
VAMP2
GABARAP
GABARAPL2
ABCB6
VAMP4
GABARAPL1
GABARAPL3
STX17
MAP1LC3C
VAMP7
VAMP1
VAMP5
ATG7
VTI1A
VAMP8
ATG9B
ATG9A
VAMP3
SNAP29
gexp
1
0.8
0.6
0.4
0.2
0
KRAS
none
G12
other

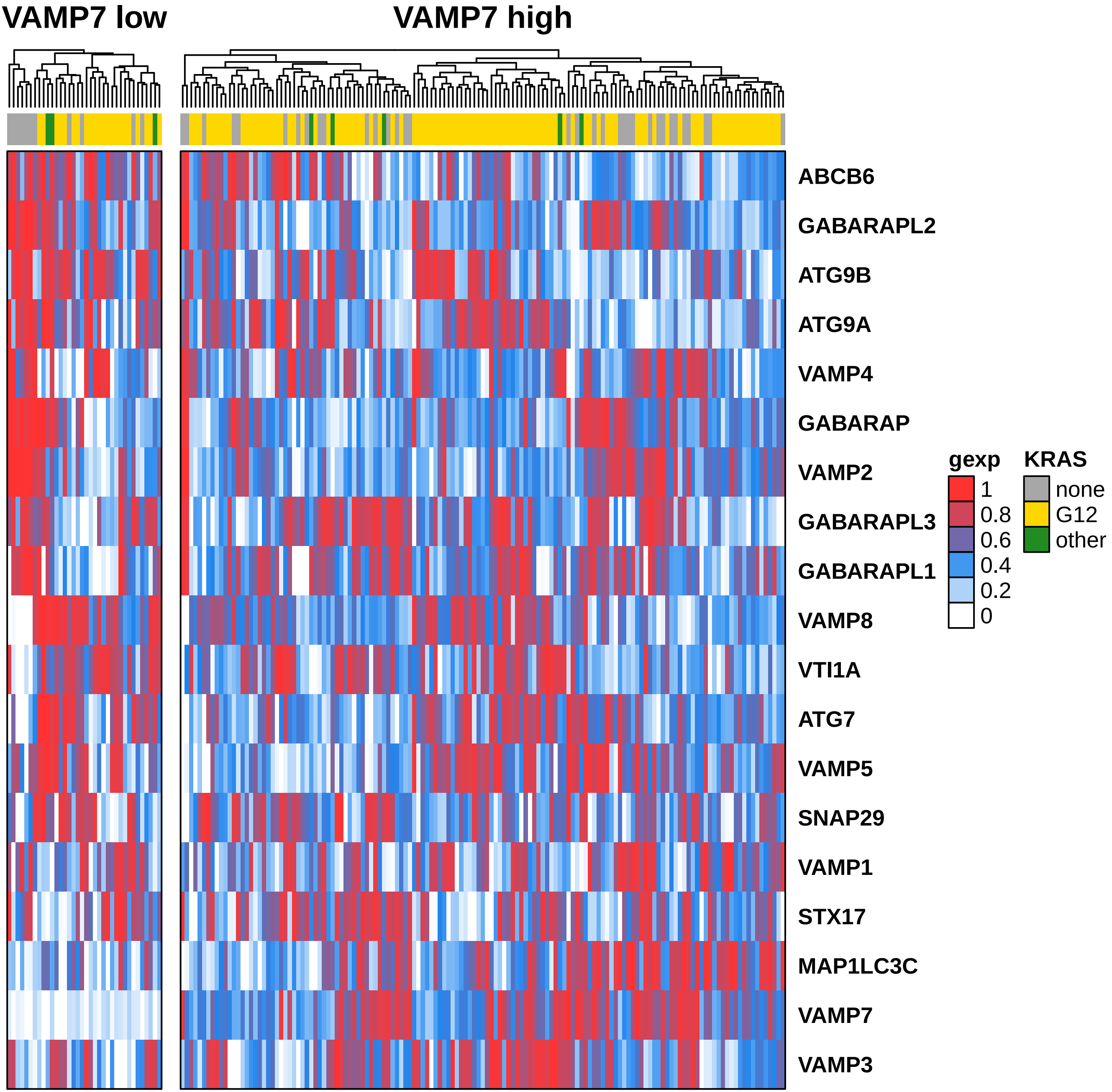
VAMP7 low
VAMP7 high
ABCB6
GABARAPL2
ATG9B
ATG9A
VAMP4
GABARAP
VAMP2
GABARAPL3
GABARAPL1
VAMP8
VTI1A
ATG7
VAMP5
SNAP29
VAMP1
STX17
MAP1LC3C
VAMP7
VAMP3
gexp
1
0.8
0.6
0.4
0.2
0
KRAS
none
G12
other

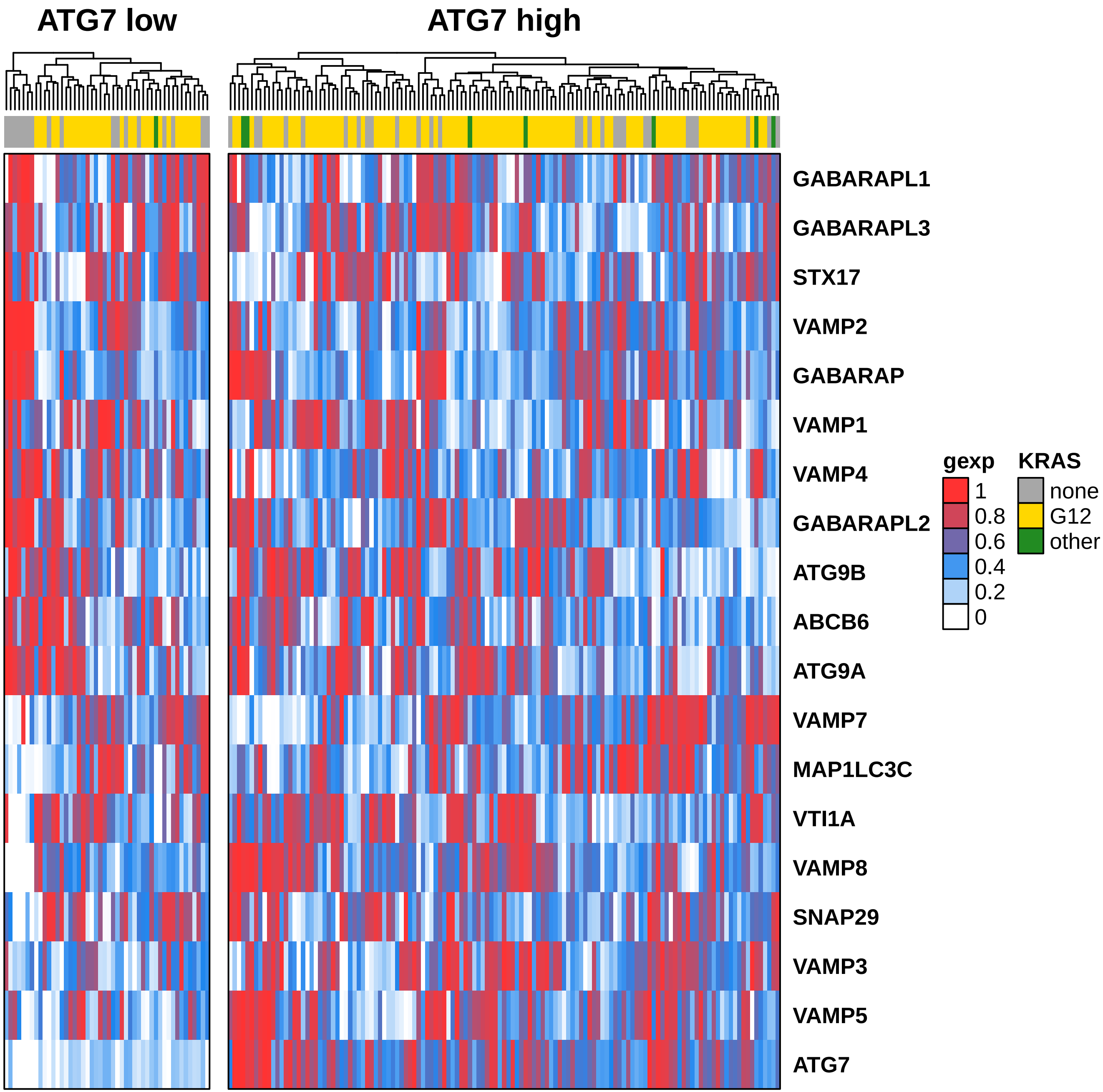
ATG7 low
ATG7 high
GABARAPL1
GABARAPL3
STX17
VAMP2
GABARAP
VAMP1
VAMP4
GABARAPL2
ATG9B
ABCB6
ATG9A
VAMP7
MAP1LC3C
VTI1A
VAMP8
SNAP29
VAMP3
VAMP5
ATG7
gexp
1
0.8
0.6
0.4
0.2
0
KRAS
none
G12
other

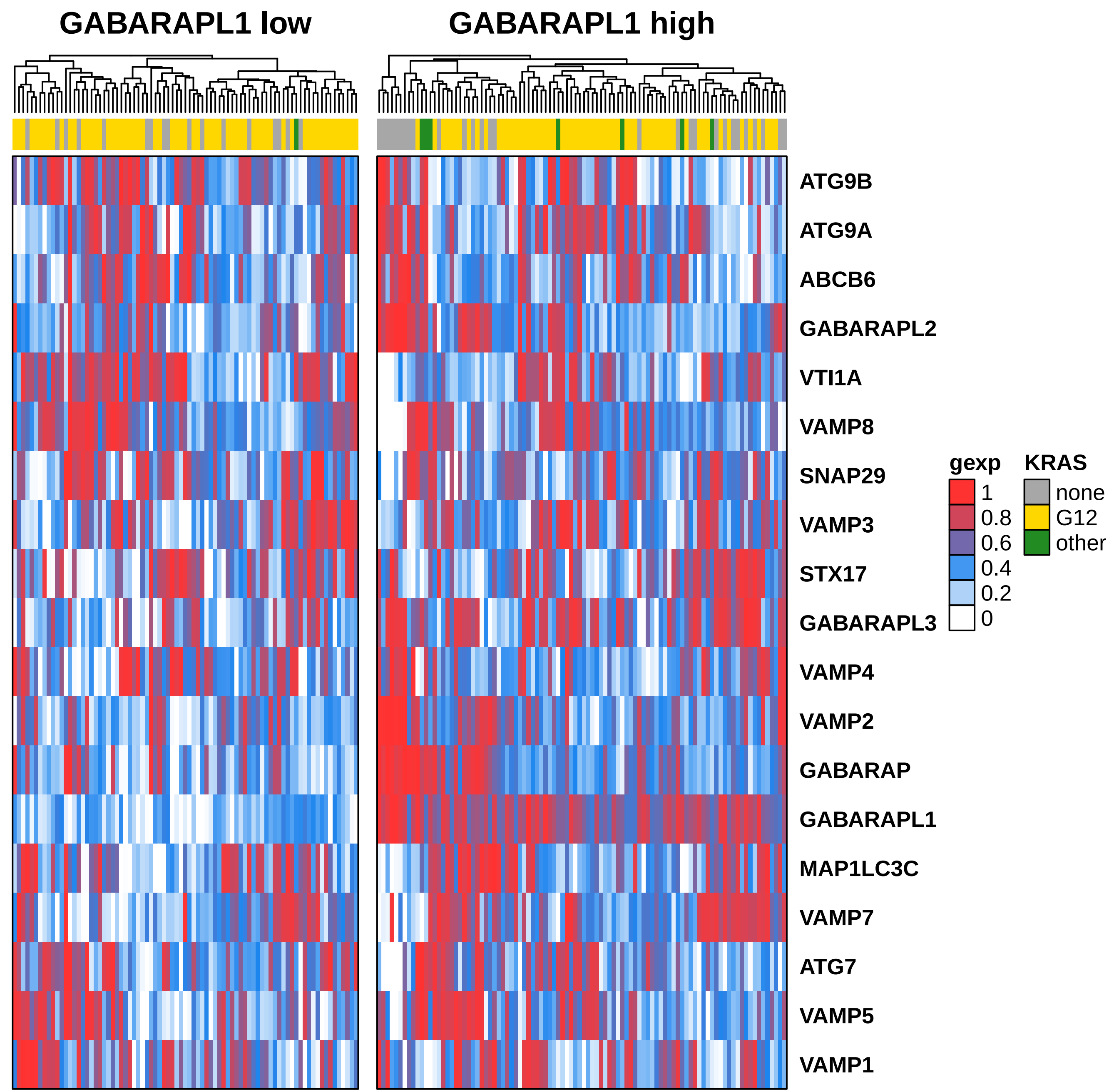
GABARAPL1 low
GABARAPL1 high
ATG9B
ATG9A
ABCB6
GABARAPL2
VTI1A
VAMP8
SNAP29
VAMP3
STX17
GABARAPL3
VAMP4
VAMP2
GABARAP
GABARAPL1
MAP1LC3C
VAMP7
ATG7
VAMP5
VAMP1
gexp
1
0.8
0.6
0.4
0.2
0
KRAS
none
G12
other

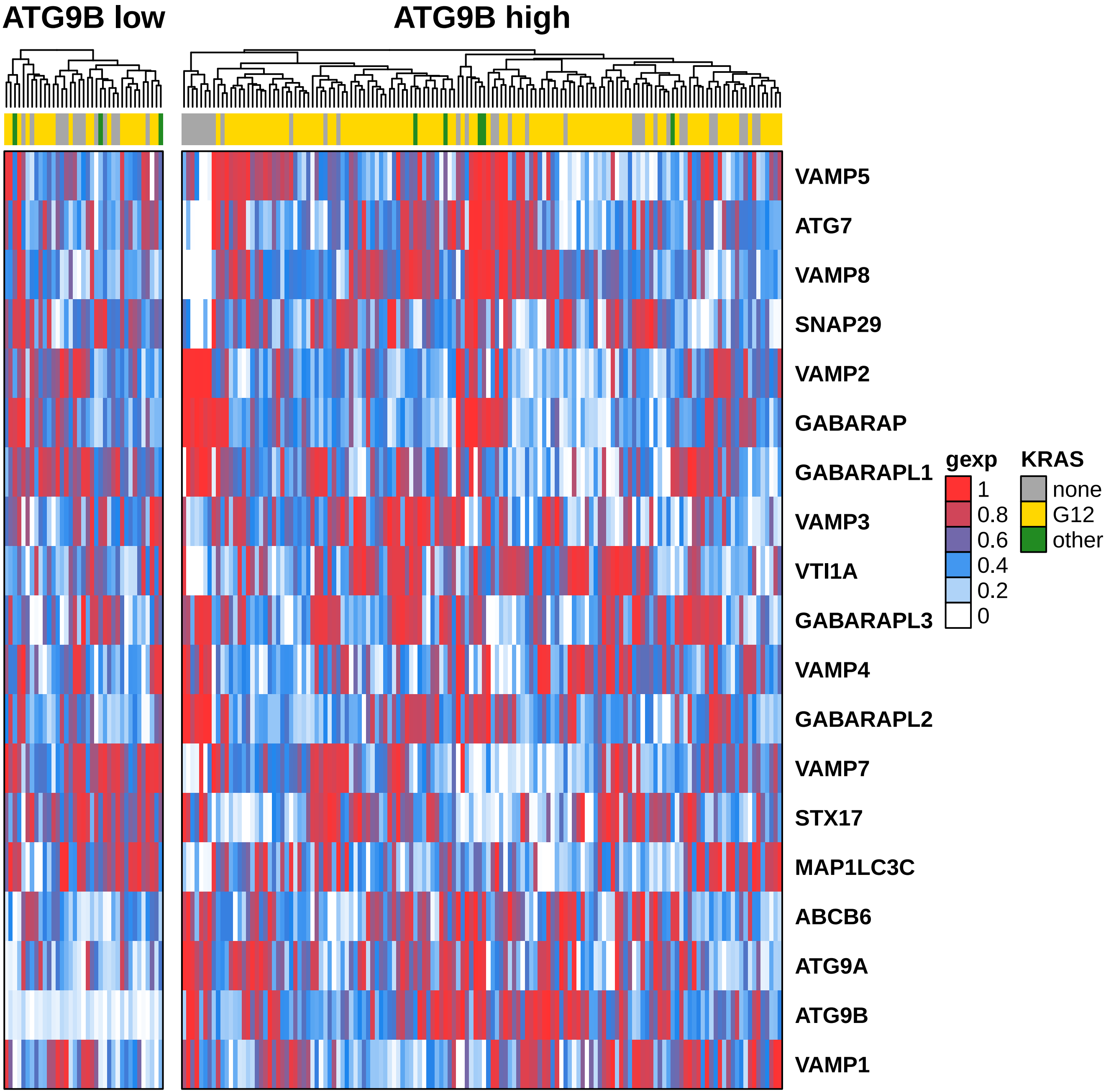

ATG9B low
ATG9B high
VAMP5
ATG7
VAMP8
SNAP29
VAMP2
GABARAP
GABARAPL1
VAMP3
VTI1A
GABARAPL3
VAMP4
GABARAPL2
VAMP7
STX17
MAP1LC3C
ABCB6
ATG9A
ATG9B
VAMP1
gexp
1
0.8
0.6
0.4
0.2
0
KRAS
none
G12
other

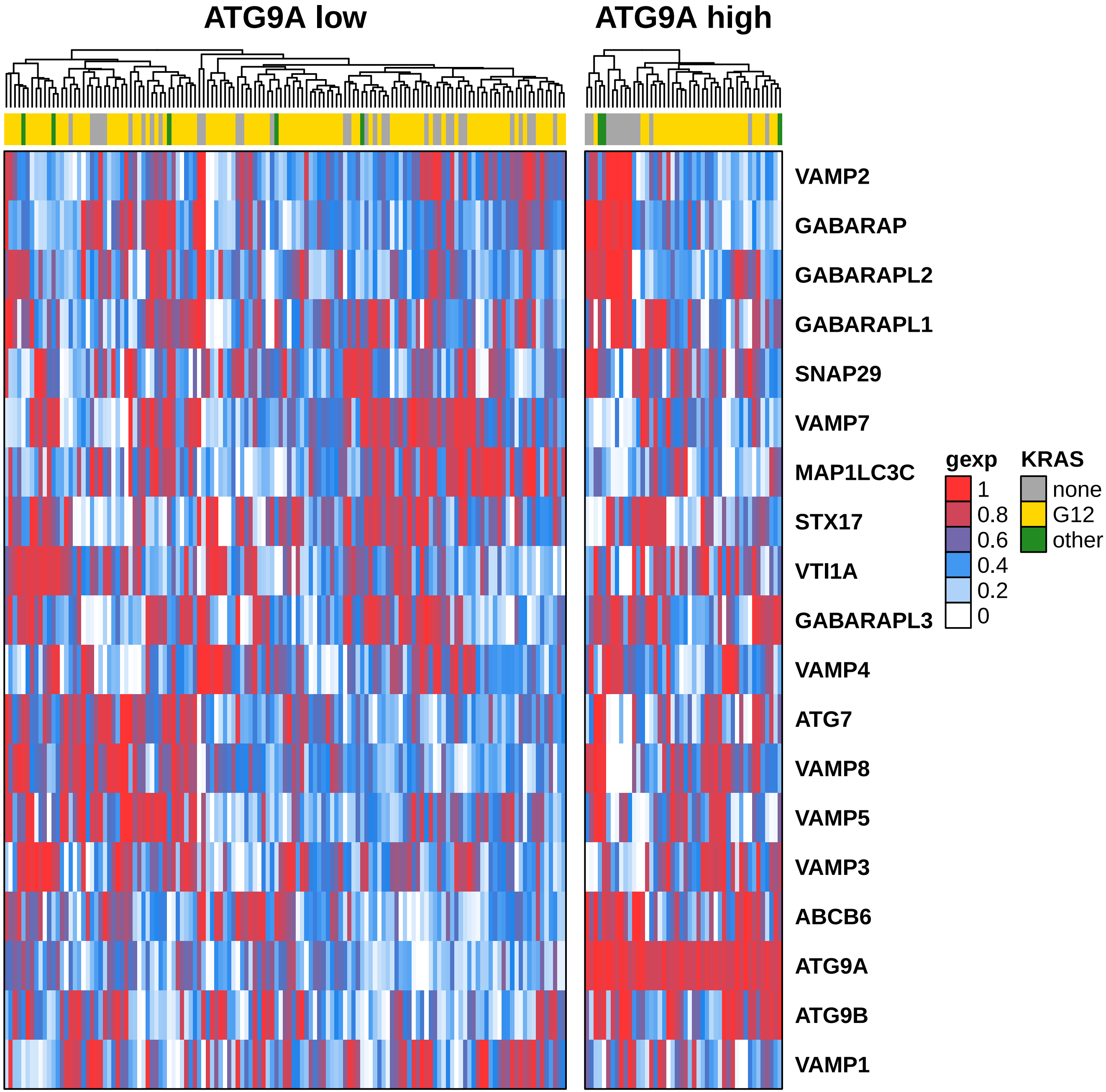
ATG9A low
ATG9A high
VAMP2
GABARAP
GABARAPL2
GABARAPL1
SNAP29
VAMP7
MAP1LC3C
STX17
VTI1A
GABARAPL3
VAMP4
ATG7
VAMP8
VAMP5
VAMP3
ABCB6
ATG9A
ATG9B
VAMP1
gexp
1
0.8
0.6
0.4
0.2
0
KRAS
none
G12
other

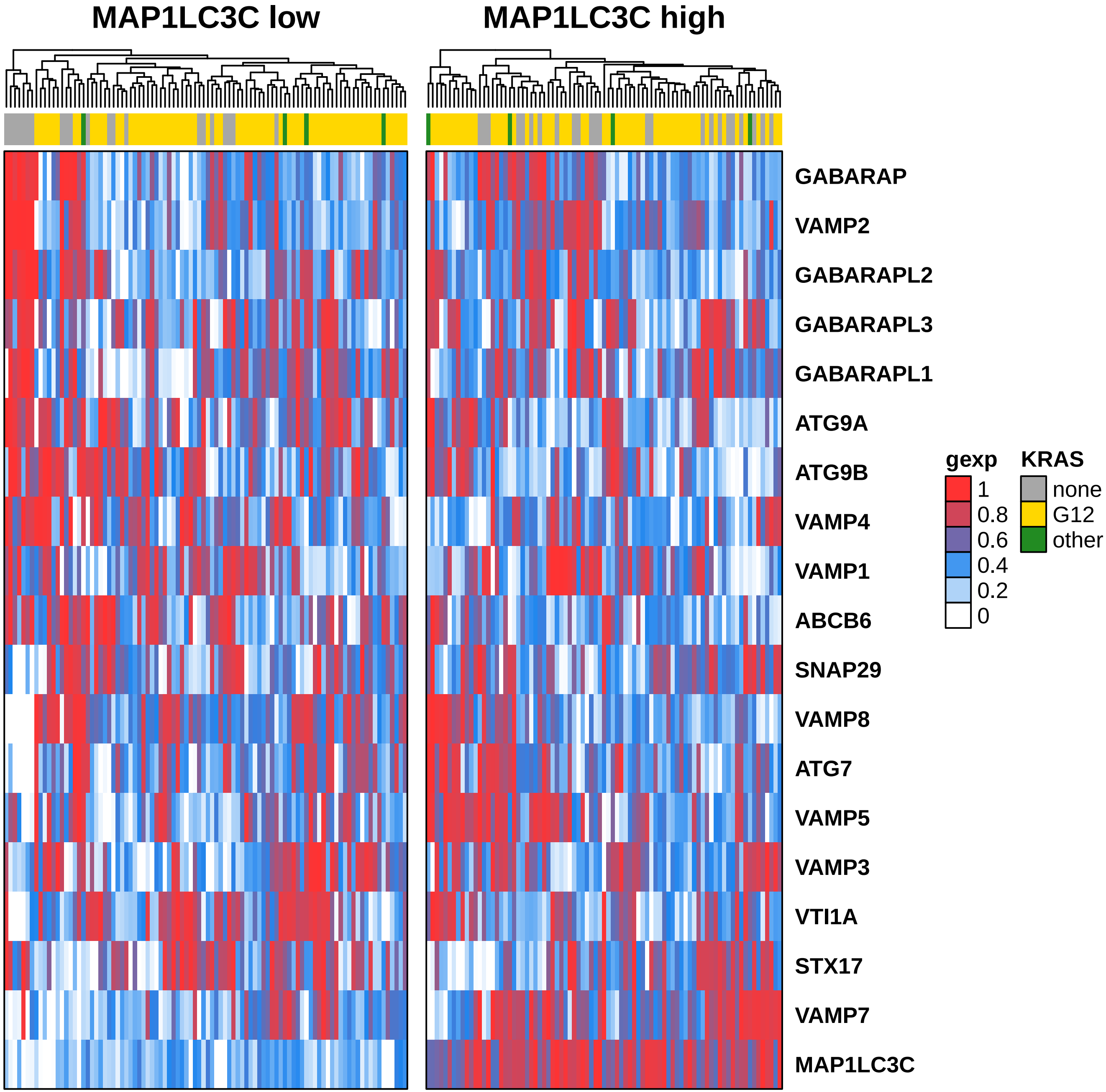
MAP1LC3C low
MAP1LC3C high
GABARAP
VAMP2
GABARAPL2
GABARAPL3
GABARAPL1
ATG9A
ATG9B
VAMP4
VAMP1
ABCB6
SNAP29
VAMP8
ATG7
VAMP5
VAMP3
VTI1A
STX17
VAMP7
MAP1LC3C
gexp
1
0.8
0.6
0.4
0.2
0
KRAS
none
G12
other

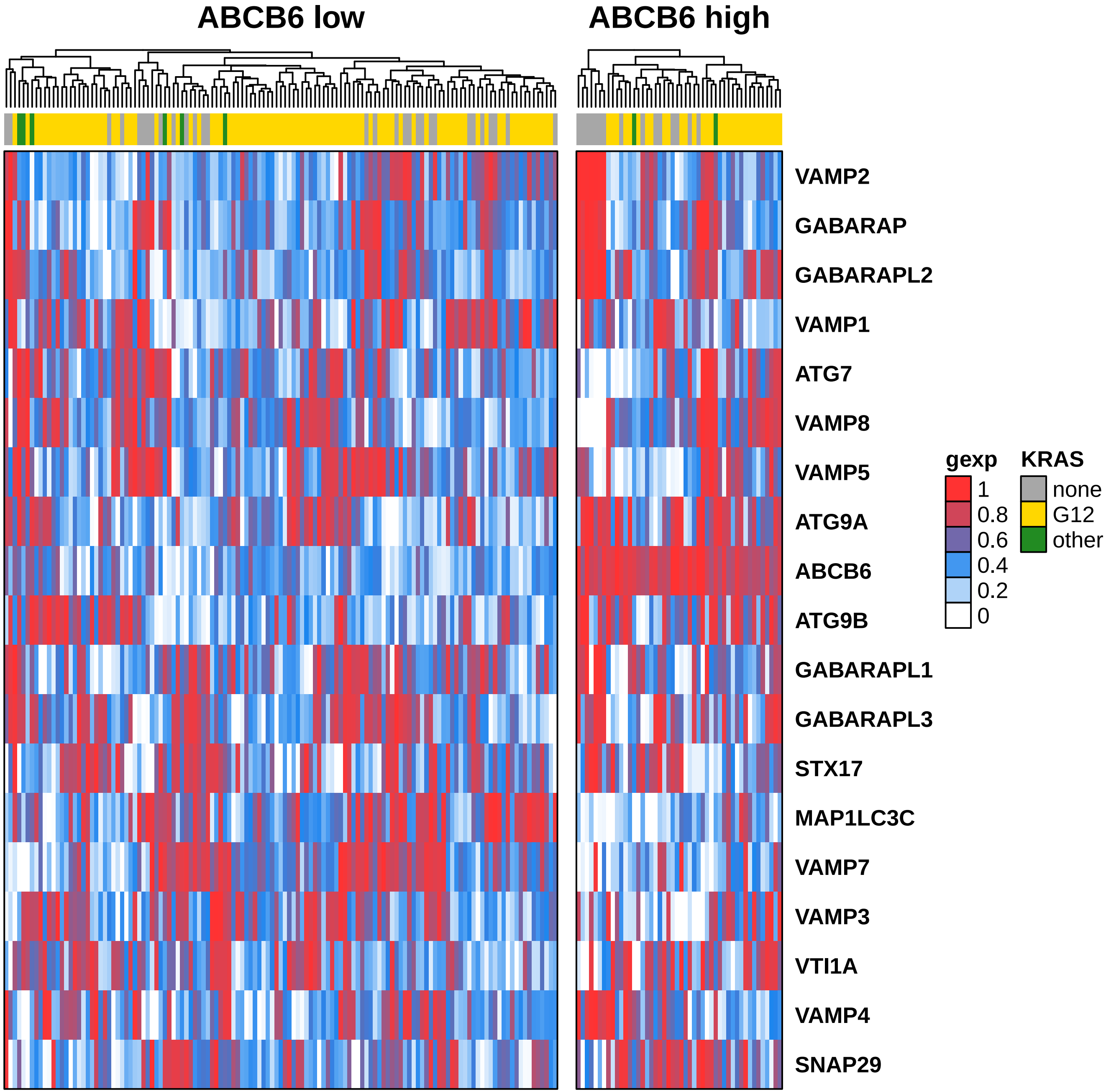

ABCB6 low
ABCB6 high
VAMP2
GABARAP
GABARAPL2
VAMP1
ATG7
VAMP8
VAMP5
ATG9A
ABCB6
ATG9B
GABARAPL1
GABARAPL3
STX17
MAP1LC3C
VAMP7
VAMP3
VTI1A
VAMP4
SNAP29
gexp
1
0.8
0.6
0.4
0.2
0
KRAS
none
G12
other

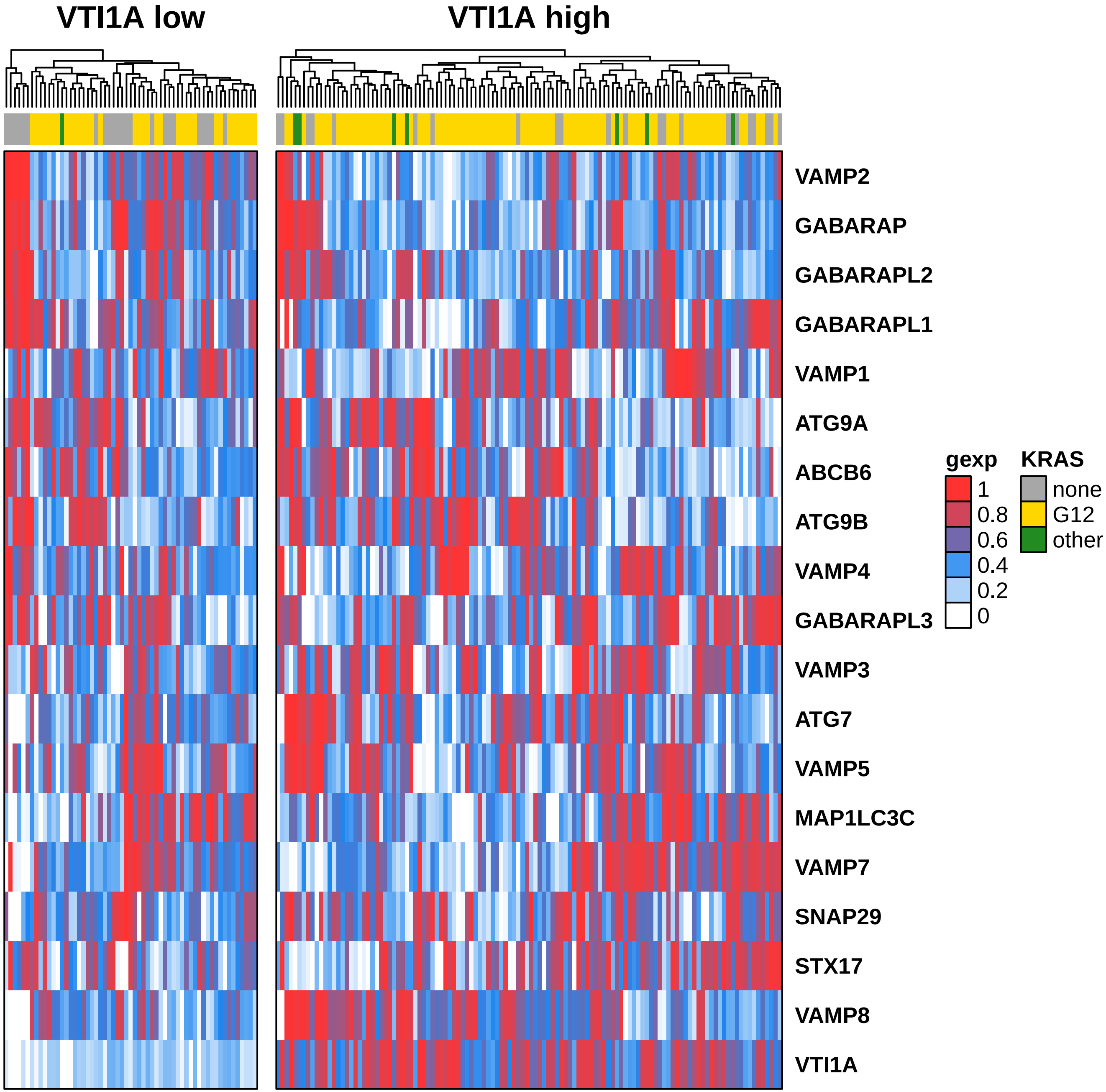
VTI1A low
VTI1A high
VAMP2
GABARAP
GABARAPL2
GABARAPL1
VAMP1
ATG9A
ABCB6
ATG9B
VAMP4
GABARAPL3
VAMP3
ATG7
VAMP5
MAP1LC3C
VAMP7
SNAP29
STX17
VAMP8
VTI1A
gexp
1
0.8
0.6
0.4
0.2
0
KRAS
none
G12
other

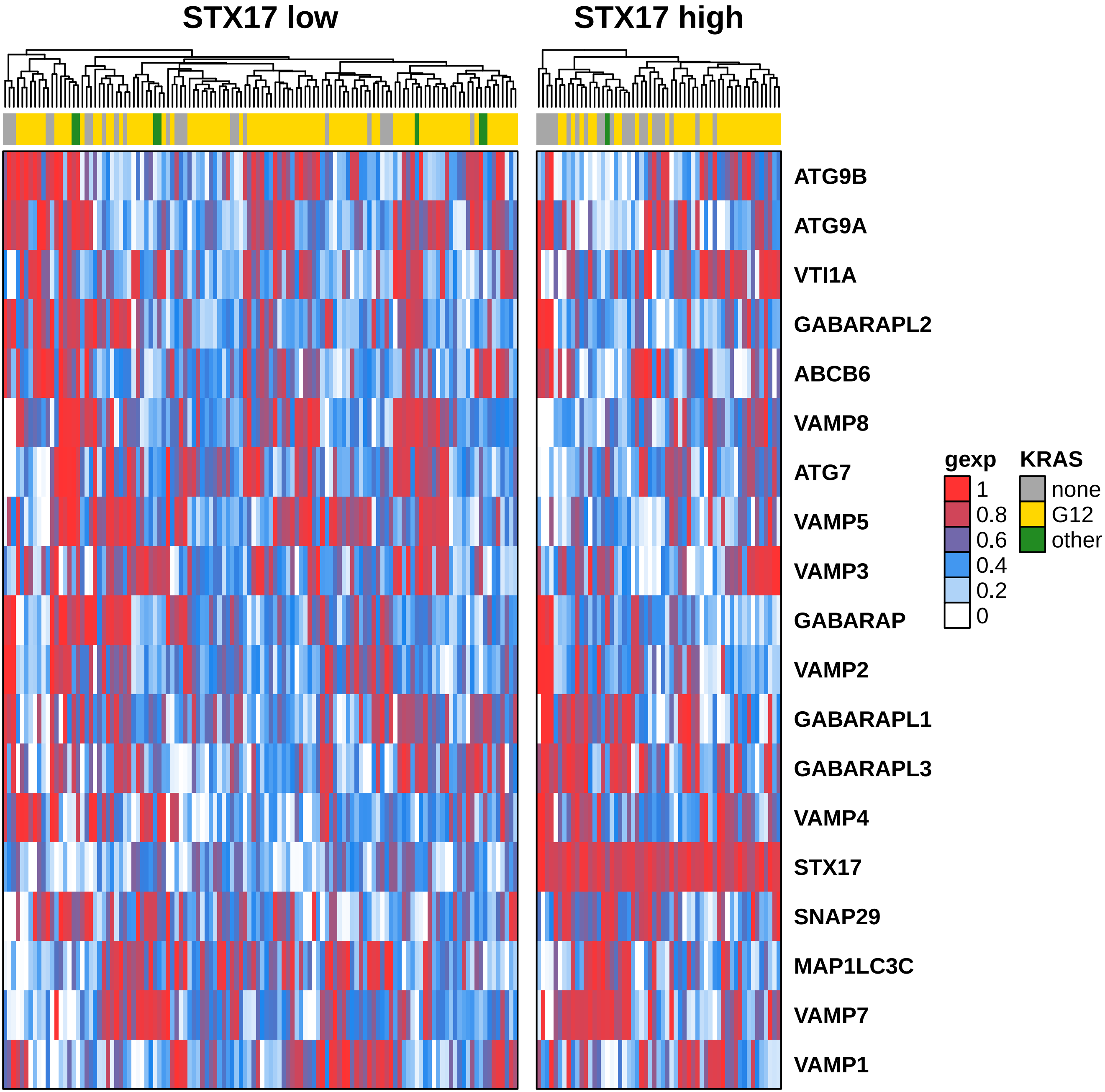
STX17 low
STX17 high
ATG9B
ATG9A
VTI1A
GABARAPL2
ABCB6
VAMP8
ATG7
VAMP5
VAMP3
GABARAP
VAMP2
GABARAPL1
GABARAPL3
VAMP4
STX17
SNAP29
MAP1LC3C
VAMP7
VAMP1
gexp
1
0.8
0.6
0.4
0.2
0
KRAS
none
G12
other

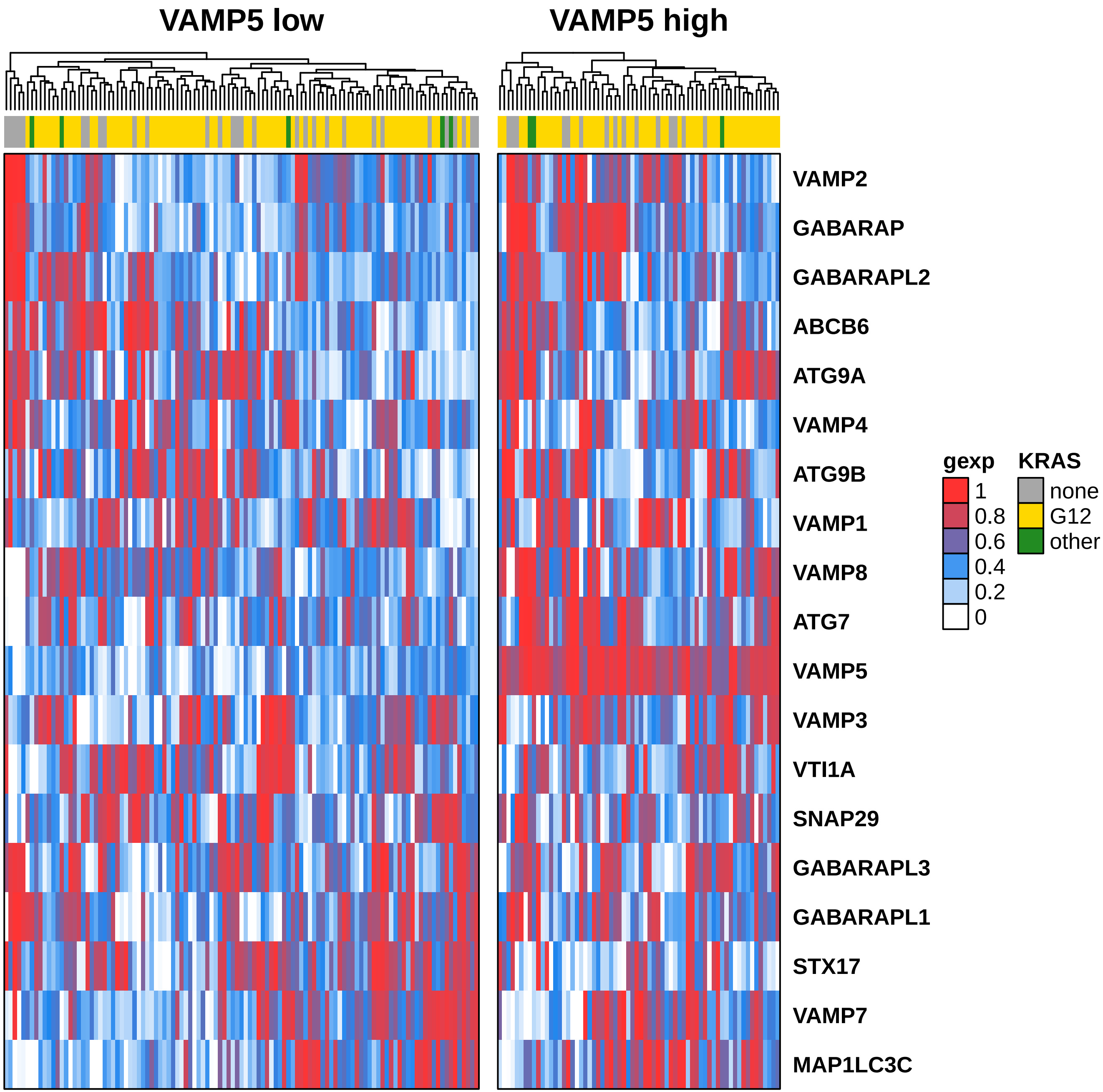

VAMP5 low
VAMP5 high
VAMP2
GABARAP
GABARAPL2
ABCB6
ATG9A
VAMP4
ATG9B
VAMP1
VAMP8
ATG7
VAMP5
VAMP3
VTI1A
SNAP29
GABARAPL3
GABARAPL1
STX17
VAMP7
MAP1LC3C
gexp
1
0.8
0.6
0.4
0.2
0
KRAS
none
G12
other

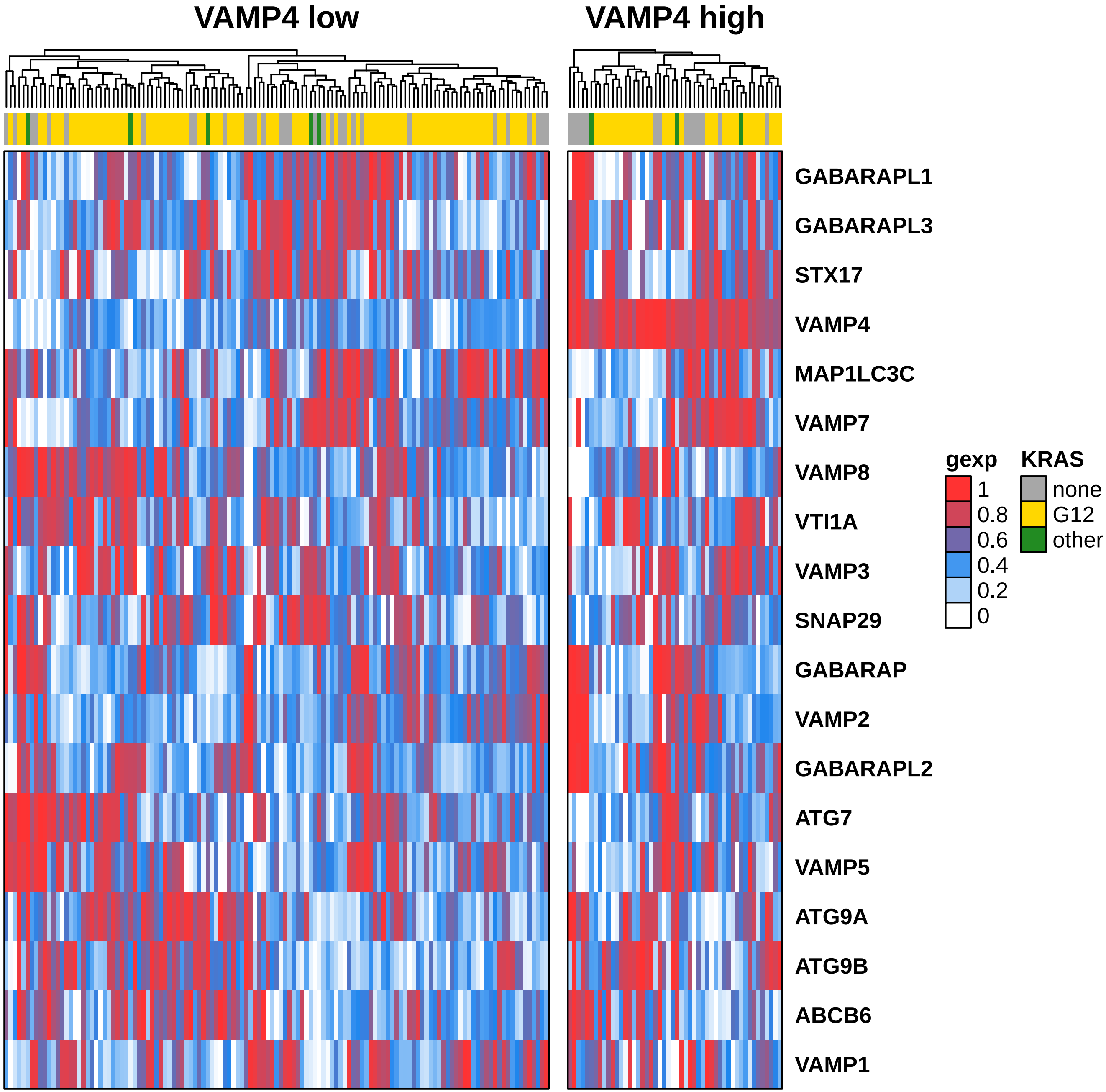
VAMP4 low
VAMP4 high
GABARAPL1
GABARAPL3
STX17
VAMP4
MAP1LC3C
VAMP7
VAMP8
VTI1A
VAMP3
SNAP29
GABARAP
VAMP2
GABARAPL2
ATG7
VAMP5
ATG9A
ATG9B
ABCB6
VAMP1
gexp
1
0.8
0.6
0.4
0.2
0
KRAS
none
G12
other
