# Supplementary material for: Autophagy suppresses Ras-driven epithelial tumourigenesis by limiting the accumulation of reactive oxygen species
Source: Oncogene. 2017 Jun 5;36(40):5576–92. doi: 10.1038/onc.2017.175 (PMC5633656; doi:10.1038/onc.2017.175)
Supplement: Supplementary Data set 2 [file onc2017175x4.pdf]

VAMP2,PAAD LOG1P SCALED bestcutoff 22%

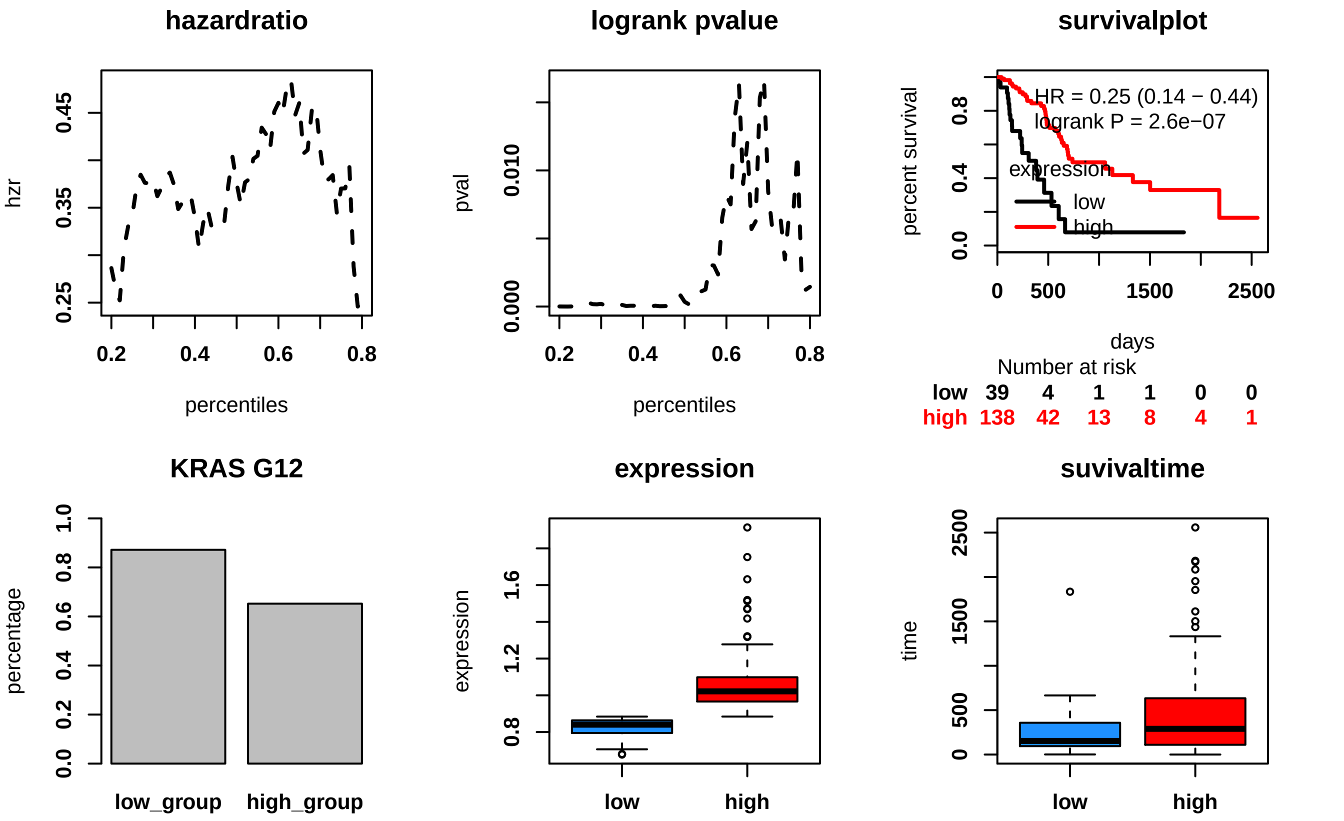

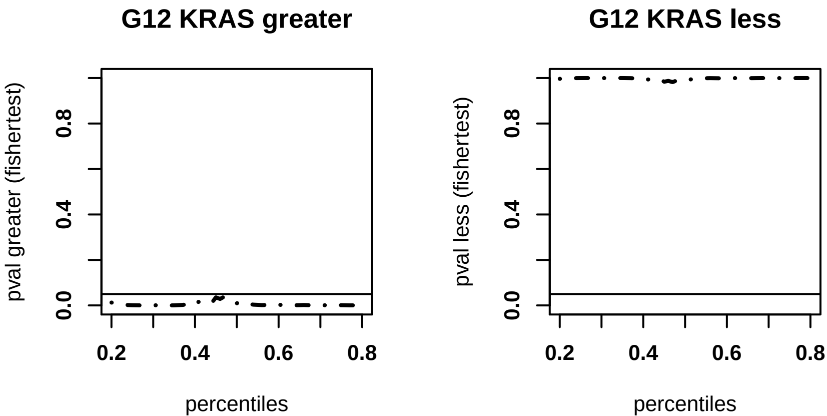

	G12	none/other
low	34	5
high	90	48
pval_less	0.9987	
pval_grea	0.005248	

GABARAP,PAAD LOG1P SCALED bestcutoff 49%

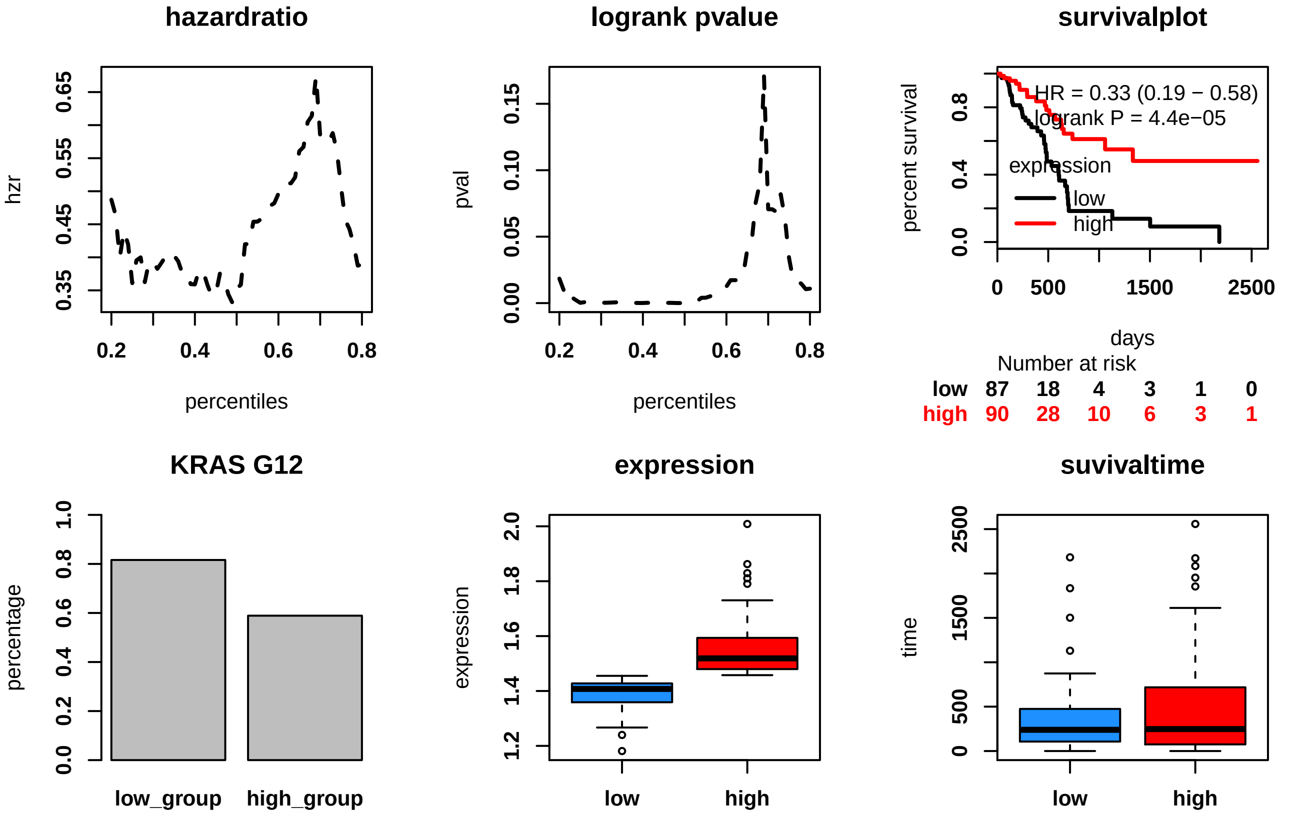

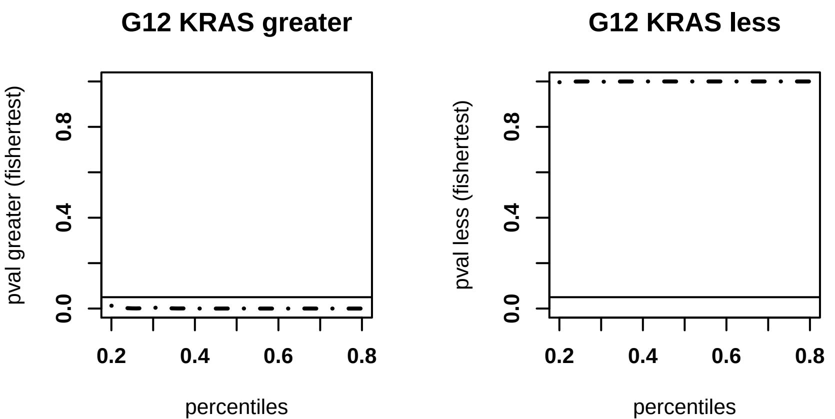

	G12	none/other
low	71	16
high	53	37
pval_less	0.9998	
pval_grea	0.000781	

VAMP3,PAAD LOG1P SCALED bestcutoff 64%

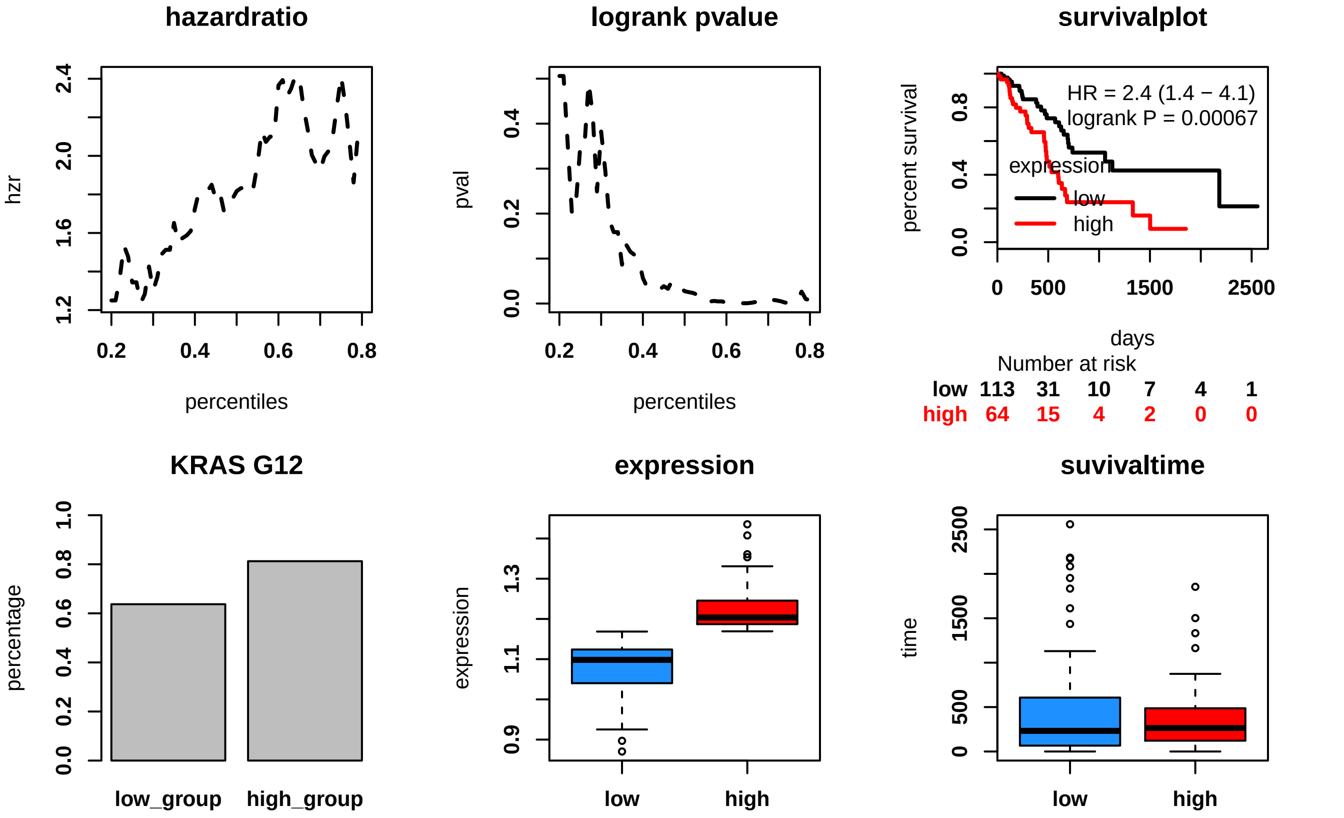

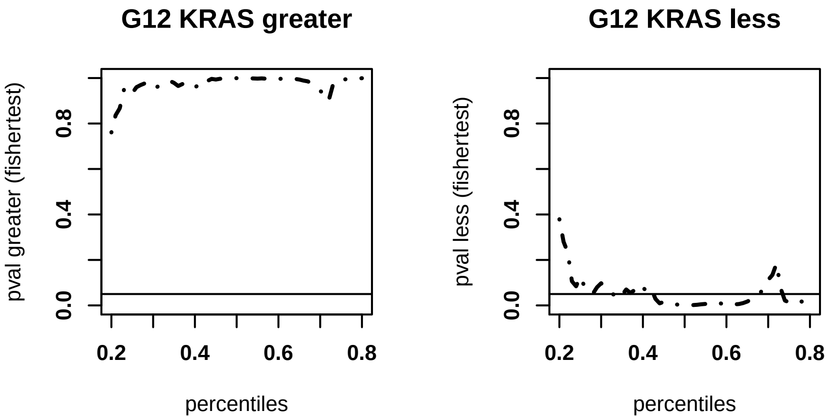

	G12	none/other
low	72	41
high	52	12
pval_less	0.01031	
pval_grea	0.9963	

GABARAPL2,PAAD LOG1P SCALED bestcutoff 80%

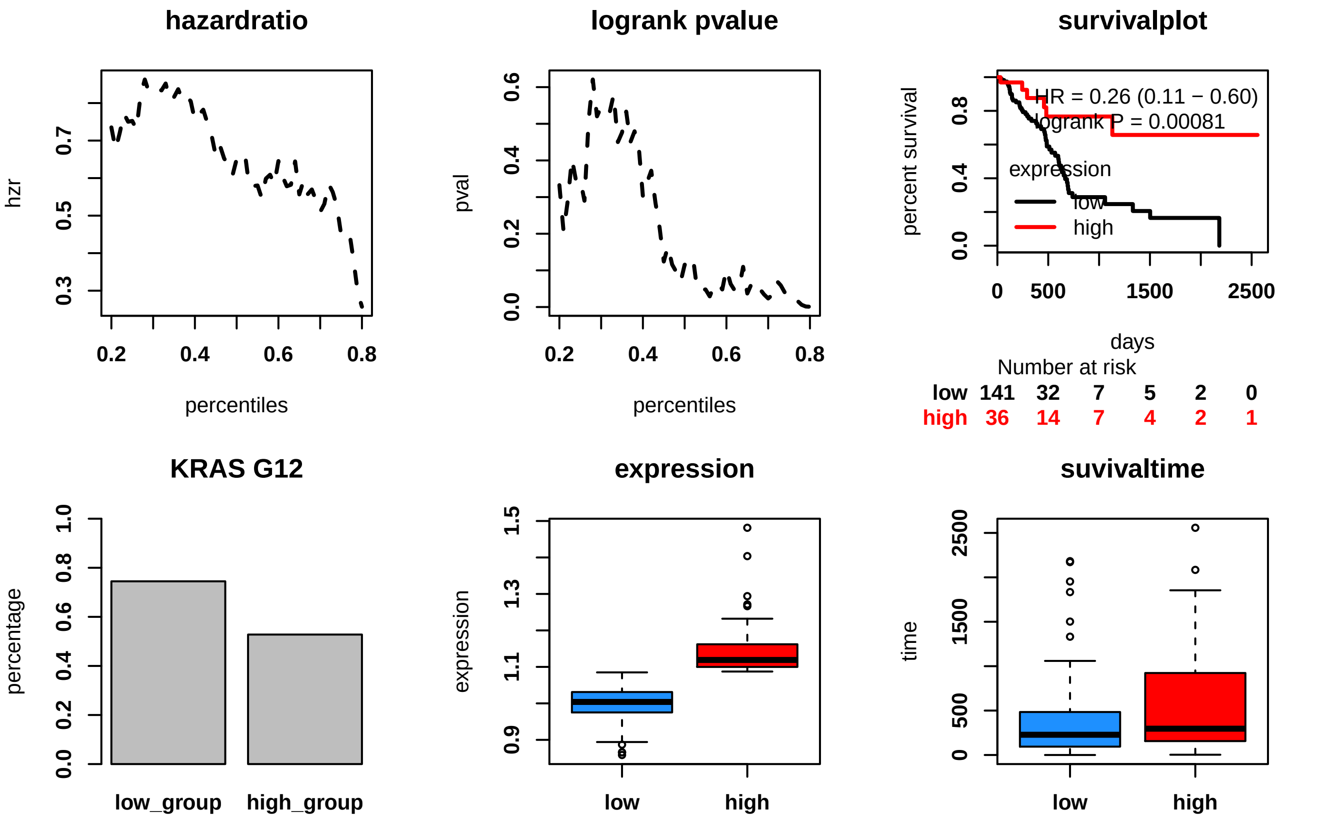

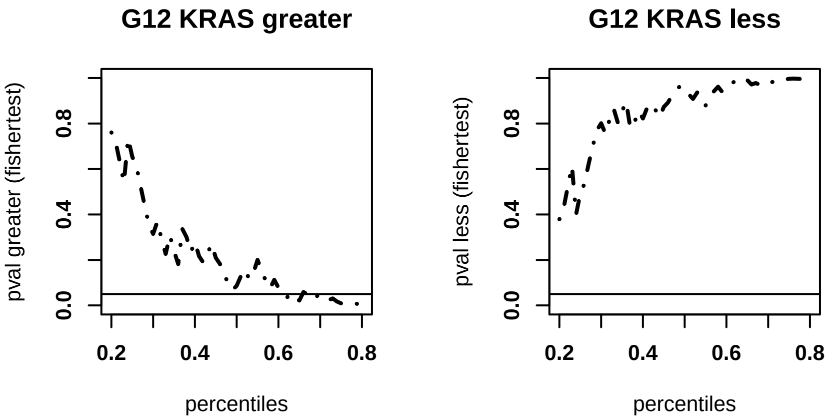

	G12	none/other
low	105	36
high	19	17
pval_less	0.9962	
pval_grea	0.01124	

GABARAPL3,PAAD LOG1P SCALED bestcutoff 66%

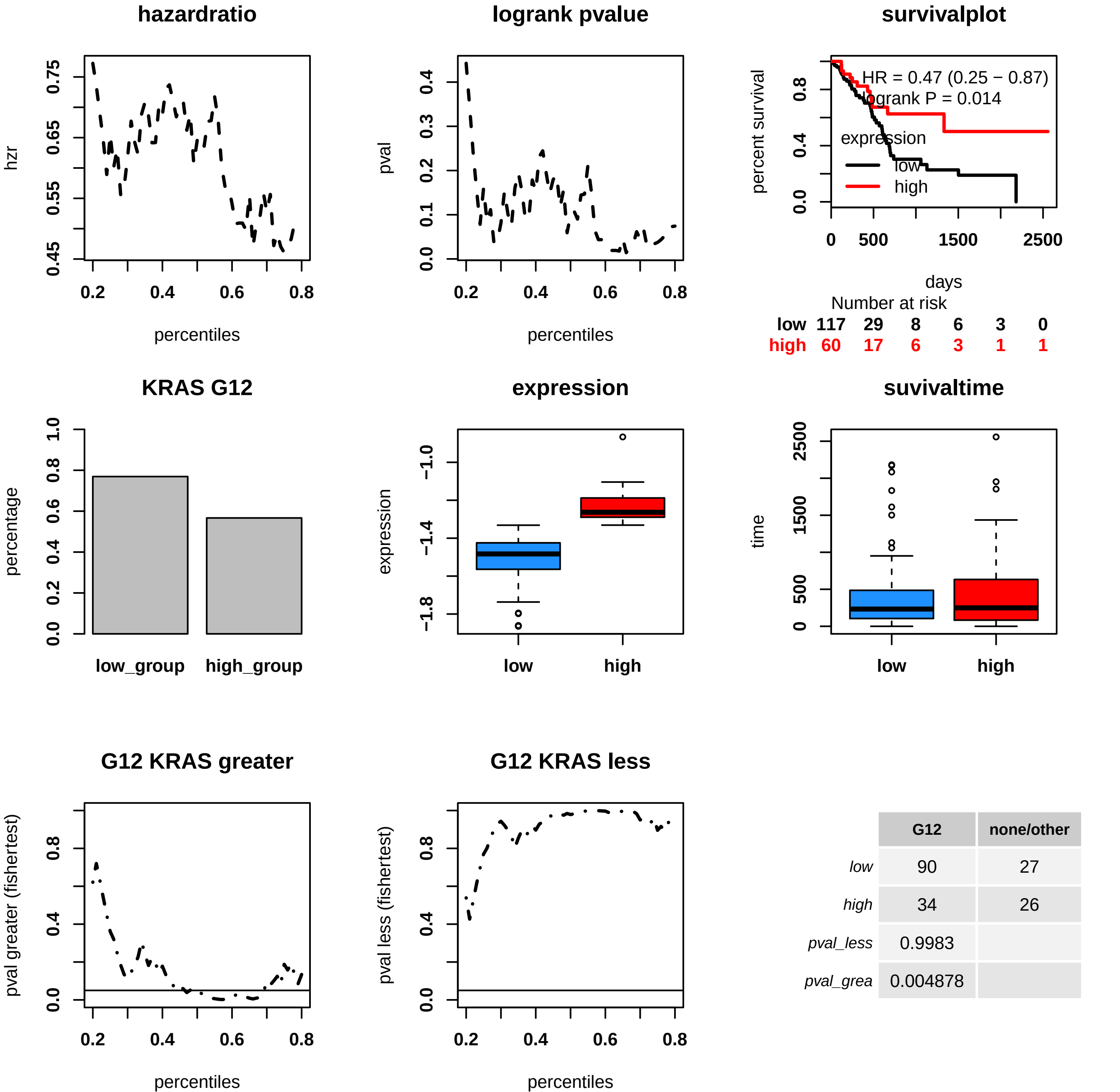

	G12	none/other
low	90	27
high	34	26
pval_less	0.9983	
pval_grea	0.004878	

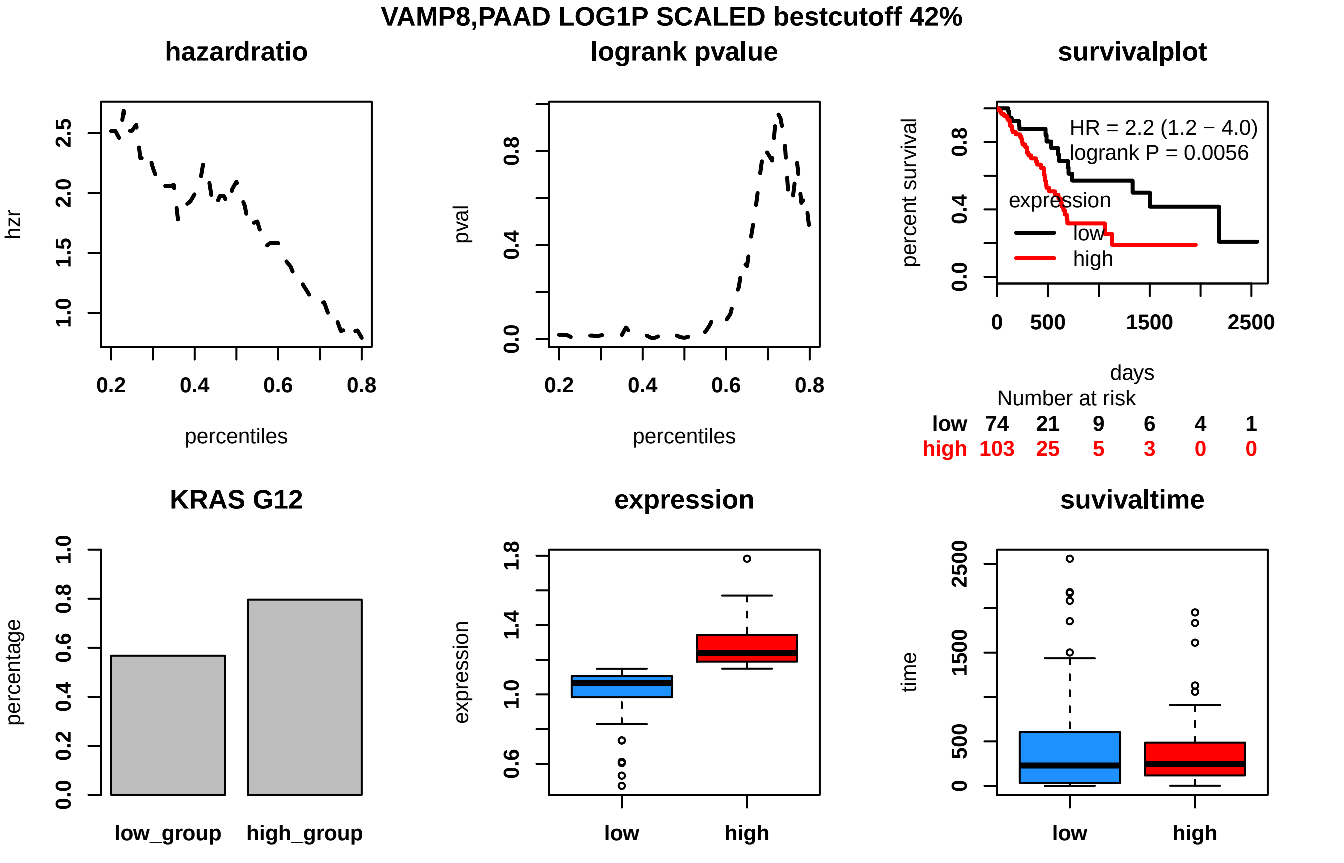

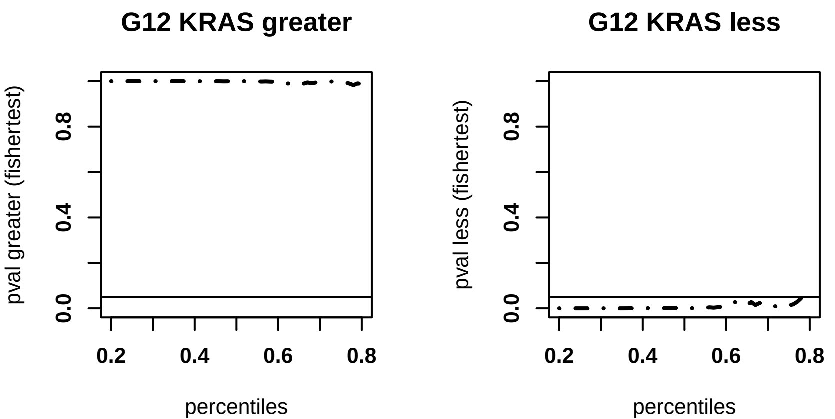

	G12	none/other
low	42	32
high	82	21
pval_less	0.0009688	
pval_grea	0.9997	

VAMP1,PAAD LOG1P SCALED bestcutoff 34%

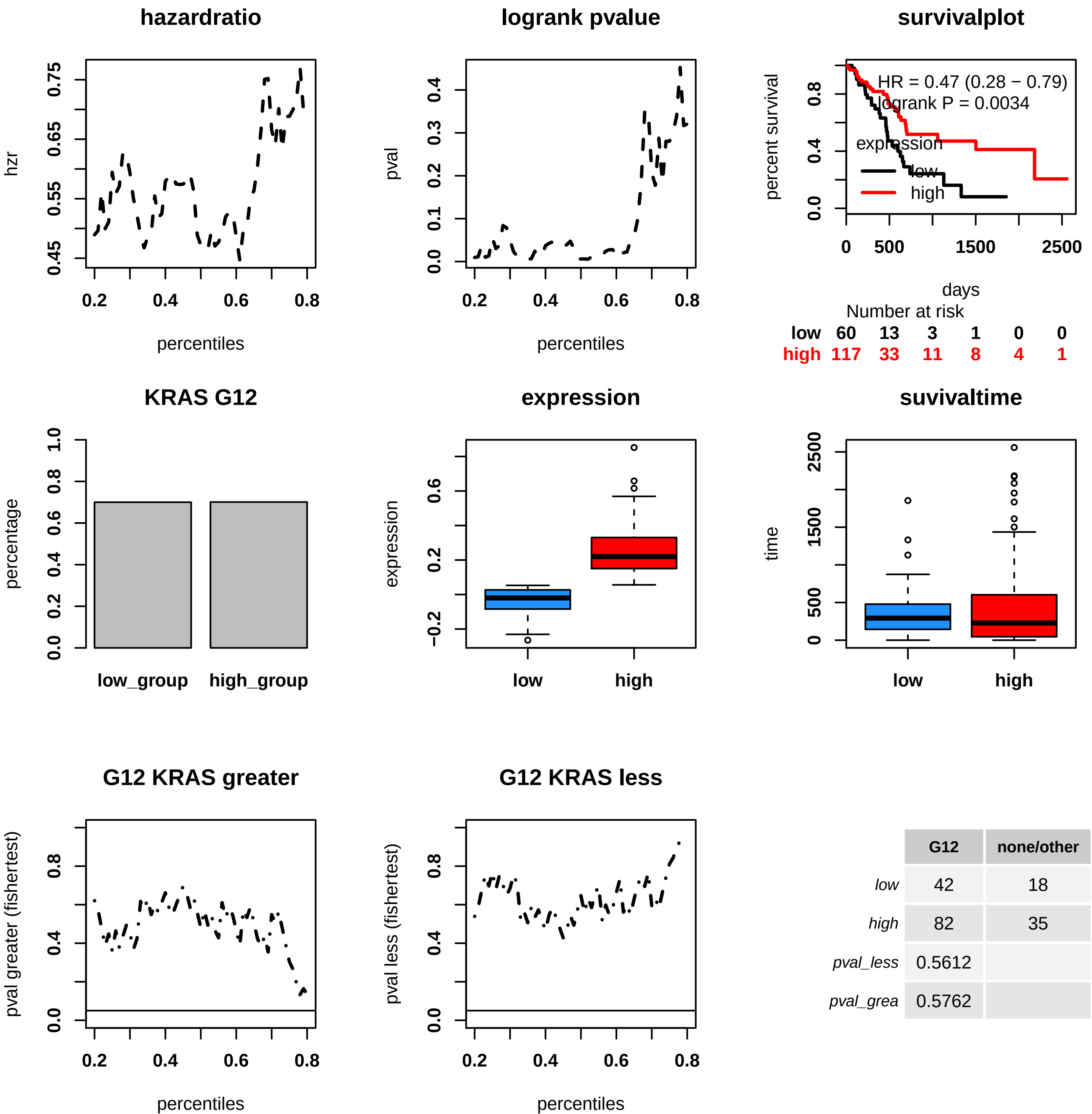

	G12	none/other
low	42	18
high	82	35
pval_less	0.5612	
pval_grea	0.5762	

SNAP29,PAAD LOG1P SCALED bestcutoff 20%

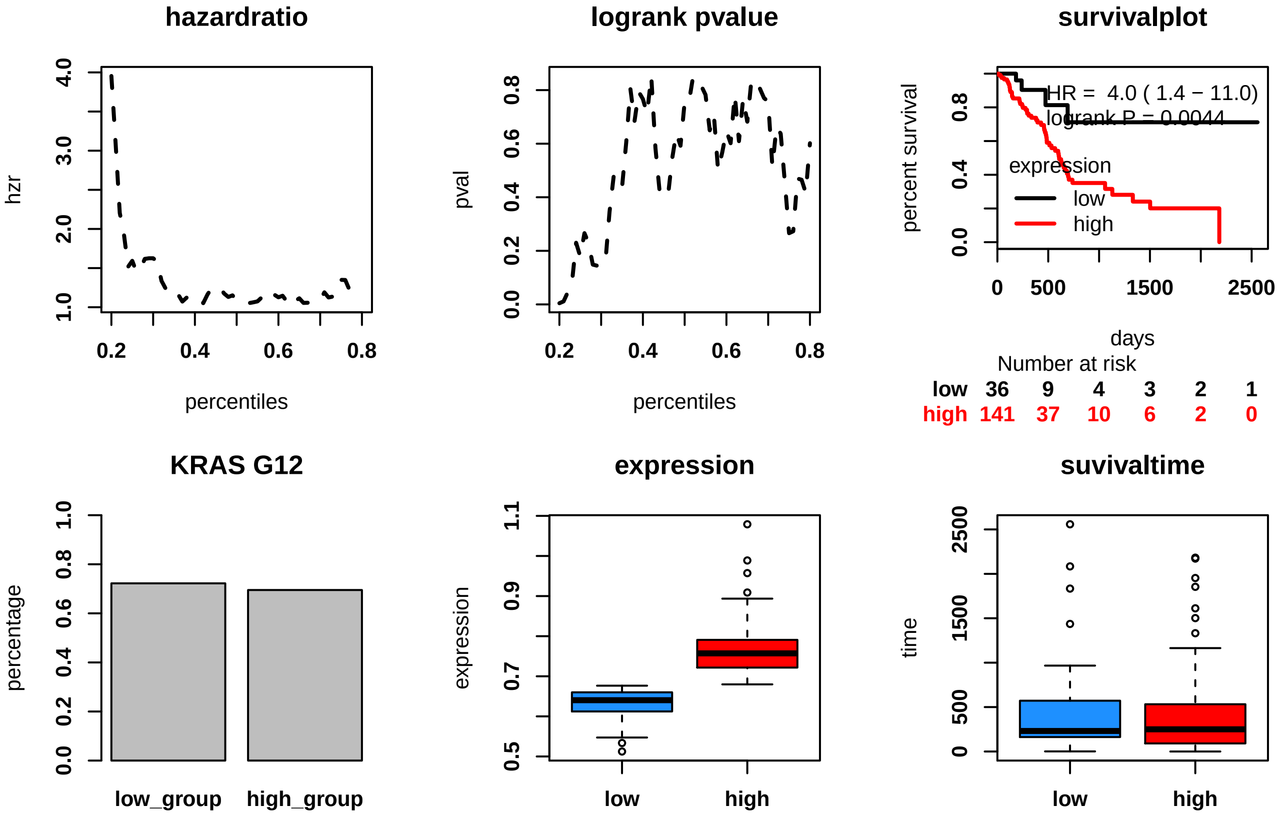

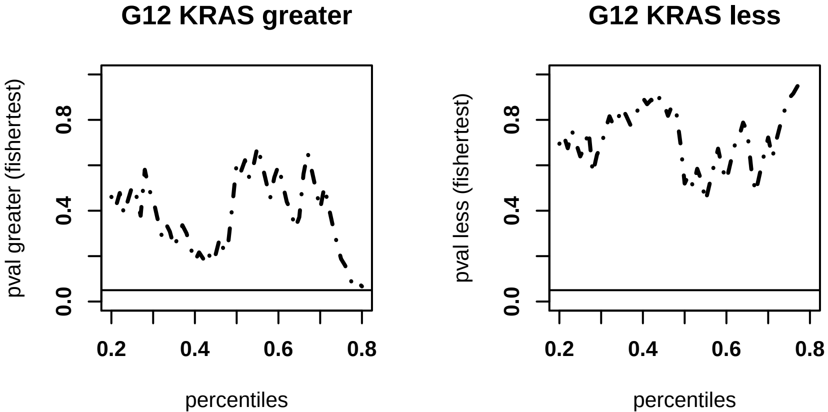

	G12	none/other
low	26	10
high	98	43
pval_less	0.6946	
pval_grea	0.4612	

VAMP7,PAAD LOG1P SCALED bestcutoff 20%

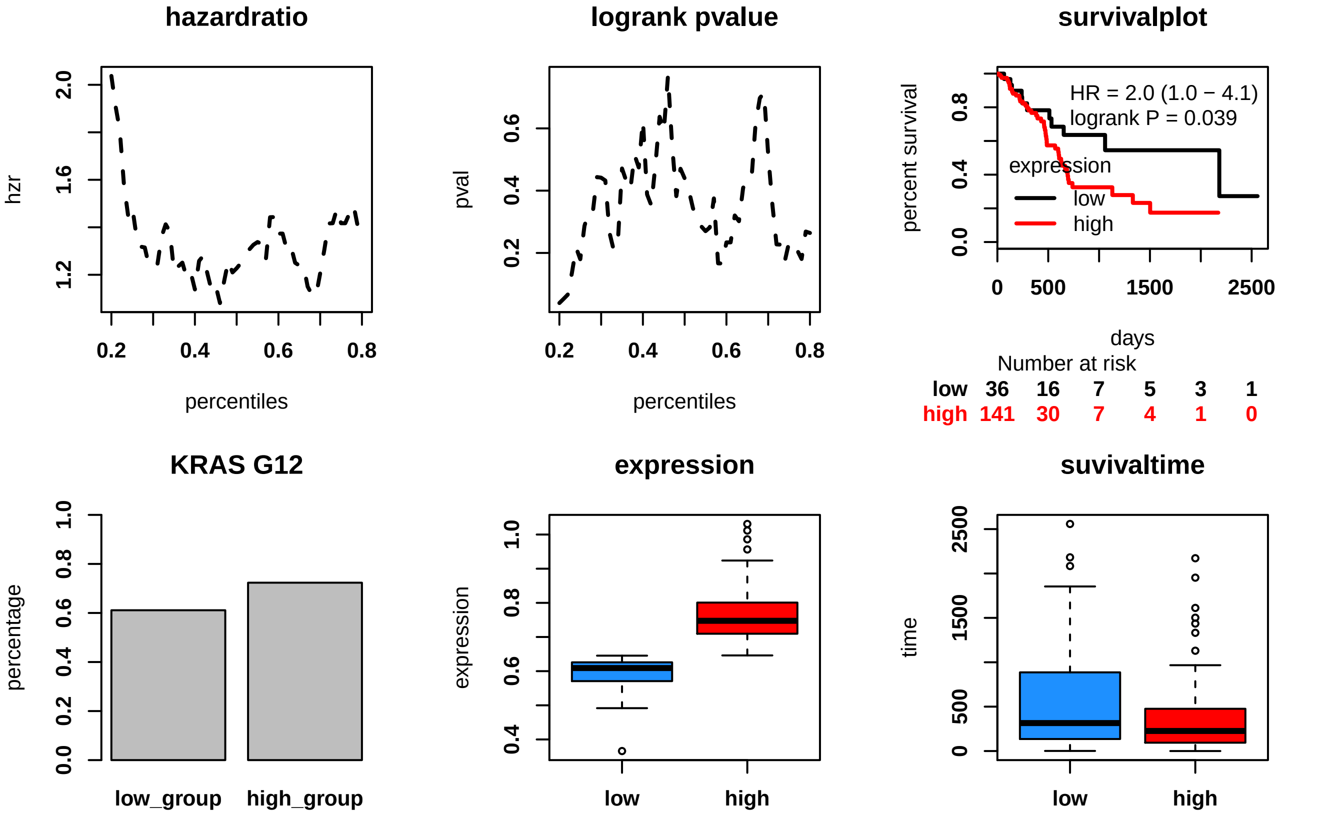

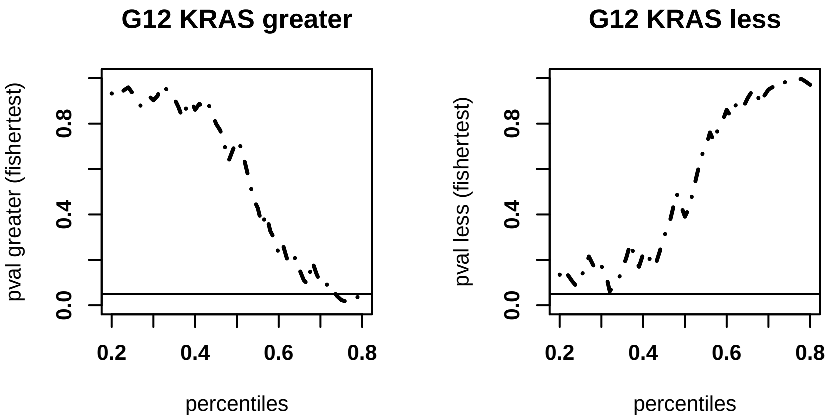

	G12	none/other
low	22	14
high	102	39
pval_less	0.1343	
pval_grea	0.9333	

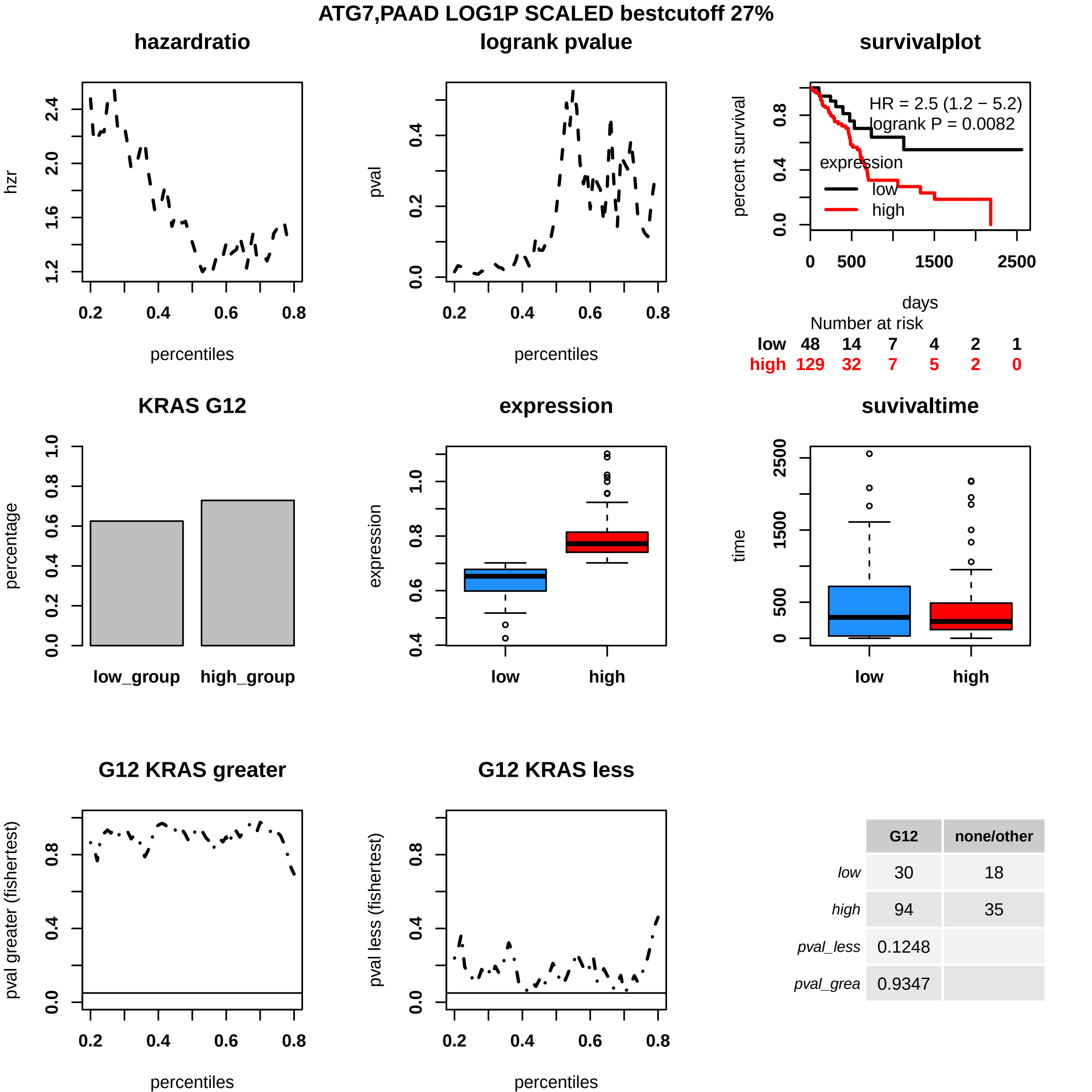

	G12	none/other
low	30	18
high	94	35
pval_less	0.1248	
pval_grea	0.9347	

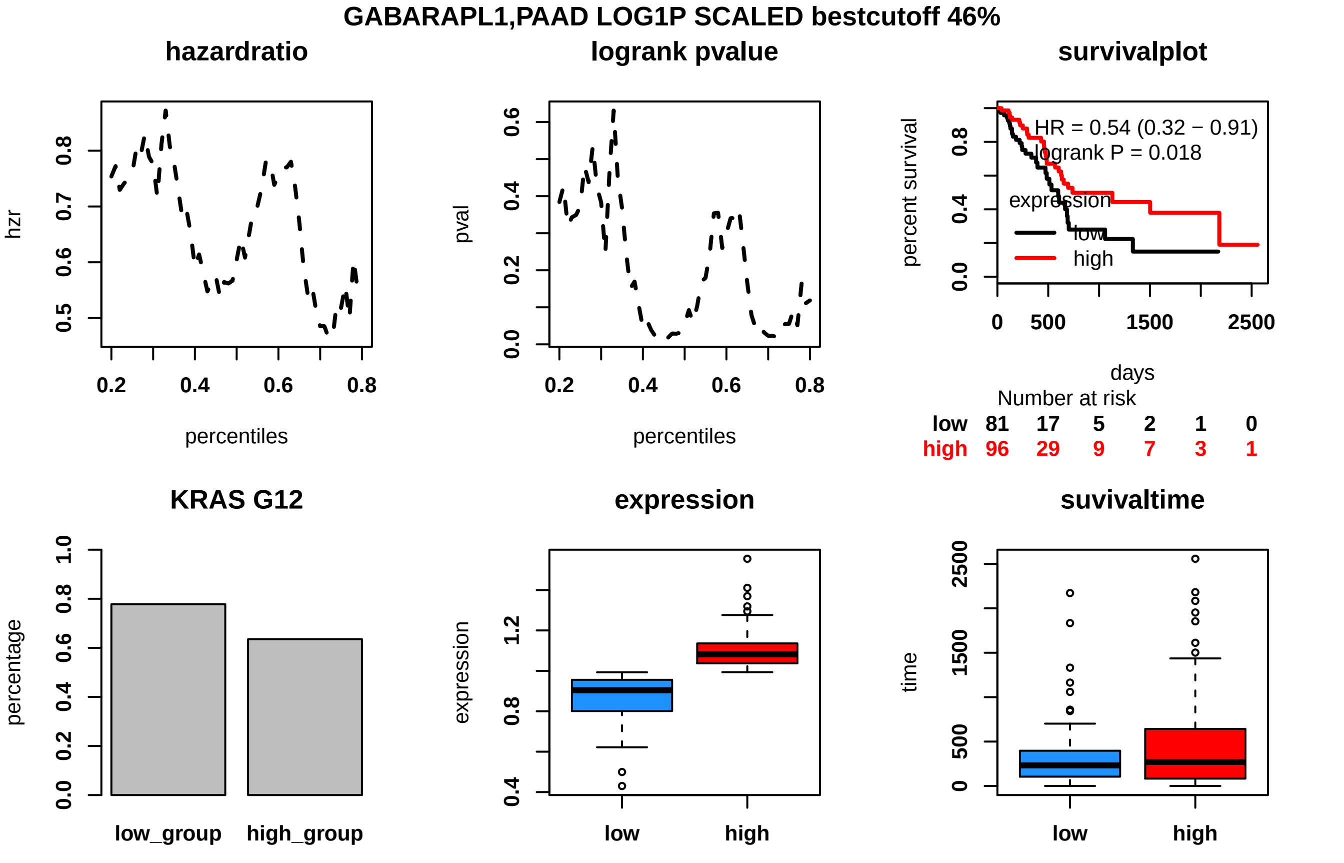

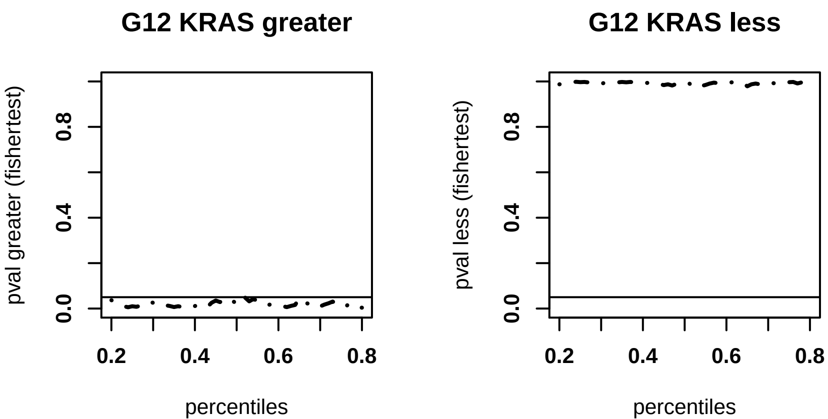

	G12	none/other
low	63	18
high	61	35
pval_less	0.9875	
pval_grea	0.02841	

ATG9B,PAAD LOG1P SCALED bestcutoff 21%

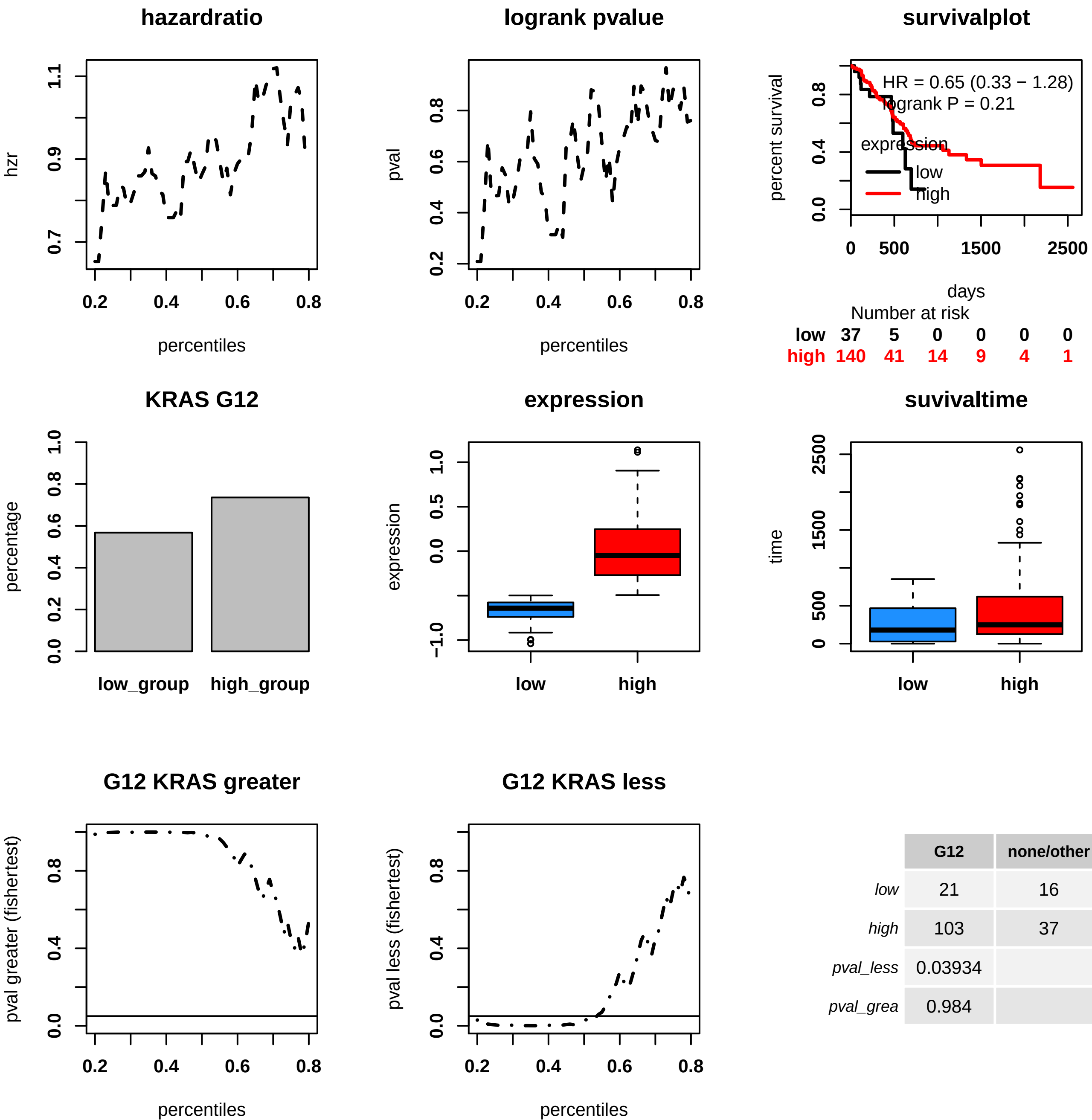

	G12	none/other
low	21	16
high	103	37
pval_less	0.03934	
pval_grea	0.984	

ATG9A,PAAD LOG1P SCALED bestcutoff 74%

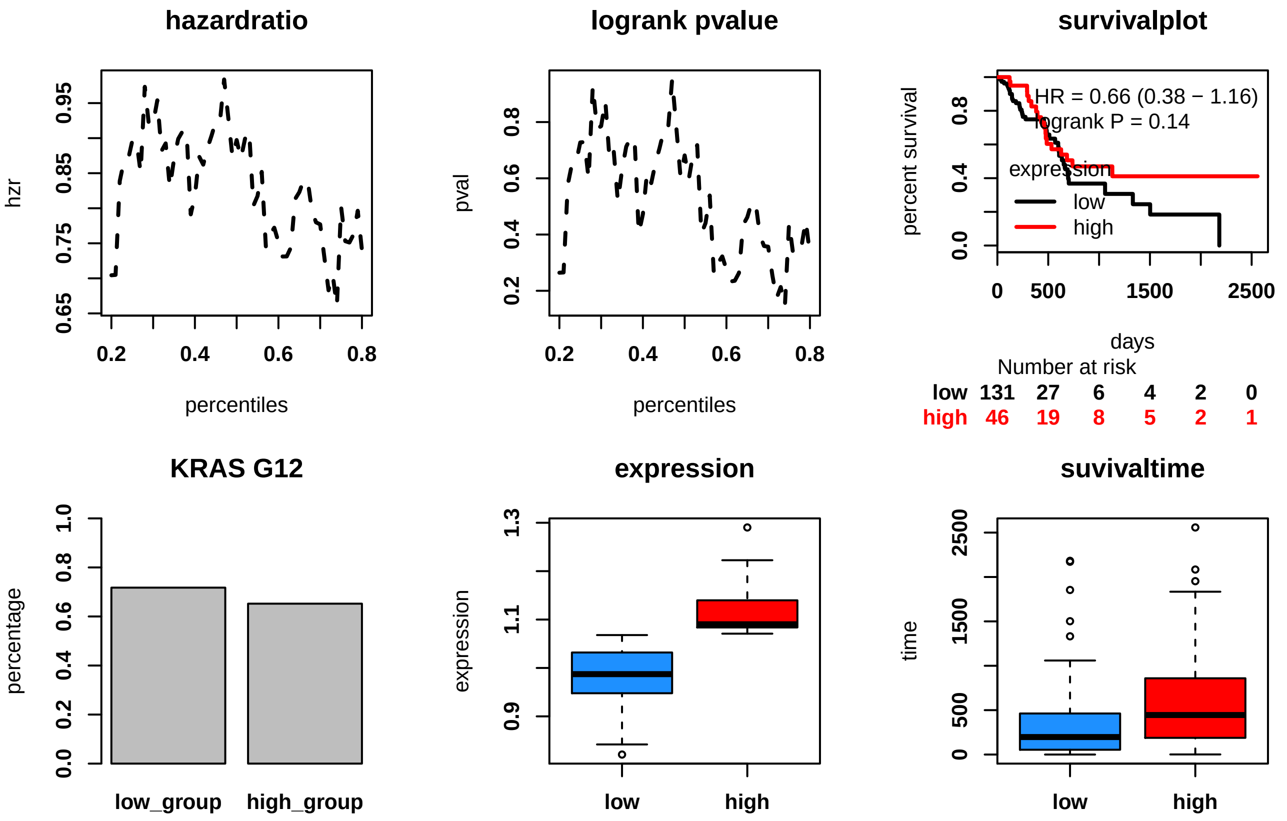

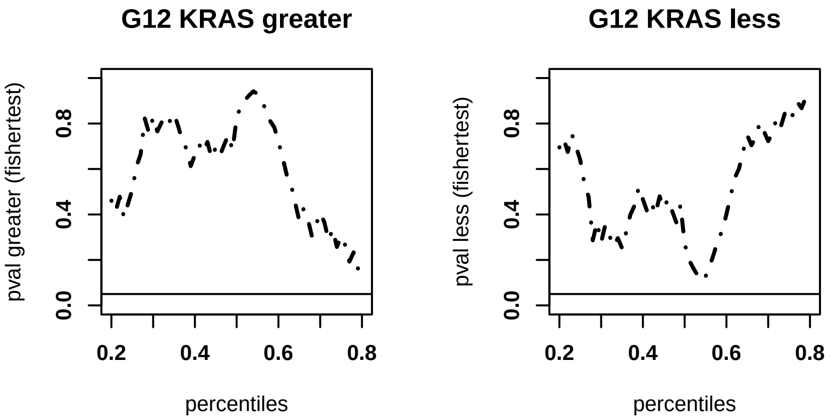

	G12	none/other
low	94	37
high	30	16
pval_less	0.8461	
pval_grea	0.257	

MAP1LC3C,PAAD LOG1P SCALED bestcutoff 53%

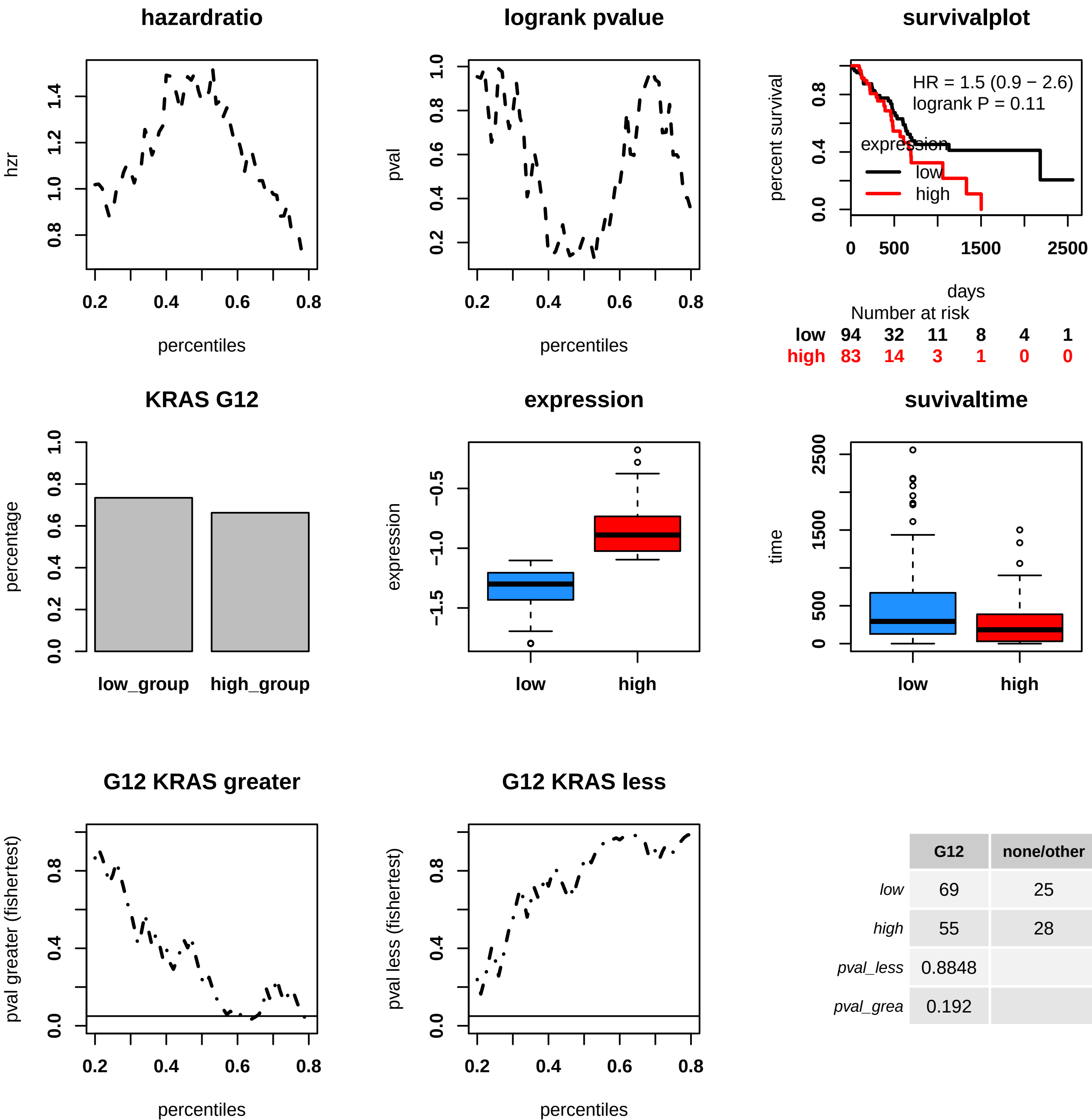

	G12	none/other
low	69	25
high	55	28
pval_less	0.8848	
pval_grea	0.192	

ABCB6,PAAD LOG1P SCALED bestcutoff 73%

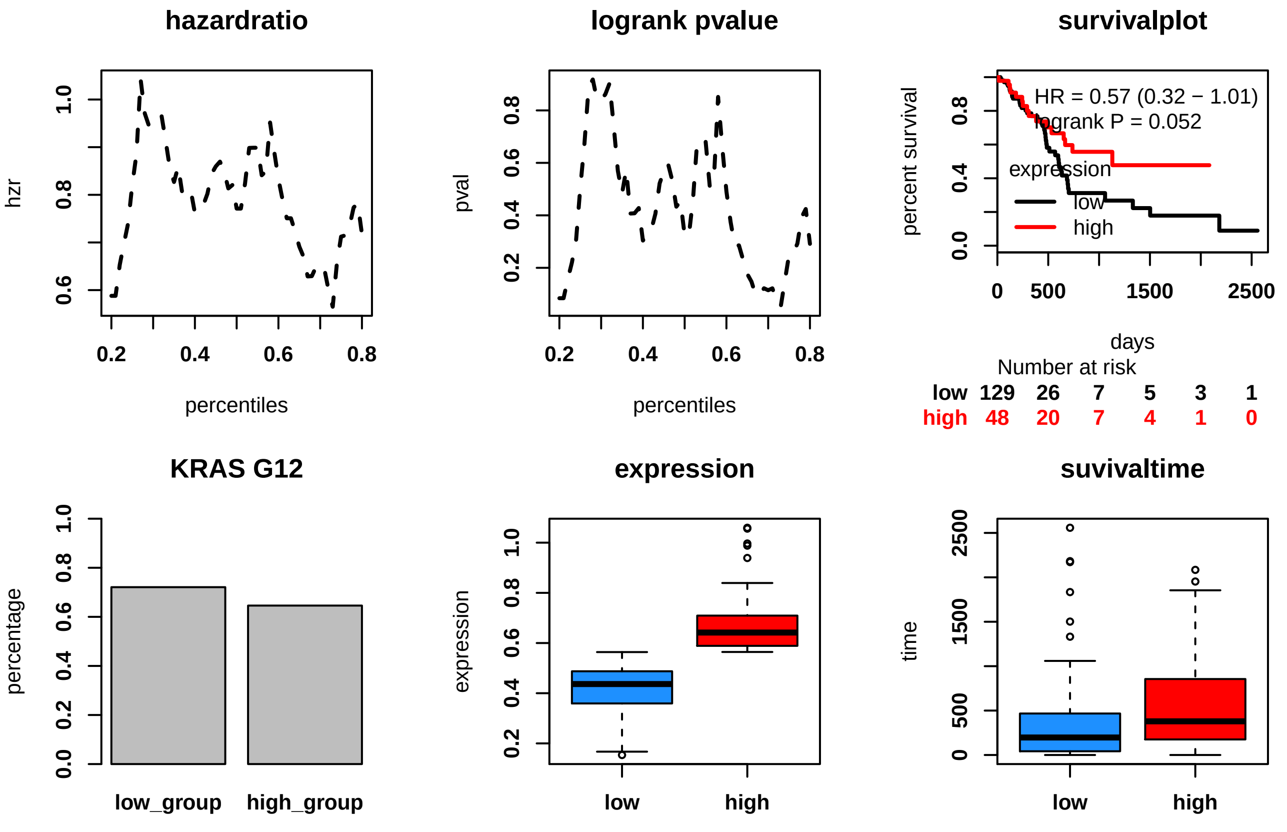

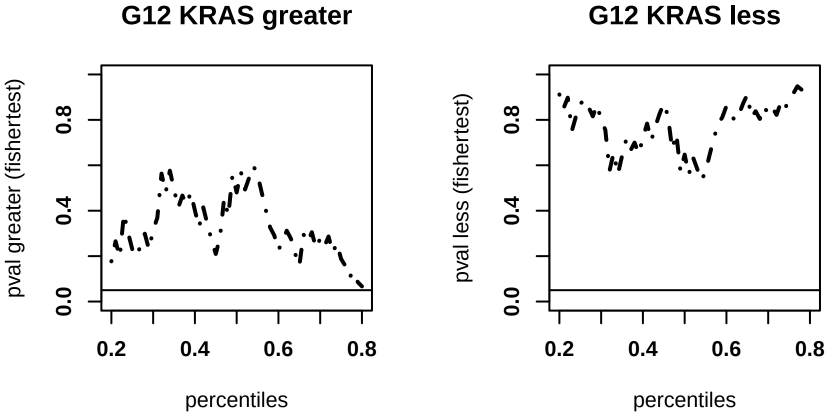

	G12	none/other
low	93	36
high	31	17
pval_less	0.8752	
pval_grea	0.215	

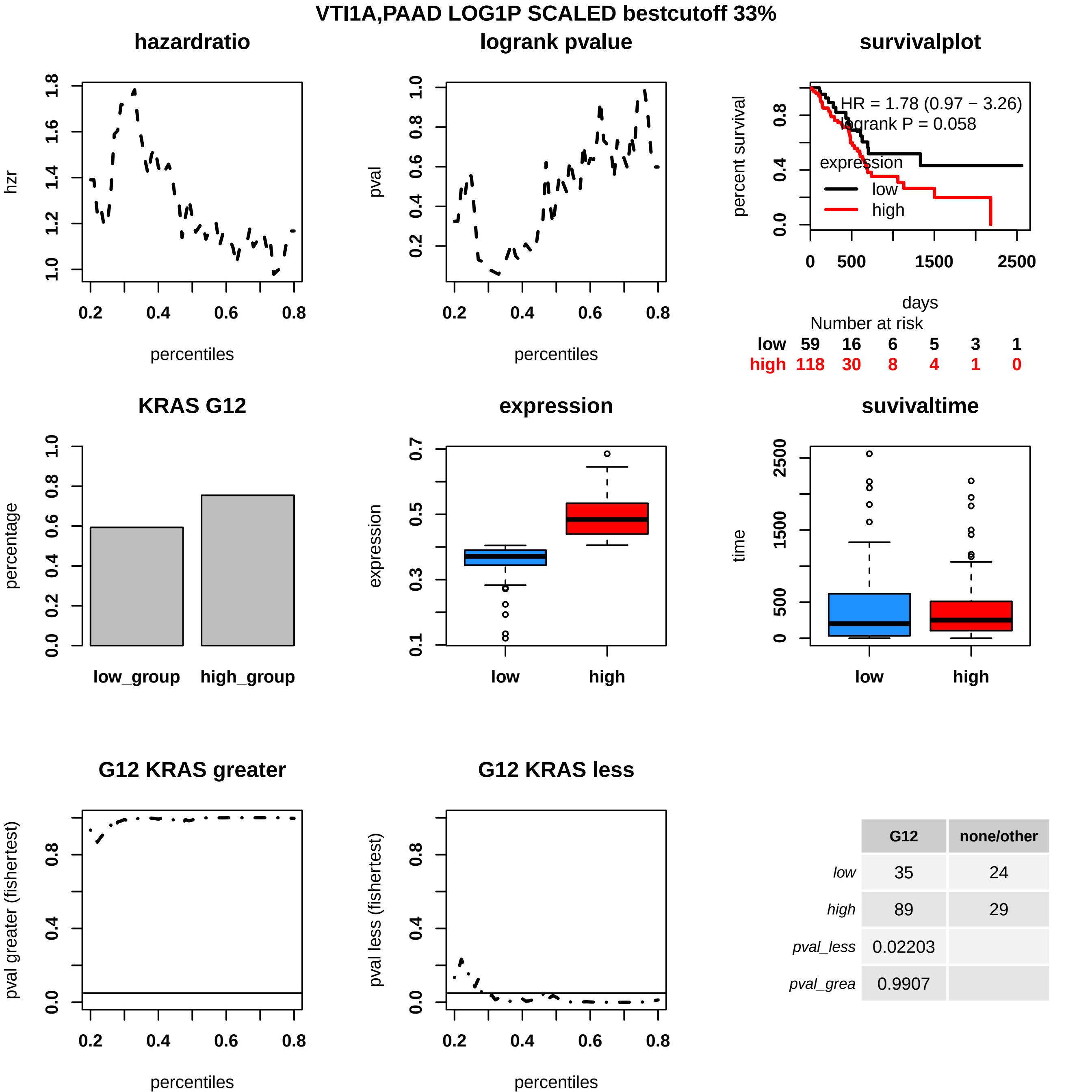

	G12	none/other
low	35	24
high	89	29
pval_less	0.02203	
pval_grea	0.9907	

STX17,PAAD LOG1P SCALED bestcutoff 68%

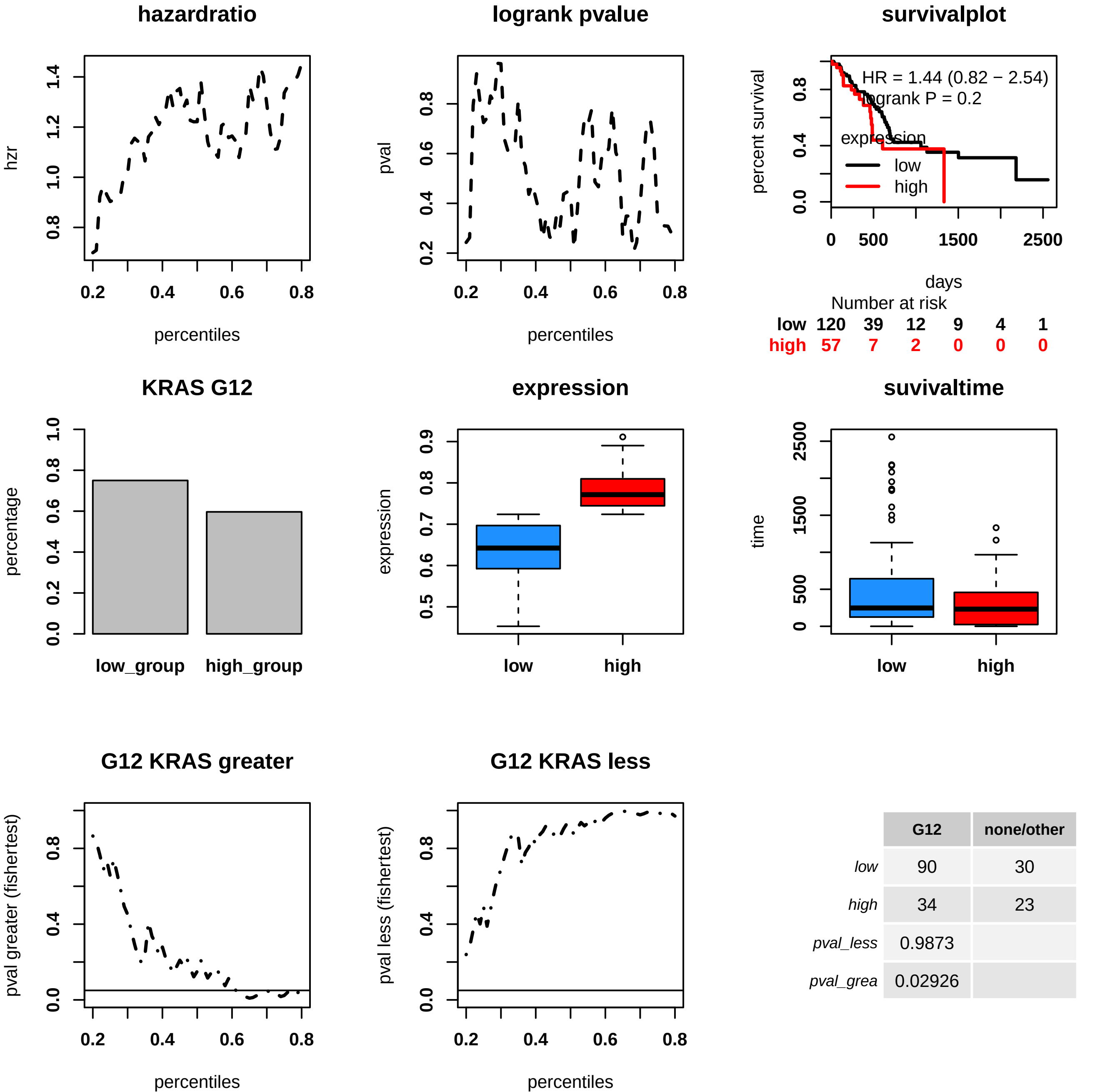

	G12	none/other
low	90	30
high	34	23
pval_less	0.9873	
pval_grea	0.02926	

VAMP5,PAAD LOG1P SCALED bestcutoff 63%

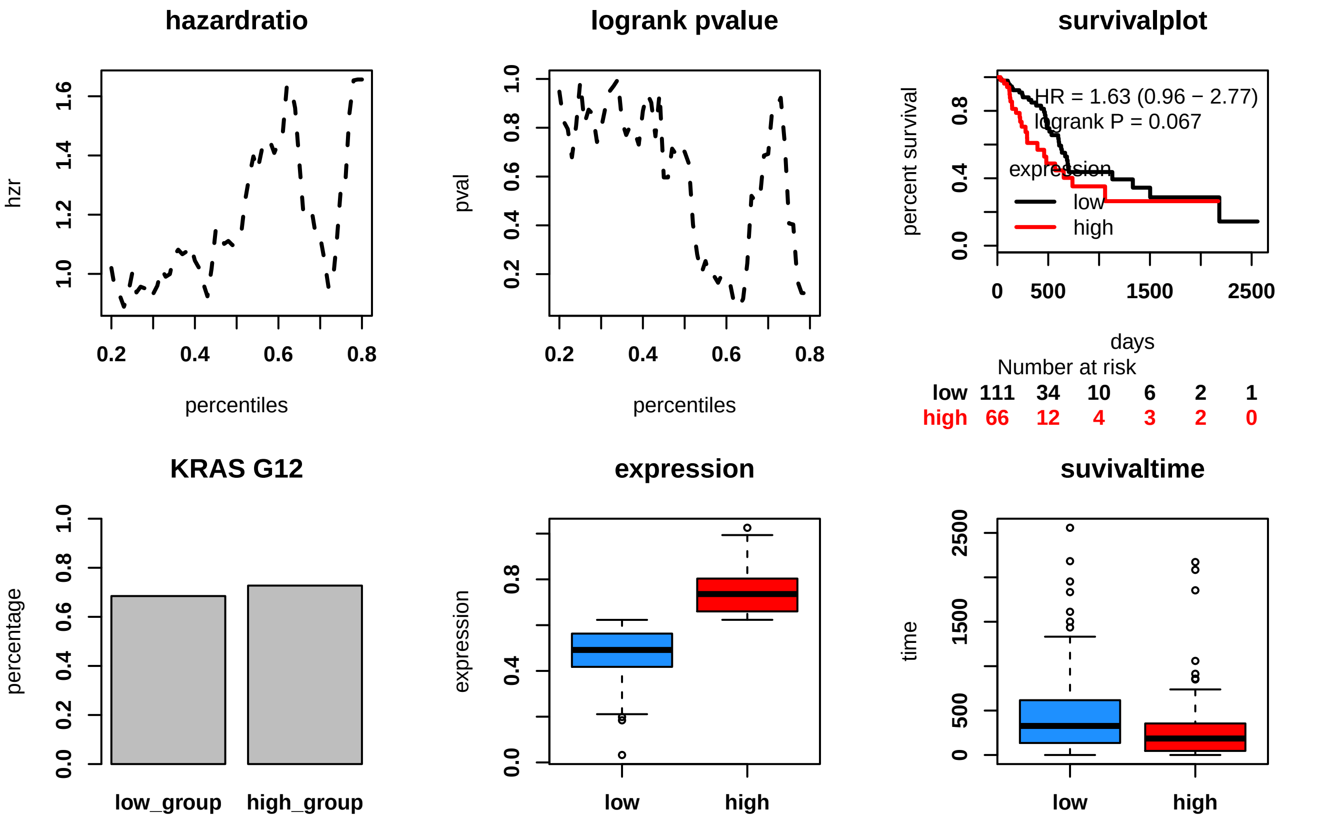

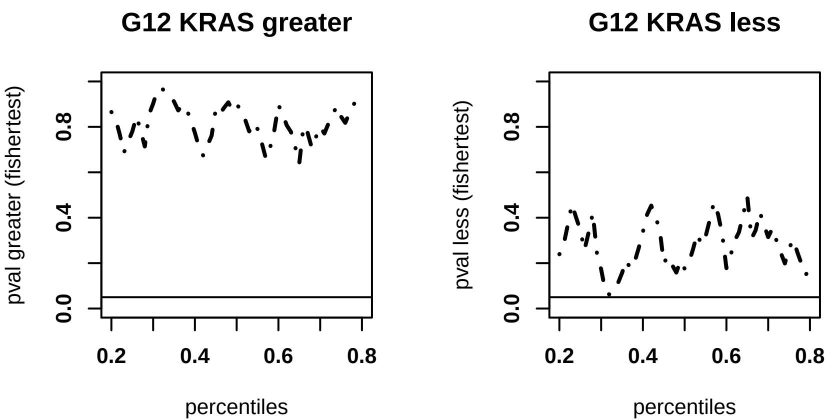

	G12	none/other
low	76	35
high	48	18
pval_less	0.336	
pval_grea	0.7779	

VAMP4,PAAD LOG1P SCALED bestcutoff 72%

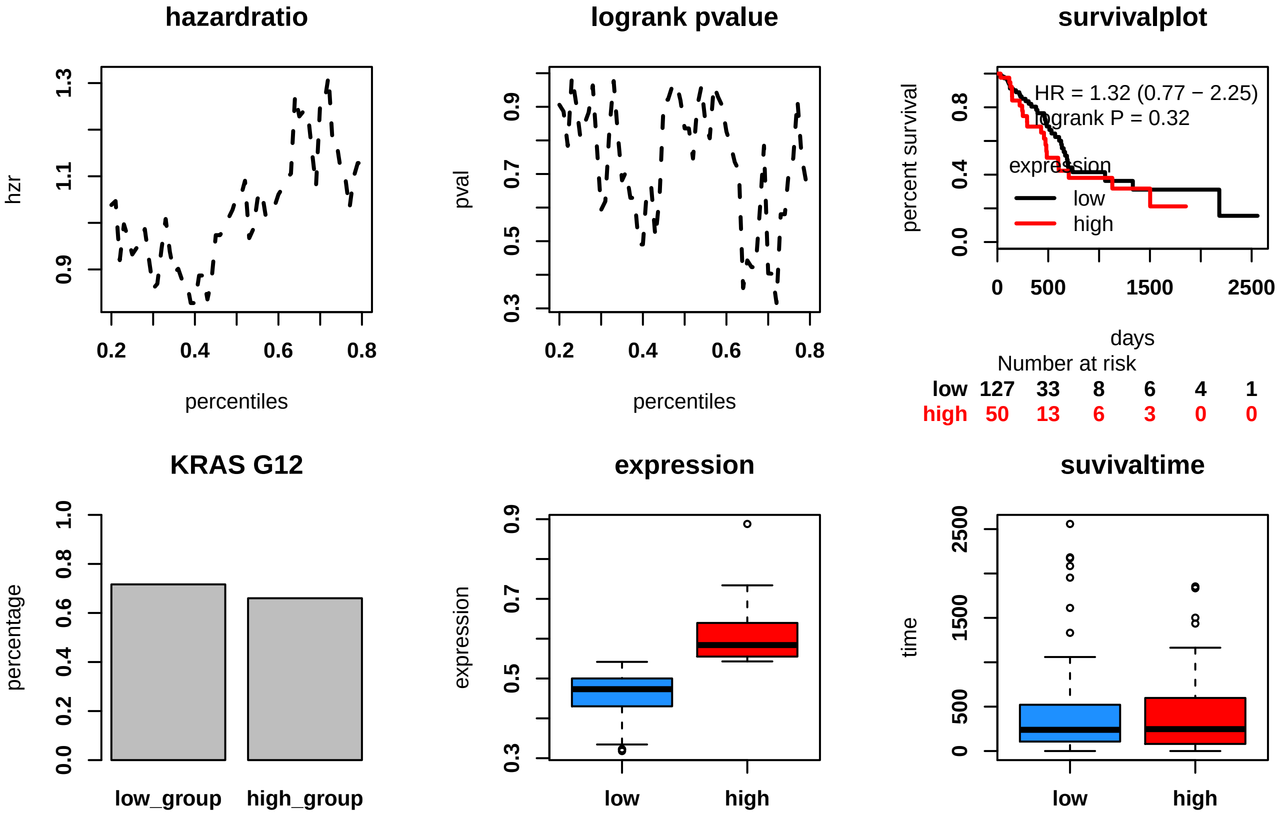

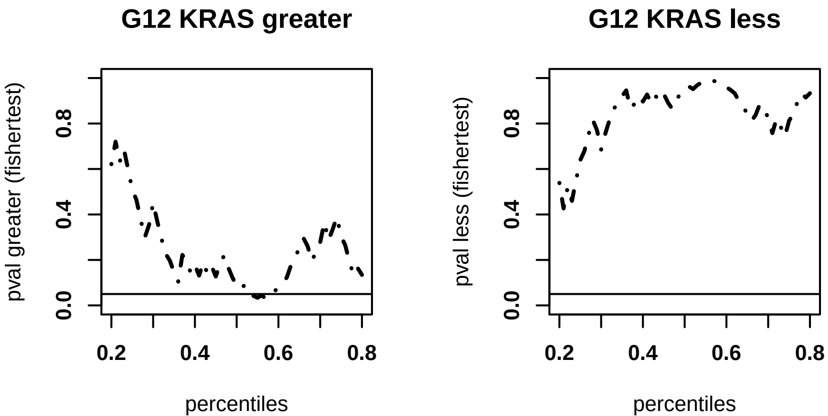

	G12	none/other
low	91	36
high	33	17
pval_less	0.822	
pval_grea	0.2864	
